# Longitudinal study of SARS-CoV-2 infections in different employee groups of long distance train services from June 2020 until February 2021 in Germany

**DOI:** 10.1017/S095026882200070X

**Published:** 2022-04-20

**Authors:** HyoungJin Kim, Robert Schultz-Heienbrok, Markus Uhle, Jenni Neubert, Fabian Ball, Matthes Metz, Christian Gravert

**Affiliations:** 1 Charité Research Organisation GmbH, Berlin, Germany; 2 Janssen-Cilag GmbH, Neuss, Germany; 3 DB Fernverkehr AG, Frankfurt, Germany; 4 Department of Biostatistics, GCP-Service International Ltd. & Co. KG, Bremen, Germany; 5 Deutsche Bahn AG, Berlin, Germany

**Keywords:** COVID-19, public transport, seroepidemiological study, severe acute respiratory syndrome coronavirus 2

## Abstract

This prospective longitudinal epidemiological study was aimed at investigating the occupational SARS-CoV-2 infection risk of long distance train services in Germany. Three different employee groups (train attendants, train drivers and maintenance workers) within the workforce of the German railway carrier Deutsche Bahn Fernverkehr AG were studied based on their contact frequency with passengers and colleagues. Approximately 1100 employees were tested by PCR for acute infections and by antibody detection for past infections in June 2020, October 2020 and February 2021. Cumulative incidence (acute and past infections) after the third (final) test series in February 2021 was 8.5% (95% interval CI 6.8–10.4): 8.5% (95% CI 6.2–11.2) for train attendants, 5.5% (95% CI 2.9–9.5) for train drivers and 11.8% (95% CI 7.6–17.2) for maintenance workers. Between June 2020 and October 2020, the incidence was 1.2% (95% CI 0.6–2.3): 1.2% (95% CI 0.4–2.7) for train attendants, 1.1% (95% CI 0.1–3.9) for train drivers and 1.4% (95% CI 0.17–5.10) for maintenance workers. Between October 2020 and February 2021, it was 5.1% (95% CI 3.6–6.8): 5.2% (95% CI 3.3–7.8) for train attendants, 1.6% (95% CI 0.3–4.5) for train drivers and 8.8% (95% CI 4.9–14.3) for maintenance workers. Thus, contrary to expectation our exploratory data did not show train attendants to be at the highest risk of SARS-CoV-2 infections among the employee groups. In line with expectations, train drivers, representing the low contact group, seemed at lowest occupational risk.

## Introduction

SARS-CoV-2 (severe acute respiratory syndrome coronavirus 2) is a novel coronavirus that causes the respiratory disease COVID-19 (coronavirus disease 2019). It first appeared in December 2019 in the metropolis of Wuhan, China and led to a pandemic within a few months. In Germany, everyday life has been affected by SARS-CoV-2 since spring 2020.

The clinical course of COVID-19 is non-specific and ranges from asymptomatic, mild symptoms (e.g. dry cough, fever) to severe pneumonia with respiratory failure and death.

Since asymptomatic and mild courses result in a high number of unreported cases, it is still difficult to determine the actual number of SARS-CoV-2 infections. Numerous studies therefore focus on seroprevalence, i.e. the presence of SARS-CoV-2 antibodies, to determine and follow infection rates over an extended period of time [1, 2].

In Germany, several seroepidemiological studies were initiated after the onset of the pandemic, some of which focused on professions with infection-relevant contacts to examine the relationship between occupational contacts and infection risk. The SERODUS I and SERODUS II studies, which compared 2000 randomly selected residents with 700 employees and family members of fire crews and rescue service who were exposed to frequent occupational contacts with other people, revealed a seroprevalence of 3.1% for the general population and 4.4% for the high contact group, indicating an increased risk of SARS-CoV-2 infections for professions with high contact frequencies [3].

Similarly, a prospective longitudinal serological survey of healthcare workers of different organisational units in the first wave of the pandemic in a quaternary care hospital in Munich also showed that increased occupational contacts with COVID-19 patients correlate with a higher seroconversion rate (4.7% seroconversion for frontline health care workers compared to 0% for non-frontline workers) [4].

Also, a study from Columbia including 7045 workers showed that participants with multiple contacts to other people during their work shift had a higher seroprevalence for SARS-CoV-2 compared to the general population [5].

Infection risk in public transportation was studied in China, where a positive correlation between the risk of infection, co-travel time and seat location in relation to an index patient was found. This result was based on data from a total of 2334 index patients and 72 093 close contacts with co-travel times of 0–8 h from 19 December 2019 through 6 March 2020 [6].

In Germany in September 2020, the German railway carrier Deutsche Bahn Fernverkehr AG compared the number of PCR-confirmed SARS-CoV-2-positive cases between its employees of boarding service and non-boarding service. It was shown that boarding service staff had a slightly higher prevalence (0.31%) compared to other employees (0.26%), although the difference was within normal statistical fluctuations. Meanwhile, SARS-CoV-2 prevalence in the general population during the same period was 0.4% and thus comparable to that of the DB Fernverkehr personnel [7].

The motivation for this study was prompted by the wish to better understand the infection dynamics and risk for staff and indirectly for travellers in long distance trains in Germany. To this end, a longitudinal design with three time points for cross-sectional data collection and analysis was chosen so that not only time-dependent prevalence data could be collected but also actual incidence rates over defined periods could be assessed. The three test series took place in June 2020, October 2020 and February 2021. While for the first two test series, the B.1 variant was predominant in Germany, during the third test series, the alpha variant (B.1.1.7) was predominant and accounted for 67.2% of cases in Germany whereas beta (B.1.351) and delta (B.1.617.2) combined accounted for 3.2% [8].

## Methods

### Study design

In this multi-centre, prospective, longitudinal cohort study, the prevalence (acute prevalence and seroprevalence) and incidence of SARS-CoV-2 infections of three employee groups with varying frequencies of contact to passengers (train attendants, train drivers and maintenance workers) were tested. Nasopharyngeal swabs were taken for PCR testing and blood samples were taken for IgG antibody testing from all participating employees at study sites in Berlin, Frankfurt, Hamburg and Munich at three points in time (each called a ‘test series’) separated by approximately 4 months. In addition, demographical and epidemiological data of interest with regard to risk of infection were obtained from the employees.

### Study setting

Study sites were established in or near the central station in each respective city (in Berlin at the train station Ostbahnhof). In Hamburg, a second study site at one of the train maintenance sites was opened additionally in each test series.

### Sampling

The study sites were selected to cover different regions of Germany (Munich: southern part, Frankfurt: central western part, Berlin: northeastern part, Hamburg: northern part).

Employees were enrolled in a ratio of 8:3:3 (train attendants: train drivers: maintenance workers). At each test series, approximately 1100 employees were tested. The sample size of this longitudinal cohort study was justified by the expected precision of the estimation (width of a two-sided asymptotic confidence interval (CI): upper limit–lower limit) for the seroprevalence. A seroprevalence of 1% was assumed in each employee group. The resulting width of the CI (train attendants: 1.6%, train drivers and maintenance workers: 2.7%) were judged to be sufficient to gain preliminary insights in the infection status of employees of DB Fernverkehr.

For each employee group and location, a randomly sorted list of employees was created and the first employees were invited by letter sent to their private address. Each letter contained an individual code that allowed employees who were interested in participating in the study to register for a test appointment. Invited employees who either did not respond or explicitly declined the invitation were replaced by inviting employees next on the list. This approach assured the best feasible approximation to a random selection of employees per employee group and location, reflecting the actual demographics of the group.

In the second and third test series, participants from the previous test series were re-invited with a higher priority to obtain the necessary sample size for the longitudinal study design. Contact data were acquired again and merged by company-wide unique personnel number to (i) react to potentially changed postal addresses and (ii) exclude employees who lost their eligibility (e.g. due to retirement or a change in eligibility). Vacant slots were filled by inviting employees as per the randomised procedure described above. As a result, in the second and third test series, participants consisted of employees who already took part in the previous test series and of those who were newly recruited. No formal test was performed to compare the results of these subgroups.

### Data collection and procedures

After obtaining informed consent at each test series, nasopharyngeal swabs were taken to detect acute SARS-CoV-2 infections by PCR (RIDA^®^ GENE SARS-CoV-2 test with the analysis performed on the Roche LightCycler^®^ 480II), whereas blood samples were taken to test for SARS-CoV-2-specifig IgG antibodies (Anti-SARS-CoV-2 ELISA (IgG) test by EUROIMMUN AG). The IgG test recognises the S1 domain of the spike protein including the receptor-binding domain, which was used as evidence of a past infection. According to the manufacturer EUROIMMUN AG (Lübeck, Germany), the sensitivity of the antibody test is 94.4% for samples obtained 10 days after symptom onset or after verification of a positive infection, while the specificity is 99.6% [9]. The ratio between the extinction of the sample and the extinction of the calibrator was used to determine the serological status, whereby a ratio smaller than 0.8 was considered negative, a ratio between 0.8 and smaller than 1.1 borderline and a ratio of 1.1 or above positive [9]. Borderline cases were reported as such. Sensitivity and specificity of the PCR assay were assumed to be 100%. In addition to these tests, questionnaires were used to collect information on demographical and epidemiological factors that may increase the susceptibility to SARS-CoV-2 infections. These included the following age, sex, chronic lung diseases, diseases of the immune system, cardiovascular diseases, diabetes mellitus, cigarette consumption, number of persons per household, number of children per household, influenza vaccination, predisposition to common cold, working hours, frequency of wearing face masks, previous results from SARS-CoV-2 tests performed outside this study, average number of contacts to colleagues per week lasting more than 15 min, average number of private contacts per week lasting more than 15 min, current symptoms (fever, muscle pain, sore throat, coughing, rhinitis, chest pain, headache, diarrhoea, anosmia and ageusia), past symptoms and duration of those symptoms.

The company's physician immediately informed participants who were tested positive by PCR. IgG-positive participants were also informed about their test result. The pseudonymised subject code was only broken in cases of positive test results.

### Data analysis

Participants with missing or borderline test results were excluded from the analysis. A sensitivity analysis was conducted, using the borderline test results as positive test results.

Acute prevalence (PCR testing) and seroprevalence (SARS-CoV-2 antibodies) were calculated as the apparent prevalence (ratio of positive test results to non-missing test results per test series).

The cumulative incidence was defined as the number of employees with at least one positive test result (PCR or antibody test) since their enrolment into the study. Thus, in case a subject had been tested positive, the subject was counted positive for all subsequent visits.

Incidence was defined as the number of employees who had been tested negative (both PCR and antibody test) in the previous test series but positive (either PCR or antibody test) in the subsequent test series. Additionally, 95% Clopper–Pearson CI were calculated for each employee group and overall. Possible differences between the employee groups were tested by using a *χ*^2^ test and descriptive *P*-values were provided. Employees who did not return for subsequent testing (drop-outs) were replaced. Subgroups were defined by the first enrolment to the study (first test series, second test series and third test series) to investigate the impact of replacing the lost to follow-up employees. Additionally, *χ*^2^ tests were applied to analyse potential associations (potential risk factors) of all demographical and epidemiological characteristics (irrespective of the employee group) on the cumulative incidence. Analyses were described in a statistical analysis plan, which was finalised prior to the analysis and performed by using the statistical software SAS^®^ version 9.4.

## Results

**Fig. 1.**
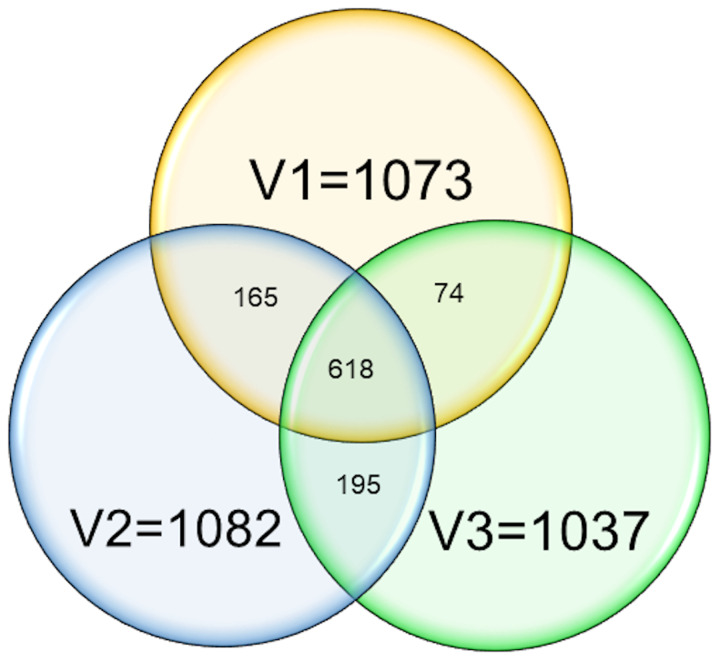
Number of participants in the first (visit 1, V1), second (visit 2, V2) and third (visit 3, V3) test series. Numbers in overlapping circles refer to number of employees participating in the respective test series. In total, 618 employees participated in all three test series.

The first test series took place from 29 June 2020 to 3 July 2020 with 1073 participants, the second one from 26 October 2020 to 30 October 2020 with 1082 participants, and the third and final one from 24 February 2021 to 2 March 2021 with 1037 participants (Fig. 1). During the study, new participants had to be recruited for the second and third test series to compensate for drop-outs. Among the 1037 participants of the third and final test series, 692 had already been enrolled in the first test series and 813 in the second test series. In the three test series, statistical differences were observed among the three employee groups when comparing their basic demographic characteristics and possible infection risk factors. For example, in the third test series, there were overall more male than female (69% *vs.* 31%), the average age was 45 years, the prevalence of cardiovascular disease was 9.7% and of diabetes was 3.6%, and 28% have smoked in the past 12 months (Table 1; detailed information on demographic characteristics and risk factors for the third test series is provided in Supplementary 01, Tables S9, S17 and S18–20, respectively, and for all test series in Supplementary 01, Tables S2, S14, S15 and S38–41, respectively).

**Table 1.** Selection of relevant demographic characteristics of DB employees in the third test series

Selected demographics	Employee groups			Total number of employees	*P*-value
	Train attendants	Train drivers	Maintenance workers		
Male^a^	282 (47.9%)	224 (97.4%)	204 (95.3%)	710 (68.7%)	<0.0001
Median age^a^ [IQR]	45 [36;51]	50 [39;56]	48 [36;55]	47 [37;53]	<0.0001
Cardiovascular disease^b^	40 (6.9%)	25 (11.1%)	34 (16.2%)	99 (9.7%)	0.0004
Diabetes^b^	16 (2.7%)	9 (4.0%)	12 (5.7%)	37 (3.6%)	0.1347
Non-smokers^b^	378 (65.3%)	195 (86.3%)	160 (75.8%)	733 (72.1%)	<0.0001
Household without children^b^	403 (69.0%)	172 (76.1%)	146 (68.9%)	721 (70.5%)	0.3069
Contact with 4 to >8 colleagues more than 15 min/week^c^	529 (91.2%)	140 (61.4%)	190 (87.6%)	859 (83.8%)	<0.0001

a See Supplementary 01, Table 2; IQR, interquartile range.

b See Supplementary 01, Table 17.

c See Supplementary 01, Table 18.

The prevalence of cardiovascular diseases differed between the employee groups. Maintenance workers tended to have the highest prevalence (16.2%), followed by train drivers (11.1%) and train attendants (6.9%). Furthermore, train drivers had the highest rate of non-smokers (86.3%) as compared to maintenance workers (75.8%) and train attendants (65.3%) (Supplementary 01, Table S17). Complete data on baseline characteristics are compiled in Supplementary material 01 (Tables S10–17).

### Prevalence of acute infection

Data on acute infections are summarised in Table 2. At the first test series, out of 1068 tested employees, one maintenance worker was tested positive by PCR. At the time of swabbing, this maintenance worker did not show any symptoms indicative of COVID-19. At the same time, he was also one of the 20 employees who had a positive antibody result. Based on these data, the overall acute prevalence across the three employee groups was 0.1% (95% CI 0.0–0.5). Stratified by employee groups, it was 0.0% (95% CI 0.0–0.6) for train attendants, 0.0% (95% CI 0.0–1.5) for train drivers and 0.5% (95% CI 0.0–2.7) for maintenance workers (*P* = 0.1155) (Supplementary 03, Table A).

**Table 2.** PCR test results (acute prevalence)

PCR test results	Test series	Employee groups			Total number of employees	*P*-Value^a^
		Train attendants	Train drivers	Maintenance workers		
Negative	1	625 (100.0 %)	242 (100.0 %)	200 (99.5 %)	1067 (99.9 %)	
	2	612 (99.5%)	235 (99.6%)	226 (99.6%)	1073 (99.5%)	
	3	586 (99.7%)	230 (100.0%)	216 (99.5%)	1032 (99.7%)	
Positive	1	**0** (**0.0 %)**	**0** (**0.0 %)**	**1** (**0.5%)**	**1** (**0.1%)**	0.1155
	2	**3** (**0.5%)**	**1** (**0.4%)**	**1** (**0.4%)**	**5** (**0.5%)**	0.9908
	3	**2** (**0.3%)**	**0** (**0.0%)**	**1** (**0.5%)**	**3** (**0.3%)**	0.2198

a P-values between occupational groups are best on a Chi-squared test.

At the second test series, out of 1078 tested employees, three train attendants, one train driver and one maintenance worker were tested positive by PCR. One of the three train attendants tested positive was aware of his infection and thus had been isolated. This employee was also tested antibody-positive. Unlike the other four employees who were tested positive and were asymptomatic, he also reported symptoms. Based on these data, the overall acute prevalence across the three employee groups was 0.5% (95% CI 0.2–1.2). Stratified by employee groups, it was 0.5% (95% CI 0.0–1.4) for train attendants, 0.4% (95% CI 0.0–2.3) for train drivers and 0.4% (95% CI 0.0–2.4) for maintenance workers (*P* = 0.9908) (Supplementary 03, Table B).

At the third test series, out of 1035 tested employees, two train attendants and one maintenance worker had a positive PCR test. Two of the three PCR-positive employees (both train attendants) were aware of their infection before the time of testing, because they already had a positive PCR test result, which was performed outside this study several weeks before the third test series and had already completed the isolation period. These two employees also had positive antibody findings. The PCR-positive maintenance worker, on the other hand, reported having had mild symptoms of common cold a few days before the testing. At the time of testing, this employee was asymptomatic. The antibody result was negative, probably due to the short time period between the infection and the testing. The overall acute prevalence across the three employee groups was 0.3% (95% CI 0.1–0.8). Stratified by employee groups, it was 0.3% (95% CI 0.0–1.2) for train attendants, 0.0% (95% CI 0.0–1.6) for train drivers and 0.5% (95% CI 0.0–2.5) for maintenance workers (*P* = 0.6252) (Supplementary 03, Table C).

### Seroprevalence

Seroprevalence data are summarised in Table 3. At the first test series, 20 out of 1064 participating employees were tested positive, including eight train attendants, six train drivers and six maintenance workers. Thus, the overall seroprevalence was 1.9% (95% CI 1.2–2.9) across the three employee groups. Stratified by employee groups, seroprevalence was 1.3% (95% CI 0.6–2.5) for train attendants, 2.5% (95% CI 0.9–5.4) for train drivers and 3.0% (95% CI 1.1–6.4) for maintenance workers (*P* = 0.2198).

**Table 3.** Antibody test results (seroprevalence)

Antibody test results	Test series	Employee groups			Total number of employees	*P*-Value^a^
		Train attendants	Train drivers	Maintenance workers		
Negative	1.	615 (98.7%)	234 (97.5%)	195 (97.0%)	1044 (98.1%)	
	2.	597 (97.4%)	232 (98.3%)	221 (97.4%)	1050 (97.6%)	
	3.	536 (93.2%)	219 (96.1%)	193 (91.5%)	948 (93.5%)	
Positive	1.	**8** (**1.3 %)**	**6** (**2.5 %)**	**6** (**3.0%)**	**20** (**1.9%)**	0.2198
	2.	**16** (**2.6%)**	**4** (**1.7%)**	**6** (**2.6%)**	**26** (**2.4%)**	0.7160
	3.	**39** (**6.8%)**	**9** (**3.9%)**	**18** (**8.5%)**	**66** (**6.5%)**	0.1390

a P-values between occupational groups are based on a Chi-squared test.

At the second test series, 26 out of 1076 employees were tested positive, including 16 train attendants, four train drivers and six maintenance workers. Thus, the overall seroprevalence was 2.4% (95% CI 1.6–3.5) across the three employee groups. Stratified by employee groups, seroprevalence was 2.6% (95% CI 1.5–4.2) for train attendants, 1.7% (95% CI 0.5–4.3) for train drivers and 2.6% (95% CI 1.0–5.7) for maintenance workers (*P* = 0.7160) (Supplementary 03, Table B).

At the third test series, out of 1014 tested employees, 66 were tested positive, including 39 train attendants, nine train drivers and 18 maintenance workers. Thus, the overall seroprevalence was 6.5% (95% CI 5.1–8.2) across the three employee groups. Stratified by employee groups, seroprevalence was 6.8% (95% CI 4.9–9.2) for train attendants, 4.0% (95% CI 1.8–7.4) for train drivers and 8.5% (95% CI 5.1–13.1) for maintenance workers (*P* = 0.1390).

The overall portion of seropositive participants who reported to not have had symptoms since March 2020 ranged from 20.3% (third test series) to 38.5% (second test series) (Supplementary 03, Table C).

### Cumulative incidence

Out of 944 participants with non-missing values in the third test series for the calculation of the cumulative incidence over the entire study, 80 were tested either PCR- or antibody-positive at least once since enrolment into the study. These included 45 train attendants, 12 train drivers and 23 maintenance workers. Cumulative incidence (acute and past infections) after the third (final) test series in February 2021 was therefore 8.5% (95% interval CI 6.8–10.4): 8.5% (95% CI 6.2–11.2) for train attendants, 5.5% (95% CI 2.9–9.5) for train drivers and 11.8% (95% CI 7.6–17.2) for maintenance workers (Supplementary 03, Table C).

### Incidence

Incidence data are summarised in Table 4. Out of 756 participants who had already participated in the first test series and had been tested negative at that time, nine employees were tested positive by antibody or PCR test in the second test series. This corresponded to an incidence of 1.2% (95% CI 0.6–2.3) for the period between the first (29 June 2020 to 3 July 2020) and the second test series (26 October 2020 to 30 October 2020). Of these nine employees, five were train attendants, two were train drivers and two were maintenance workers resulting in an incidence of 1.2% (95% CI 0.4–2.7), 1.1% (95% CI 0.1–3.9) and 1.4% (95% CI 0.2–5.1), respectively (Supplementary 03, Table B).

**Table 4.** Incidence

Incidence	Test series	Employee groups			Total number of employees per group	*P*-value^a^
		Train attendants	Train drivers	Maintenance workers		
Negative	1–2	429 (98.8%)	181 (98.9%)	137 (98.6%)	747 (98.8%)	
	2–3	401 (94.8%)	187 (98.4%)	146 (91.3%)	734 (95.0%)	
Positive	1–2	**5** (**1.2%)**	**2** (**1.1%)**	**2** (**1.4%)**	**9** (**1.2%)**	0.9545
	2–3	**22** (**5.2%)**	**3** (**1.6%)**	**14** (**8.8%)**	**39** (**5.0%)**	0.0092

a P-values between occupational groups are based on a Chi-squared test.

Out of 773 participants who were tested negative during the second test series and attended the third test series, 39 employees were tested positive by antibody or PCR in the third test series. This corresponded to an incidence of 5.1% (95% CI 3.6–6.8) for the period between the second (26 October 2020 to 30 October 2020) and the third test series (24 February 2021 to 2 March 2021). Of these 39 employees, 22 were train attendants, three were train drivers and 14 were maintenance workers resulting in an incidence of 5.20% (95% CI 3.3–7.8), 1.6% (95% CI 0.3–4.5) and 8.8% (95% CI 4.9–14.3), respectively (Supplementary 03, Table C).

### Factors associated with infection risk

We have analysed factors that are commonly associated with infection risk in order to understand whether observed differences between the employee groups can be explained by differences in these associated risk factors. An association between past symptoms – especially fever (Supplementary 02, Figs S2 and S15), rhinitis (Supplementary 02, Figs S4 and S17), coughing (Supplementary 02, Figs S3 and S16), headache (Supplementary 02, Figs S5 and S18), anosmia and ageusia (Supplementary 02, Figs S7 and S20) – and cumulative incidence was found, which varied across test series. In the initial testing rounds, we found elevated infection rates in staff with diabetes and in households with children (see Supplementary 02, Figs S8, S10, S21 and S23, respectively). In general, private habits, e.g. meeting with more people during free time (Supplementary 02, Figs S12 and S25), and individual health status, such as cardiovascular disease (Table 1), had an influence on the infection risks but no finding was consistently observed throughout the three study rounds.

## Discussion

This longitudinal study compared the prevalence and incidence of SARS-CoV-2 infections between three employee groups of DB Fernverkehr AG (train attendants, train drivers and maintenance workers) to estimate the occupational risk of infection. The employee groups were selected based on their differing contact exposure with passengers and colleagues. Underlying the primary objective of obtaining seroprevalences from the different employee groups was the question on whether the different contact frequencies of the employee groups had an impact on the infection risk. The study also allowed to determine cumulative incidences over a period of almost one year. This study was explorative in nature and did not strive for a formal hypothesis testing on the differences in employee group infection risks. Throughout the three test series, acute prevalence of SARS-CoV-2 remained relatively low across all three employee groups ranging from 0.1% to 0.5%. The differences observed between employee groups were in all three test series not meaningful due to the low number of positive test results. A steady increase in seroprevalence was observed during the course of the study, reflecting the ongoing pandemic. While in the first test series it was 1.9%, it increased to 2.4% in the second test series and to 6.5% in the third test series. The observed differences among the employee groups were marginal. Maintenance workers exhibited the relatively highest seroprevalence throughout all three test series. The steady increase in seroprevalence in the course of the study was partly a consequence of the long half-life of SARS-CoV-2 IgG antibodies (according to a large US study with 452 antibody-positive healthcare workers, up to 92.4% remained still positive after 7–10 months [10]). In our study, which was run over 9 months, 66.7% of those tested positive in the first test series were still positive in the third test series, which includes the false-positive results in all rounds. However, to an even larger extent, the magnitude of the increase reflected the nationwide infection dynamics. The true infection rates appear to be a factor of 2.2–6.0 times higher than the cases officially reported to the authorities. This estimate is based on observed differences between the reported positive PCR results made public and the estimates of seroprevalence, which also captures undetected and unreported past infections, from a series of seroepidemiological studies initiated by the German federal government agency and research institute Robert Koch Institute (RKI) (‘CORONA-MONITORING local’ (CoMoLo)) [11–13], and the interim results of a Saarland-wide antibody study [14]. The same commercial laboratory test ‘Anti-SARS-CoV-2-ELISA (IgG)’ by EUROIMMUN (EUROIMMUN Medizinische Labordiagnostika AG, Lübeck, Germany) was used in this study of DB Fernverkehr AG as in the studies mentioned above [11–14]. The data may thus be comparable with the following caveats. In this study, borderline cases were neither regarded as negative nor positive but as missing. However, as part of a sensitivity analysis (see Supplementary 03, Tables A–C), the data were also calculated as if borderline results were positive, which did not change the relative frequencies of prevalence or incidence. Furthermore, the RKI studies did only count neutralizing antibodies [11–14], whereas in this study, non-neutralizing antibodies were included in the determination of seroprevalence, thereby implying a possible underestimation of seroprevalence in those studies when compared with our study. The observed increase of the cumulative prevalence at DB employees corresponds well to the nationwide pandemic dynamics. Employing the factor 2.2–6 to account for the underreported and undetected cases, a Germany-wide cumulative incidence of 0.6–1.4%, 1.9–4.4% and 6.5–17.7% can be calculated for the points in time of the first, second and third test series, respectively [15]. Thus, the seroprevalence rates of 1.9%, 2.4% and 6.5% for the first, second and third test series, in our study, were compatible to the overall infection dynamics in Germany, if not even lower. Likewise, the estimated nationwide incidence was 1.2–2.8% between the time periods of the first and second test series and 4.8–13.2% between those of the second and third series, respectively [15], corresponding to the observed incidence of 1.2% and 5.0% for the same time periods in our study.

Several limitations regarding the interpretation of this study apply. First, the study design was exploratory in nature. Therefore, the results do not allow conclusions based on formal hypothesis testing. Consequently, the study results do not allow for drawing the conclusion whether more exposure to occupational contact poses a higher risk of infection or not. Furthermore, due to the small number of observed infections and the limited sample size, potential confounders (e.g. age, lifestyle and living situation either alone or with family members) could not be considered in the analysis.

Second, the study did not account for a possible underestimation of the prevalence of acute infections at each test series. This bias would be introduced by employees not attending study visits because of acute symptomatic infections or being quarantined.

Third, it should be noted that although hygiene rules such as wearing masks applied in general in the same way for all three employee groups, the degree of social contact during work was different among these groups. Masks were also mandatory for passengers during their journey [16]. Train attendants had frequently but mainly temporary contact to passengers while train drivers and maintenance workers had only occasional contact to passengers during commutes. Maintenance workers needed to work in teams, which made keeping enough social distance often more difficult due to tight workspaces and as a result, this might have contributed to a higher rate of infection in this employee group.

Fourth, the estimation of the nationwide infection rate is also subject to several caveats. For example, the testing strategy in Germany underwent a change, which initially was more restricted due to limited capacity than later in the pandemic, possibly leading to an over- or underestimation of the true number of infections in Germany at the time of the test series in this study. In addition, no statistical test was performed to identify any differences in population characteristics between the employees of Deutsche Bahn Fernverkehr and the general population, such as age and gender distribution limiting the extrapolation of the results to the general population.

Fifth, the study does not allow direct conclusions for the infection risks of passengers, since this was not examined. However, since train attendants and passengers share the same compartment, it can be assumed that the infection risk for passengers emanating from train attendants is modest as well.

Also, it is noted that detection of reinfections is limited by the chosen study design and definition of incidence. A person tested positive in two consecutive test series would only have been counted once.

In summary, the results of this study did not show any evidence that train attendants with the highest degree of contacts to passengers and colleagues had noticeably higher rates of SARS-CoV-2 infections than train drivers and maintenance workers. Maintenance workers exhibited the highest seroprevalence followed by train attendants and train drivers. The overall infection rates within the tested employees of the DB Fernverkehr were also in accordance with the nationwide infection dynamics. However, in general, no conclusive differences between the employee groups were observed either in acute prevalence or seroprevalence throughout the study. Nonetheless, the incidence between the second and the third test series showed a marked difference between the employee groups. The maintenance workers markedly showed the highest incidence, followed by train attendants and train drivers.

## Data Availability

The data that support the findings of this study are available upon request by contacting the corresponding author by email (Robert.Schultz-Heienbrok@charite-research.org). The data referenced in the text are compiled in Supplements. Supplementary 01 comprises the demographic and epidemiologic baseline characteristics. Supplementary 02 comprises a figure on enrolment and figures on underlying risk factors. Supplementary 03 comprises the sensitivity analyses.

## Supplementary material

For supplementary material accompanying this paper visit https://doi.org/10.1017/S095026882200070X. click here to view supplementary material
