# Supplementary material for: Longitudinal study of SARS-CoV-2 infections in different employee groups of long distance train services from June 2020 until February 2021 in Germany
Source: Epidemiol Infect. 2022 Apr 20;150:e88. doi: 10.1017/S095026882200070X (PMC9095852; doi:10.1017/S095026882200070X)
Supplement: Supplementary file 1 [file hygsup.zip › S095026882200070Xsup001.pdf]

03 Supplementary Material

Longitudinal study of SARS-CoV-2 infections in different employee groups of long distance train services in Germany

Authors:

HyoungJin Kim[1,5] , Robert Schultz-Heienbrok[1*], Markus Uhle[1], Jenni Neubert[1], Fabian Ball[2], Matthes Metz[3], Christian Gravert[4]

[1]Charité Research Organisation GmbH, Germany, [2]DB Fernverkehr AG, Germany, [3]Department of Biostatistics, GCP-Service International Ltd. & Co. KG, Germany,

[4]Deutsche Bahn AG, Germany, [5]Janssen-Cilag GmbH, Germany

Author for correspondence:

Robert Schultz-Heienbrok E-Mail: robert.schultz-heienbrok@charite-research.org

Table A: Primary objectives in 1st test series

	Train attendants		Train drivers		Maintenance workers		Total		p-value*
**IgG test**									0.0963
Negative	615	(98.7)	234	(97.1)	195	(96.5)	1044	(97.9)	
Positive	8	(1.3)	7	(2.9)	7	(3.5)	22	(2.1)	
Missing	2	(0.3)	1	(0.4)	1	(0.5)	4	(0.4)	
**PCR test**									0.1155
Negative	625	(100.0)	242	(100.0)	200	(99.5)	1067	(99.9)	
Positive	0	(0.0)	0	(0.0)	1	(0.5)	1	(0.1)	
Missing	0	(0.0)	0	(0.0)	2	(1.0)	2	(0.2)	
**Infection rate**									0.0947
Negative	615	(98.7)	234	(97.1)	194	(96.5)	1043	(97.9)	
Positive	8	(1.3)	7	(2.9)	7	(3.5)	22	(2.1)	
Missing	2	(0.3)	1	(0.4)	2	(1.0)	5	(0.5)	
**Infection rate with additional tests**									0.2197
Negative	613	(98.4)	234	(97.1)	194	(96.5)	1041	(97.7)	
Positive	10	(1.6)	7	(2.9)	7	(3.5)	24	(2.3)	
Missing	2	(0.3)	1	(0.4)	2	(1.0)	5	(0.5)	
**Prevalence (%)**									
**Not adjusted**									
IgG test (Estimator + 95%-CI)	1.28	(0.40, 2.17)	2.90	(0.78, 5.02)	3.47	(0.94, 5.99)	2.06	(1.21, 2.92)	
IgG test (Estimator + 90%-CI)	1.28	(0.54, 2.03)	2.90	(1.13, 4.68)	3.47	(1.35, 5.58)	2.06	(1.35, 2.78)	
PCR test (Estimator + 95%-CI)	0.00	(0.00, 0.59)	0.00	(0.00, 1.51)	0.50	(0.01, 2.74)	0.09	(0.00, 0.52)	
PCR test (Estimator + 90%-CI)	0.00	(0.00, 0.48)	0.00	(0.00, 1.23)	0.50	(0.03, 2.34)	0.09	(0.00, 0.44)	
Infection rate(Estimator + 95%-CI)	1.28	(0.40, 2.17)	2.90	(0.78, 5.02)	3.48	(0.95, 6.02)	2.07	(1.21, 2.92)	
Infection rate(Estimator + 90%-CI)	1.28	(0.54, 2.03)	2.90	(1.13, 4.68)	3.48	(1.36, 5.61)	2.07	(1.35, 2.78)	
**Adjusted**									
IgG test (Rogan-Gladen Estimator)	0.94		2.66		3.26		1.77		
PCR test (Rogan-Gladen Estimator)	0.00		0.00		0.50		0.09		
Infection rate (Regression + 95%-CI)	1.21	(0.50, 2.88)	2.61	(0.87, 7.55)	2.55	(0.78, 8.00)	-		

For two participants (one train driver and one maintenance worker), the IgG test result was judged as missing due to a borderline result.
n: Number of non-missing observations; %: Percentages for response categories based on total number of non-missing observations in the respective group, Percentages for missing observations are based on number of all subjects in the respective group; CI: confidence interval
*: Chi-squared test

Table B: Primary objectives in 2nd test series

	Train attendants		Train drivers		Maintenance workers		Total		p-value*
**IgG test (n (%))**									0.4704
Negative	597	(96.8)	232	(98.3)	221	(96.9)	1050	(97.1)	
Positive	20	(3.2)	4	(1.7)	7	(3.1)	31	(2.9)	
Missing	1	(0.2)	0	(0.0)	0	(0.0)	1	(0.1)	
**PCR test (n (%))**									0.9908
Negative	612	(99.5)	235	(99.6)	226	(99.6)	1073	(99.5)	
Positive	3	(0.5)	1	(0.4)	1	(0.4)	5	(0.5)	
Missing	3	(0.5)	0	(0.0)	1	(0.4)	4	(0.4)	
**Incidence (n (%))**									0.8882
Negative	429	(98.4)	181	(98.9)	137	(98.6)	747	(98.5)	
Positive	7	(1.6)	2	(1.1)	2	(1.4)	11	(1.5)	
Missing	3	(0.7)	0	(0.0)	0	(0.0)	3	(0.4)	
**Infection rate (n (%))**									0.7931
Negative	589	(96.1)	228	(97.0)	218	(96.0)	1035	(96.3)	
Positive	24	(3.9)	7	(3.0)	9	(4.0)	40	(3.7)	
Missing	5	(0.8)	1	(0.4)	1	(0.4)	7	(0.7)	
**Infection rate with additional tests (n (%))**									0.7116
Negative	589	(96.1)	228	(97.0)	217	(95.6)	1034	(96.2)	
Positive	24	(3.9)	7	(3.0)	10	(4.4)	41	(3.8)	
Missing	5	(0.8)	1	(0.4)	1	(0.4)	7	(0.7)	
**Incidence (%)**									
Incidence (Estimator + 95%-CI)	1.61	(0.43, 2.79)	1.09	(0.00, 2.60)	1.44	(0.00, 3.42)	1.45	(0.60, 2.30)	
Incidence (Estimator + 90%-CI)	1.61	(0.62, 2.60)	1.09	(0.00, 2.36)	1.44	(0.00, 3.10)	1.45	(0.74, 2.17)	
**Prevalence (%)**									
Not adjusted									
IgG test (Estimator + 95%-CI)	3.24	(1.84, 4.64)	1.69	(0.05, 3.34)	3.07	(0.83, 5.31)	2.87	(1.87, 3.86)	
IgG test (Estimator + 90%-CI)	3.24	(2.07, 4.41)	1.69	(0.31, 3.08)	3.07	(1.19, 4.95)	2.87	(2.03, 3.70)	
PCR test (Estimator + 95%-CI)	0.49	(0.00, 1.04)	0.42	(0.00, 1.25)	0.44	(0.00, 1.30)	0.46	(0.06, 0.87)	
PCR test (Estimator + 90%-CI)	0.49	(0.03, 0.95)	0.42	(0.00, 1.12)	0.44	(0.00, 1.16)	0.46	(0.12, 0.80)	
Infection rate(Estimator + 95%-CI)	3.92	(2.38, 5.45)	2.98	(0.81, 5.15)	3.96	(1.43, 6.50)	3.72	(2.59, 4.85)	
Infection rate(Estimator + 90%-CI)	3.92	(2.63, 5.20)	2.98	(1.15, 4.80)	3.96	(1.83, 6.10)	3.72	(2.77, 4.67)	
**Adjusted**									
IgG test (Rogan-Gladen Estimator)	3.02		1.38		2.84		2.63		
PCR test (Rogan-Gladen Estimator)	0.49		0.42		0.44		0.46		
Infection rate(Regression + 95%-CI)	3.71	(2.46, 5.56)	3.04	(1.33, 6.78)	3.82	(1.82, 7.81)	-		

Only participants who had no missing values in both the first and second test series were included in the calculation of incidence.

n: Number of non-missing observations; %: Percentages for response categories based on total number of non-missing observations in the respective group, Percentages for missing observations are based on number of all subjects in the respective group; CI: confidence interval
*: Chi-squared test

Table C: Primary objectives in 3rd test series

	Train attendants		Train drivers		Maintenance workers		Total		p-value*
**IgG test (n (%))**									0.0411
Negative	536	(92.6)	219	(95.6)	193	(89.4)	948	(92.6)	
Positive	43	(7.4)	10	(4.4)	23	(10.6)	76	(7.4)	
Missing	10	(1.7)	1	(0.4)	2	(0.9)	13	(1.3)	
**PCR test (n (%))**									0.6252
Negative	586	(99.7)	230	(100.0)	216	(99.5)	1032	(99.7)	
Positive	2	(0.3)	0	(0.0)	1	(0.5)	3	(0.3)	
Missing	1	(0.2)	0	(0.0)	1	(0.5)	2	(0.2)	
**Incidence (n (%))**									0.0024
Negative	401	(94.8)	187	(98.4)	146	(90.1)	734	(94.7)	
Positive	22	(5.2)	3	(1.6)	16	(9.9)	41	(5.3)	
Missing	5	(1.2)	1	(0.5)	2	(1.2)	8	(1.0)	
**Infection rate (n (%))**									0.0263
Negative	487	(91.0)	205	(94.0)	172	(86.4)	864	(90.8)	
Positive	48	(9.0)	13	(6.0)	27	(13.6)	88	(9.2)	
Missing	54	(9.2)	12	(5.2)	19	(8.7)	85	(8.2)	
**Infection rate with additional tests (n (%))**									0.0236
Negative	481	(89.7)	204	(93.6)	170	(85.4)	855	(89.7)	
Positive	55	(10.3)	14	(6.4)	29	(14.6)	98	(10.3)	
Missing	53	(9.0)	12	(5.2)	19	(8.7)	84	(8.1)	
**Incidence (%)**									
Incidence (Estimator + 95%-CI)	5.20	(3.08, 7.32)	1.58	(0.00, 3.35)	9.88	(5.28, 14.47)	5.29	(3.71, 6.87)	
Incidence (Estimator + 90%-CI)	5.20	(3.43, 6.98)	1.58	(0.09, 3.07)	9.88	(6.02, 13.73)	5.29	(3.97, 6.61)	
**Prevalence (%)**									
**Not adjusted**									
IgG test (Estimator + 95%-CI)	7.43	(5.29, 9.56)	4.37	(1.72, 7.01)	10.65	(6.53, 14.76)	7.42	(5.82, 9.03)	
IgG test (Estimator + 90%-CI)	7.43	(5.63, 9.22)	4.37	(2.15, 6.59)	10.65	(7.20, 14.10)	7.42	(6.07, 8.77)	
PCR test (Estimator + 95%-CI[1])	0.34	(0.04, 1.22)	0.00	(0.00, 1.59)	0.46	(0.01, 2.54)	0.29	(0.06, 0.84)	
PCR test (Estimator + 90%-CI[1])	0.34	(0.06, 1.07)	0.00	(0.00, 1.29)	0.46	(0.02, 2.17)	0.29	(0.08, 0.75)	
Infection rate (Estimator + 95%-CI)	8.97	(6.55, 11.39)	5.96	(2.82, 9.11)	13.57	(8.81, 18.33)	9.24	(7.40, 11.08)	
Infection rate (Estimator + 90%-CI)	8.97	(6.94, 11.00)	5.96	(3.33, 8.60)	13.57	(9.57, 17.56)	9.24	(7.70, 10.79)	

**Adjusted**							
IgG test (Rogan-Gladen Estimator)	7.48		4.22		10.90		7.47
PCR test (Rogan-Gladen Estimator)	0.34		0.00		0.46		0.29
Infection rate (Regression + 95%-CI)	8.43	(6.33, 11.14)	4.85	(2.60, 8.87)	11.42	(7.29, 17.46)	-
Infection rate (Time-adjusted + 95%-CI)	0.19	(0.14, 0.26)	0.12	(0.07, 0.22)	0.29	(0.20, 0.42)	-

In Only participants who had no missing values in both the second and third test series were included in the calculation of incidence.as well as those participants tested negative in the second test series.
Infection rate: Participants who had a positive test result in either PCR or IgG test.
n: Number of non-missing observations; %: Percentages for response categories based on total number of non-missing observations in the respective group, Percentages for missing observations are based on number of all subjects in the respective group; CI: confidence interval (asymptomatic), [1]: Exact confidence interval due to employee groups without a positive test result.
*: Chi-squared test; The estimates of the regression are based on a logistic regression with age and gender as covariates; The time-adjusted estimator is based on a Poisson regression with age as covariate and working time as exposure time; The time-adjusted estimators indicate the infection rate per 8 h working day.
