# Supplementary material for: Longitudinal study of SARS-CoV-2 infections in different employee groups of long distance train services from June 2020 until February 2021 in Germany
Source: Epidemiol Infect. 2022 Apr 20;150:e88. doi: 10.1017/S095026882200070X (PMC9095852; doi:10.1017/S095026882200070X)
Supplement: Supplementary file 1 [file hygsup.zip › S095026882200070Xsup002.pdf]

02 Supplementary Material

Longitudinal study of SARS-CoV-2 infections in different employee groups of long distance train services in Germany

Authors:

HyoungJin Kim[1,5] , Robert Schultz-Heienbrok[1*], Markus Uhle[1], Jenni Neubert[1], Fabian Ball[2], Matthes Metz[3], Christian Gravert[4]

[1]Charité Research Organisation GmbH, Germany, [2]DB Fernverkehr AG, Germany, [3]Department of Biostatistics, GCP-Service International Ltd. & Co. KG, Germany, [4]Deutsche Bahn AG, Germany, [5]Janssen-Cilag GmbH, Germany

Author for correspondence:

Robert Schultz-Heienbrok
robert.schultz-heienbrok@charite-research.org

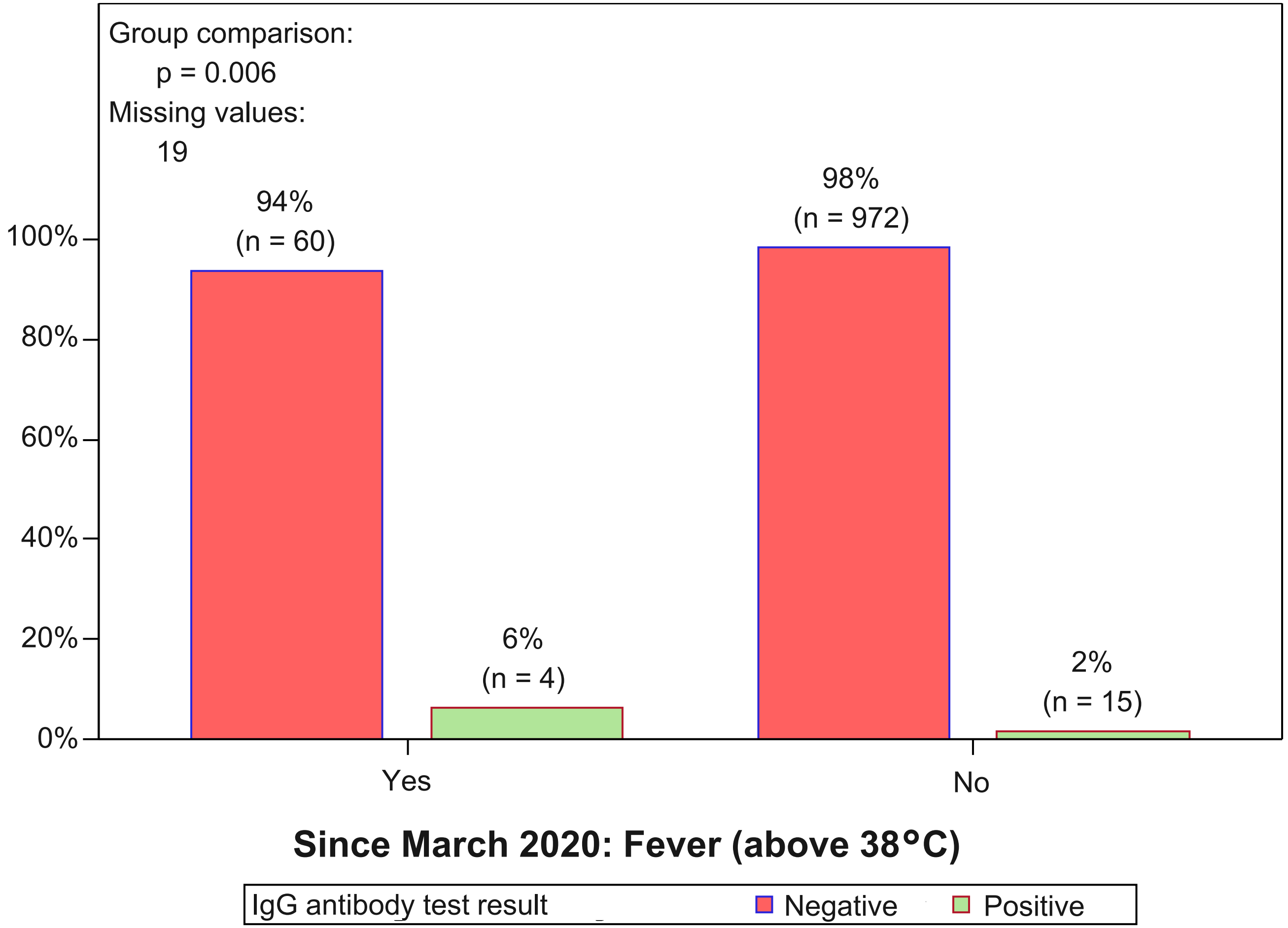

Figure 2: IgG test results vs. reported fever (since March 2020) in the first test series (June 2020). Results are based on the symptom reported by participants in the questionnaire during the first test series.

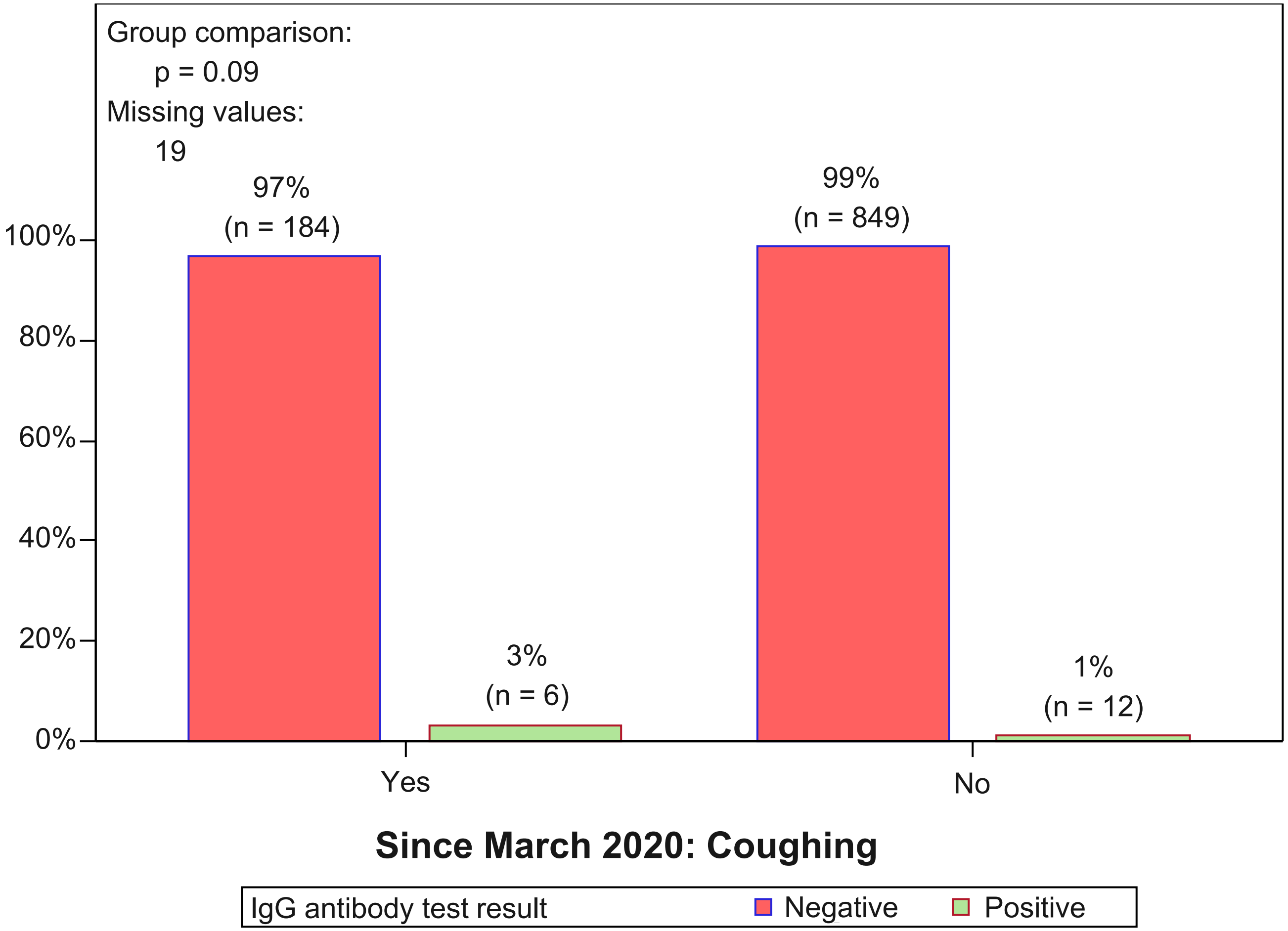

Figure 3: IgG test results vs. reported coughing (since March 2020) in the first test series (June 2020). Results are based on the symptom reported by participants in the questionnaire during the first test series.

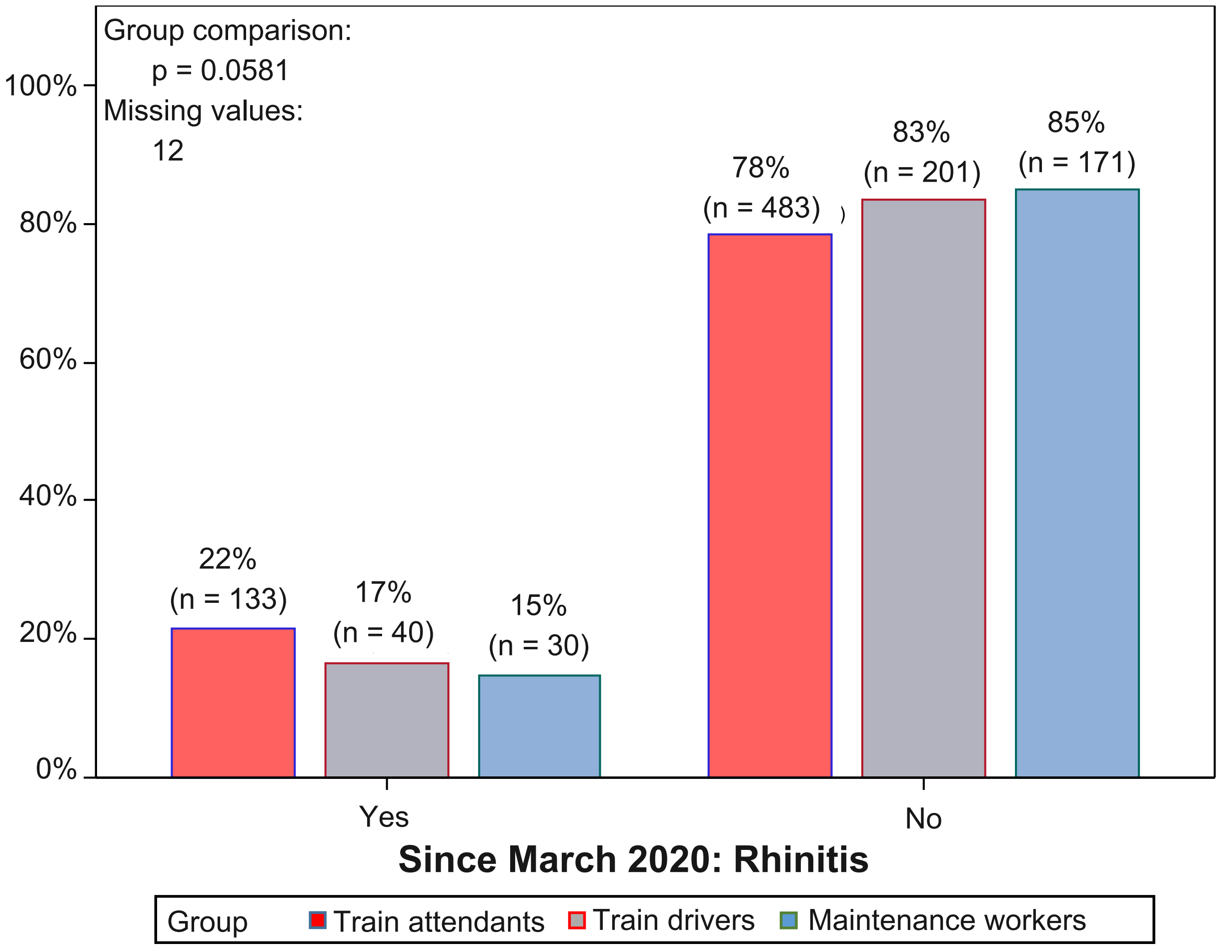

Figure 4: IgG test results vs. reported rhinitis (since March 2020) in first test series (June 2020). Results are based on the symptom reported by participants in the questionnaire during the first test series.

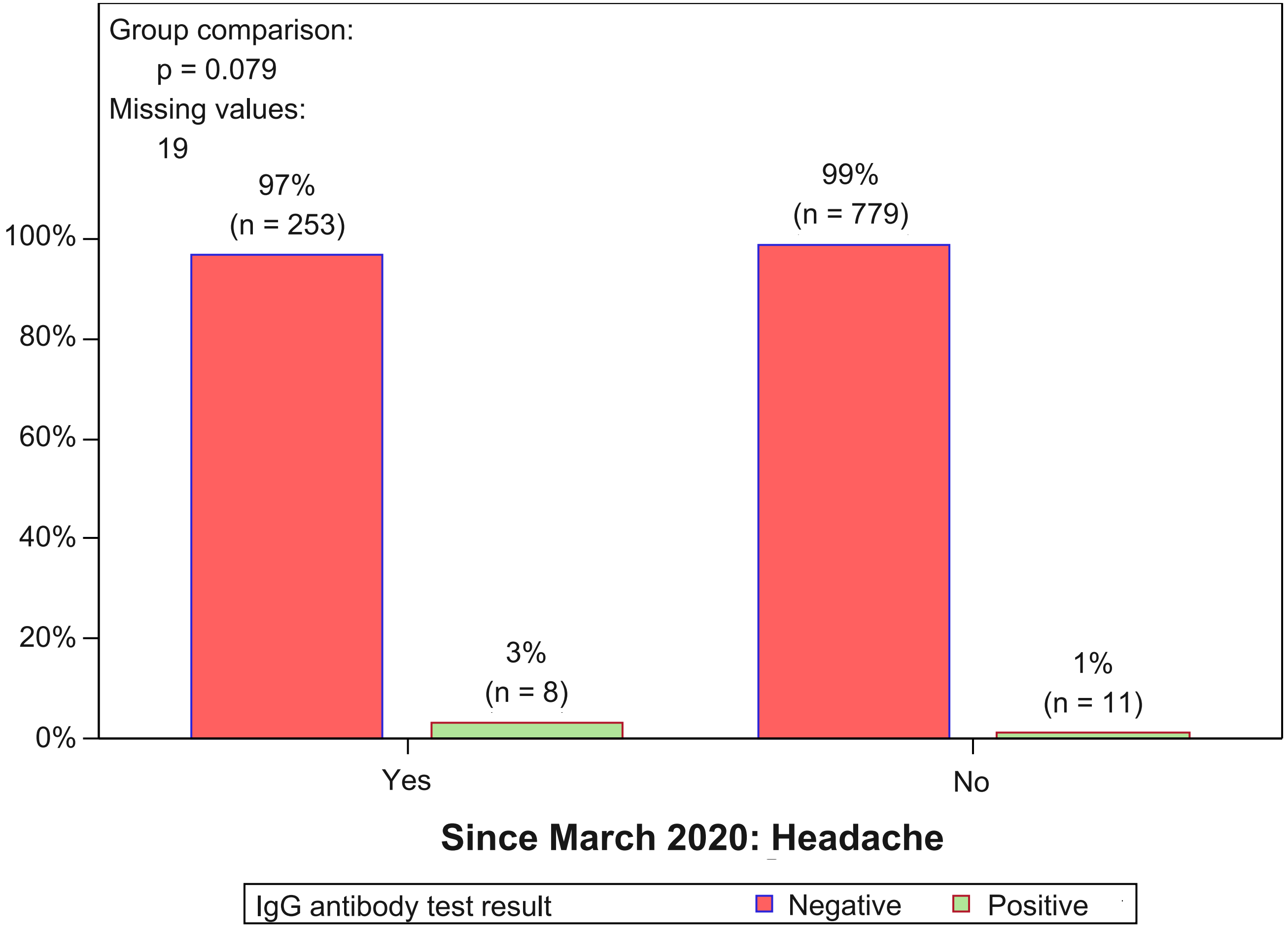

Figure 5: IgG test results vs. reported headache (since March 2020) in first test series (June 2020). Results are based on the symptom reported by participants in the questionnaire during the first test series.

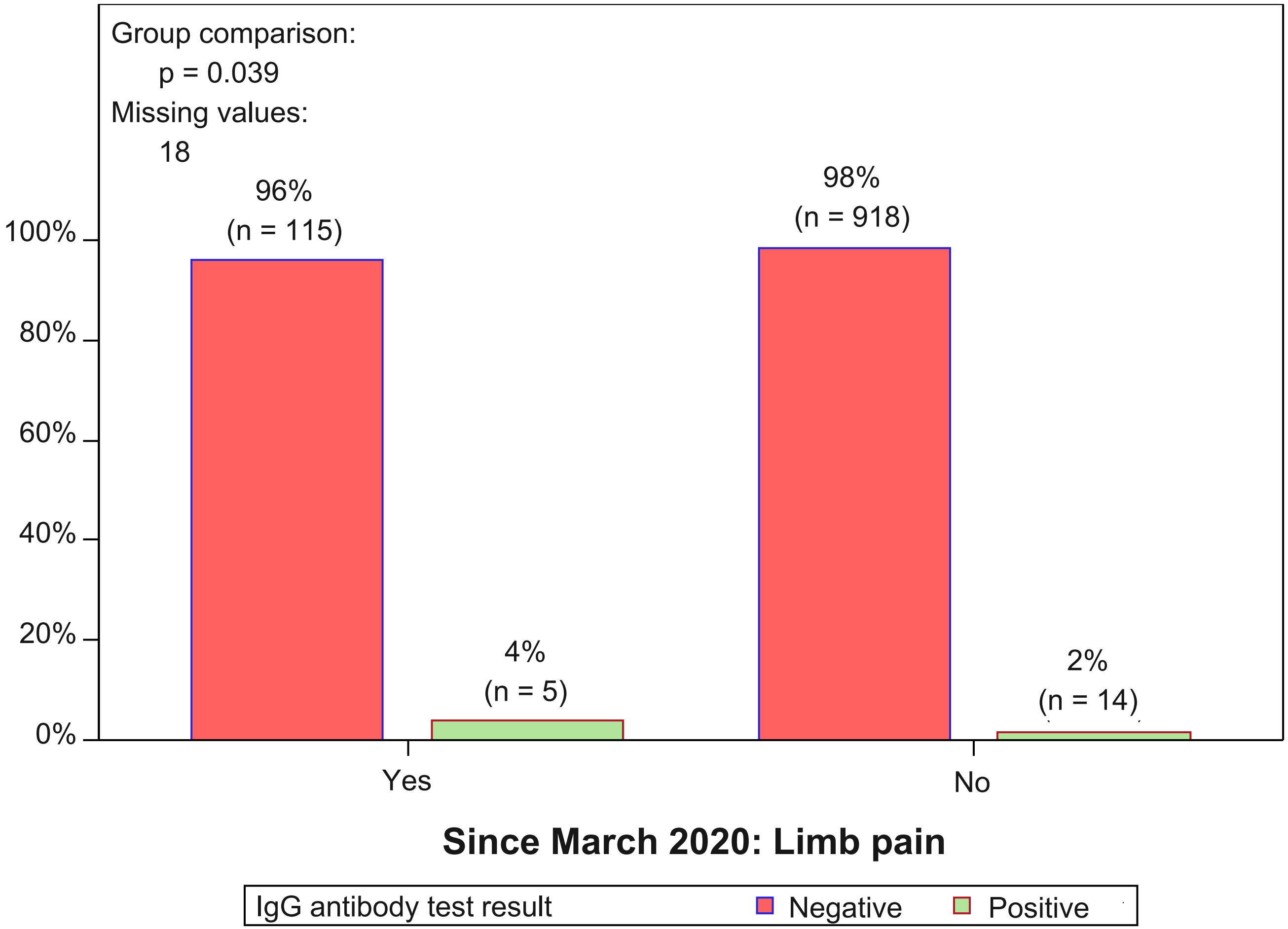

Figure 6: IgG test results vs. reported limb pain (since March 2020) in first test series (June 2020). Results are based on the symptom reported by participants in the questionnaire during the first test series.

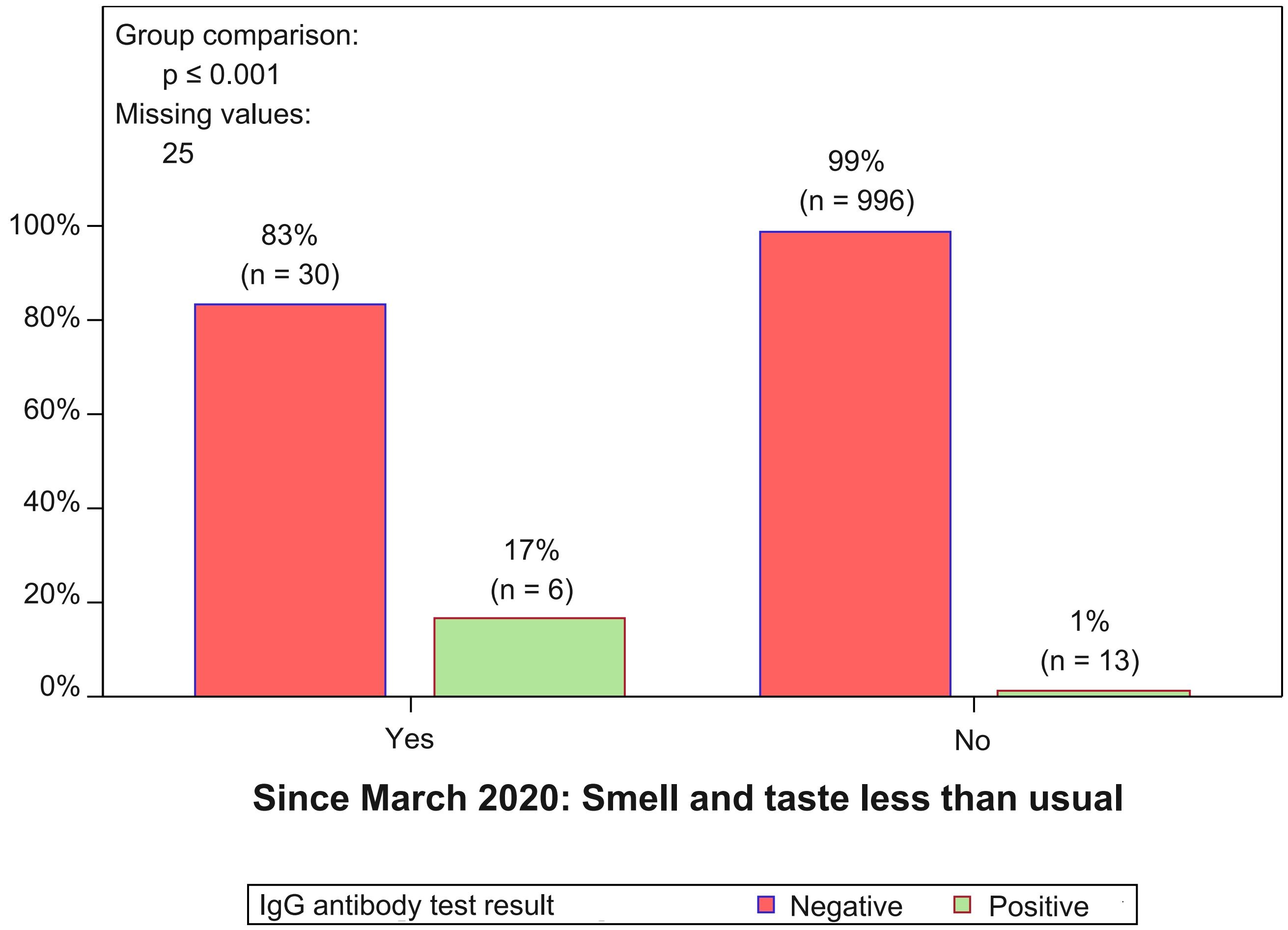

Figure 7: IgG test results vs. reported anosmia and ageusia (since March 2020) in first test series (June 2020). Results are based on symptoms reported by participants in the questionnaire during the first test series.

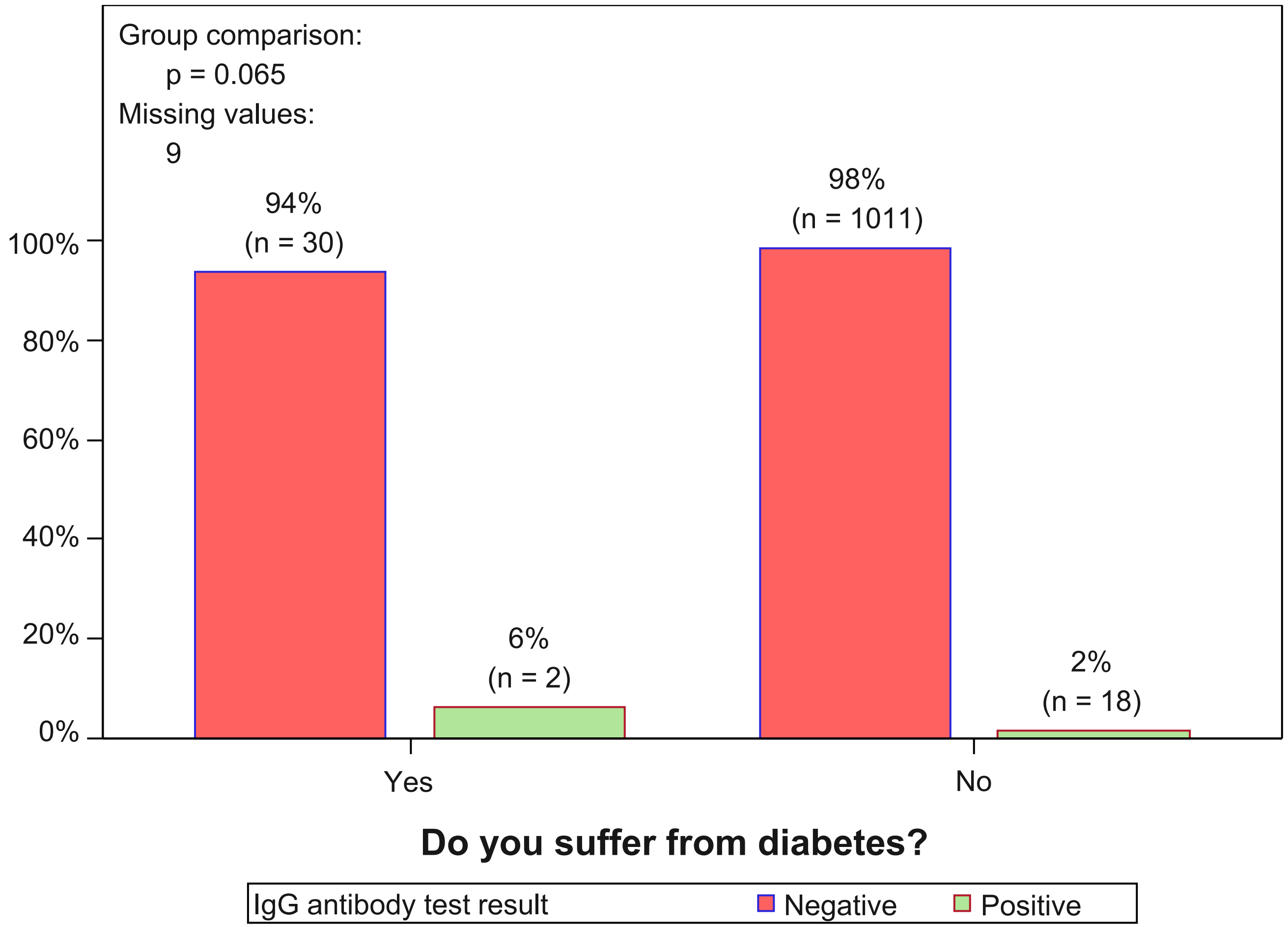

Figure 8: IgG test results vs. reported diabetes in first test series (June 2020). Results are based on the symptom reported by participants in the questionnaire during the first test series.

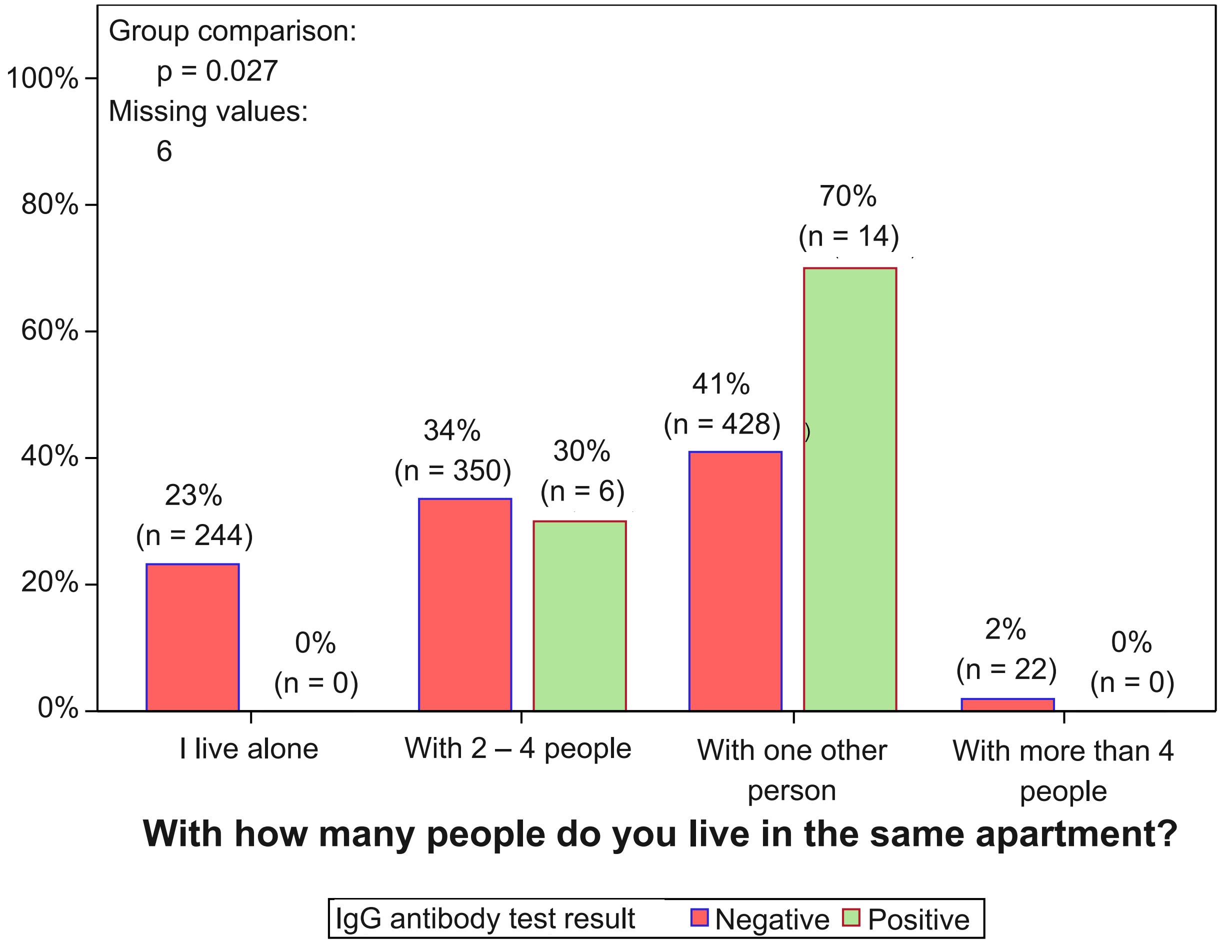

Figure 9: IgG test results vs. number of people living in same apartment (first test series: June 2020). Results are based on answers of participants in the questionnaire during the first test series.

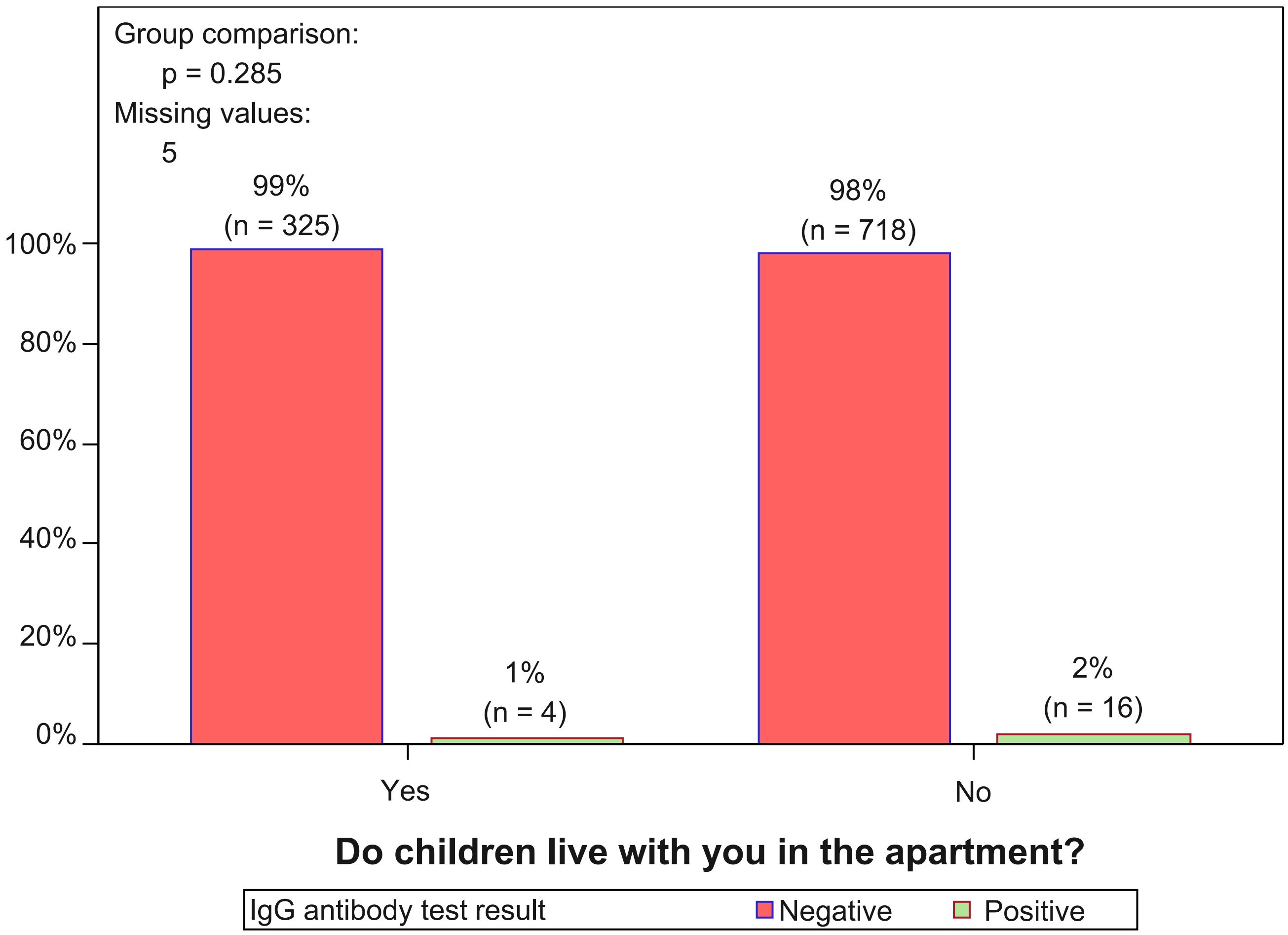

Figure 10: IgG test results vs. children in same apartment in the first test series (June 2020). Results are based on answers of participants in the questionnaire during the first test series.

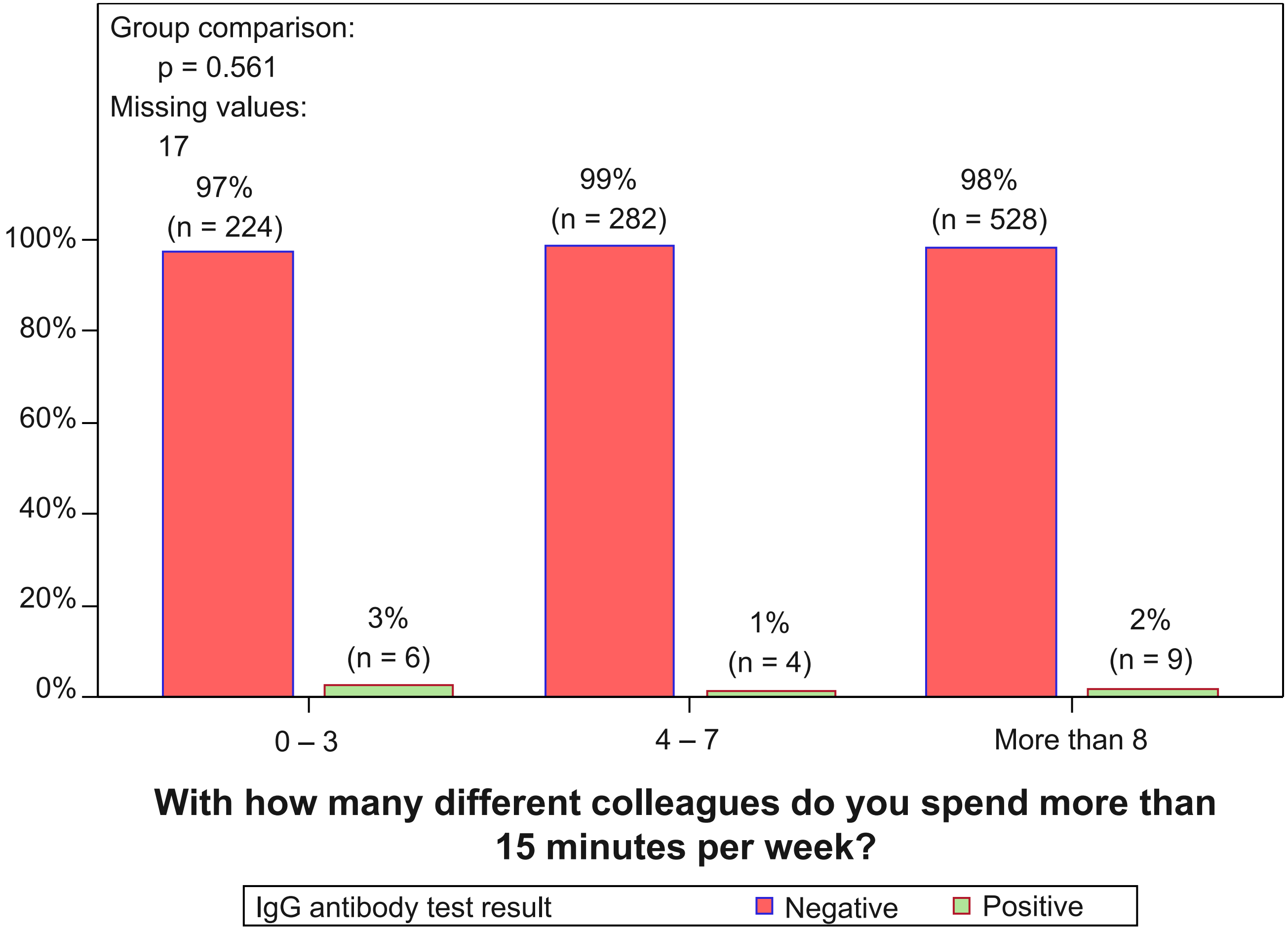

Figure 11: IgG test results vs. number of occupational contacts per week lasting more than 15 minutes (first test series: June 2020). Results are based on answers of participants in the questionnaire during the first test series.

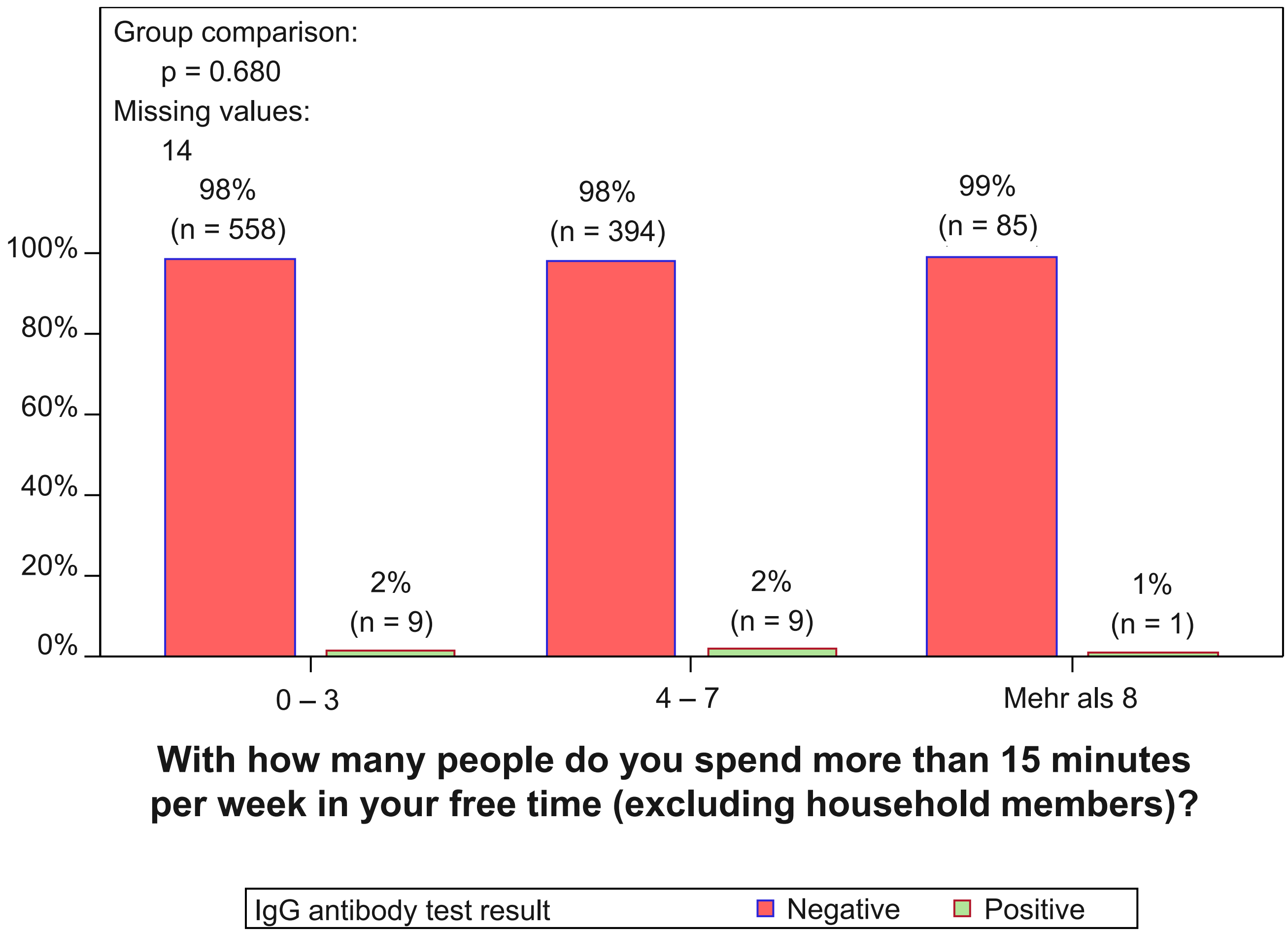

Figure 12: IgG test results vs. number of contacts to people during free time per week lasting more than 15 minutes (first test series: June 2020). Results are based on answers of participants in the questionnaire during the first test series.

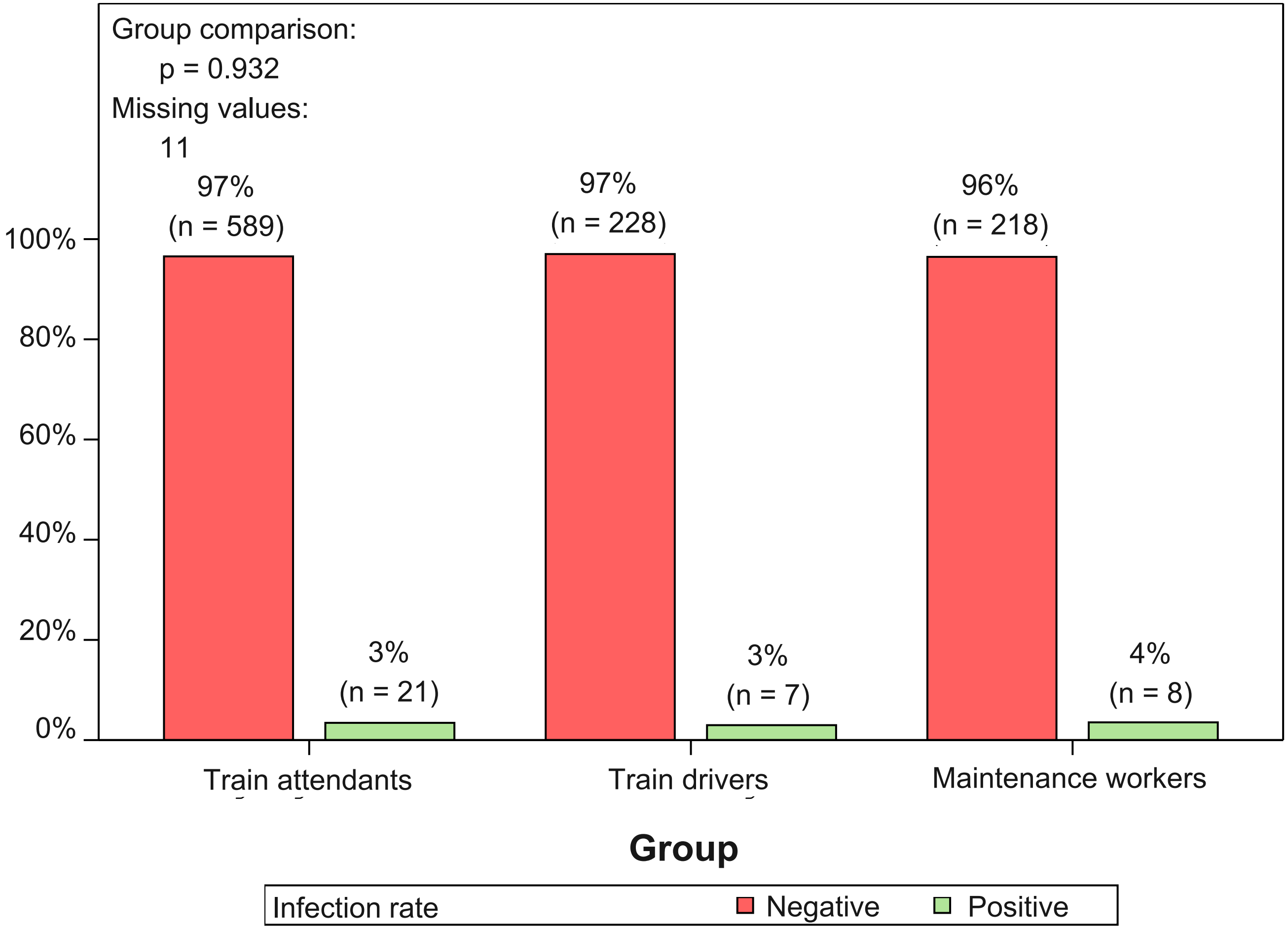

Figure 13: Comparison of employee groups vs. infection rate of participants in the second test series (October 2020).

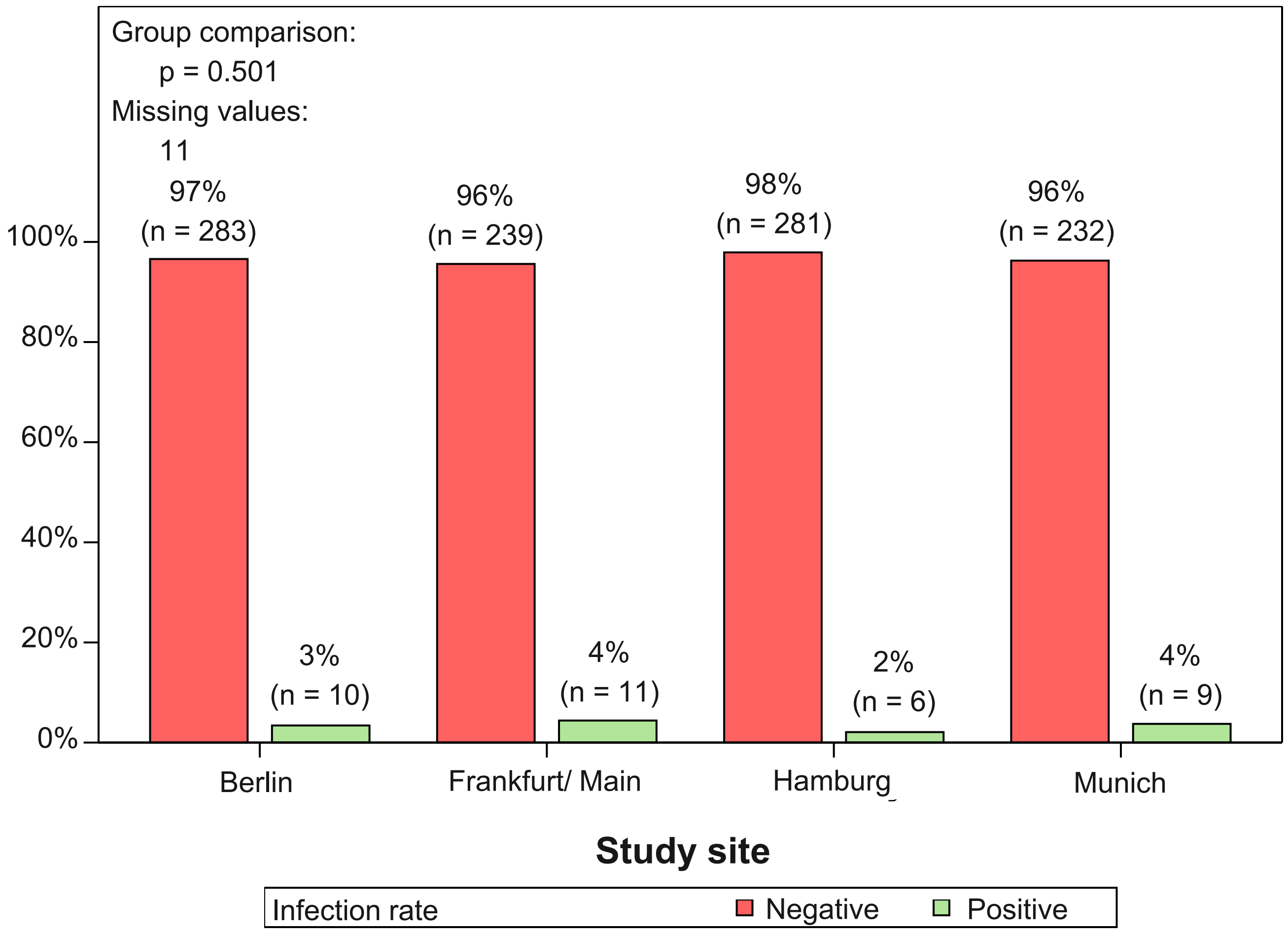

Figure 14: Comparison of employee's residence at different study sites vs. infection rate in the second test series (October 2020).

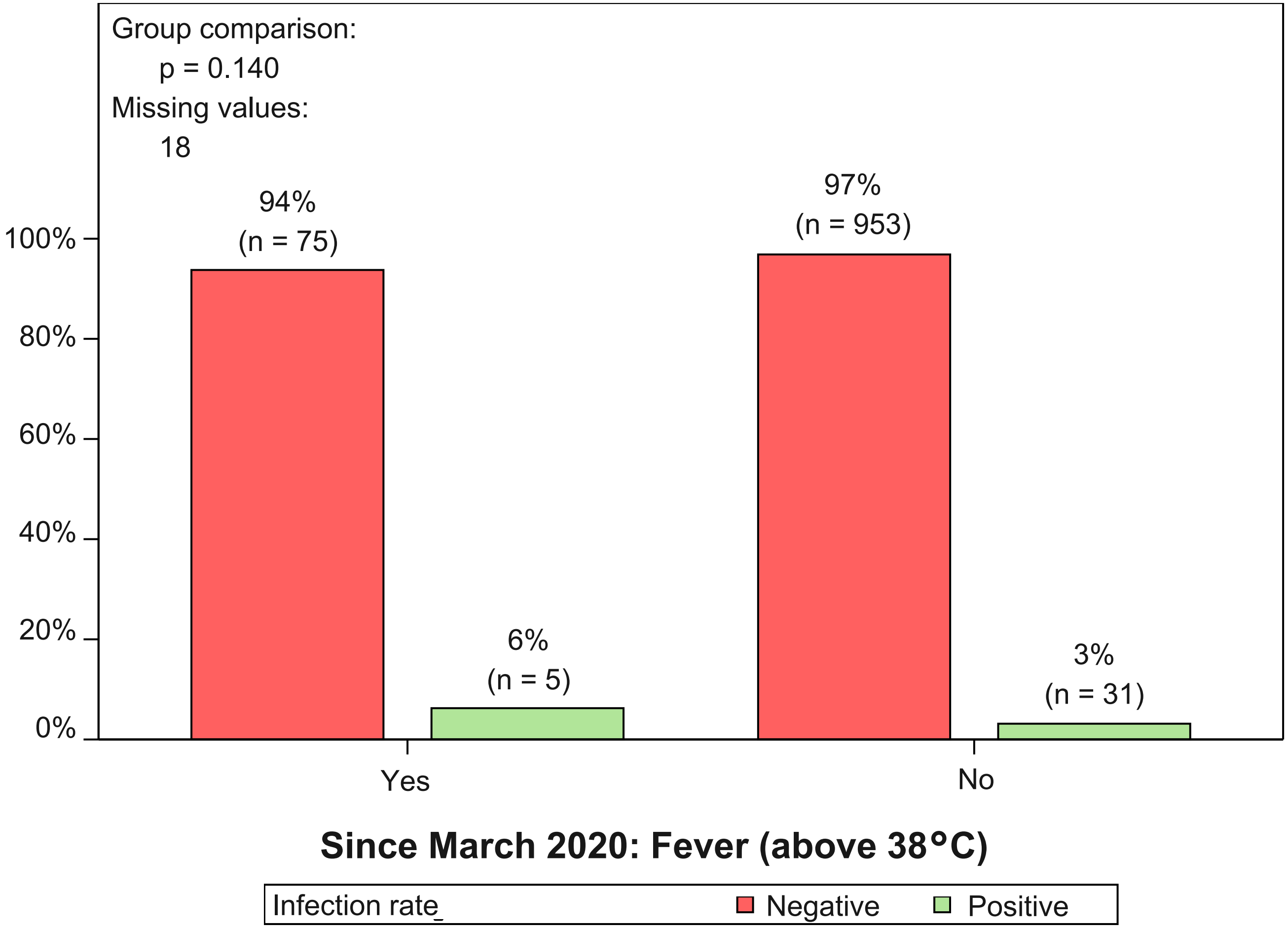

Figure 15: Infection rate vs. reported fever (above 38°C) in second test series (October 2020). Results are based on the symptom reported by participants in the questionnaire during the second test series.

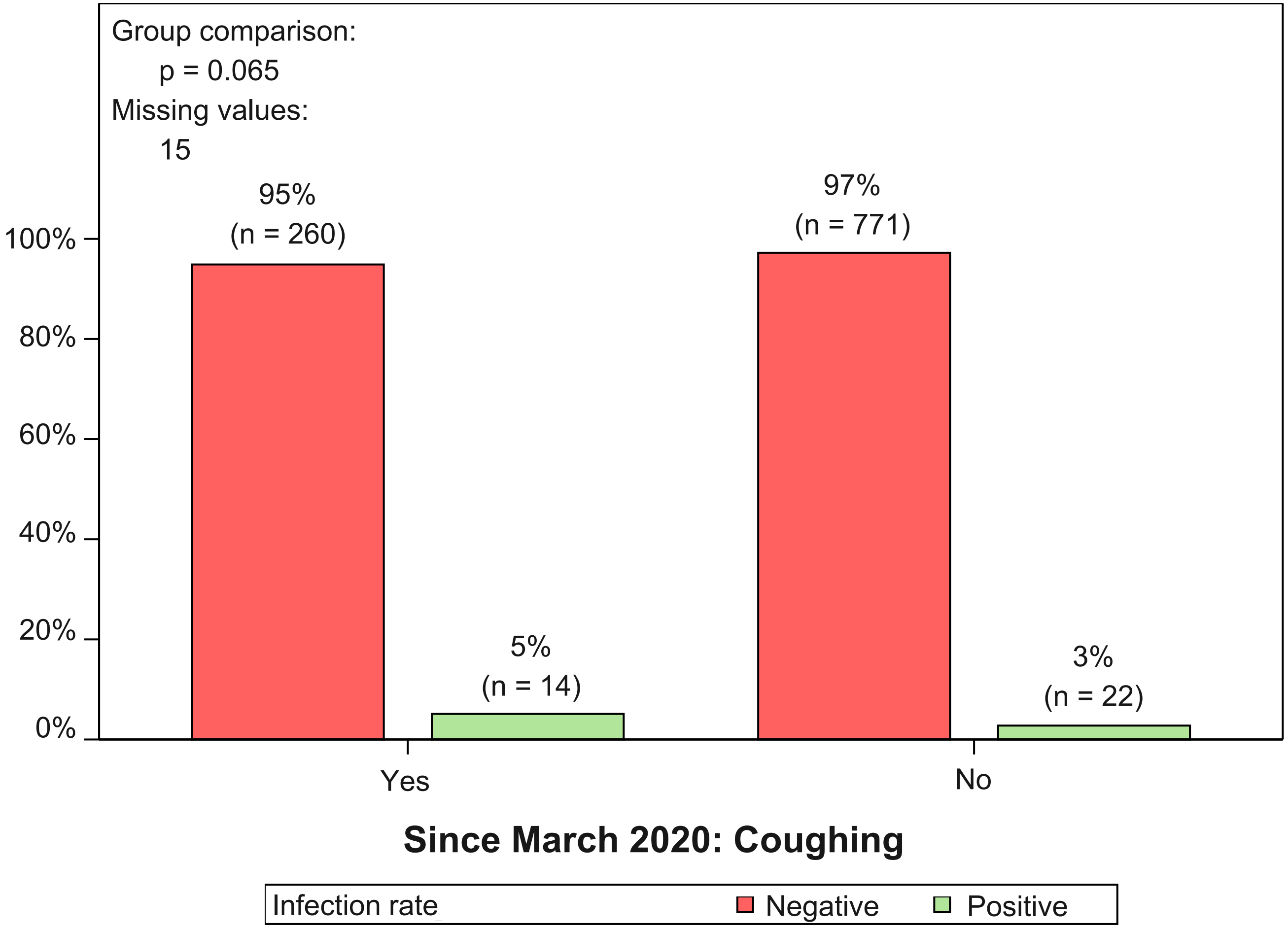

Figure 16: Infection rate vs. coughing (since March 2020) in second test series (October 2020). Results are based on the symptom reported by participants in the questionnaire during the second test series.

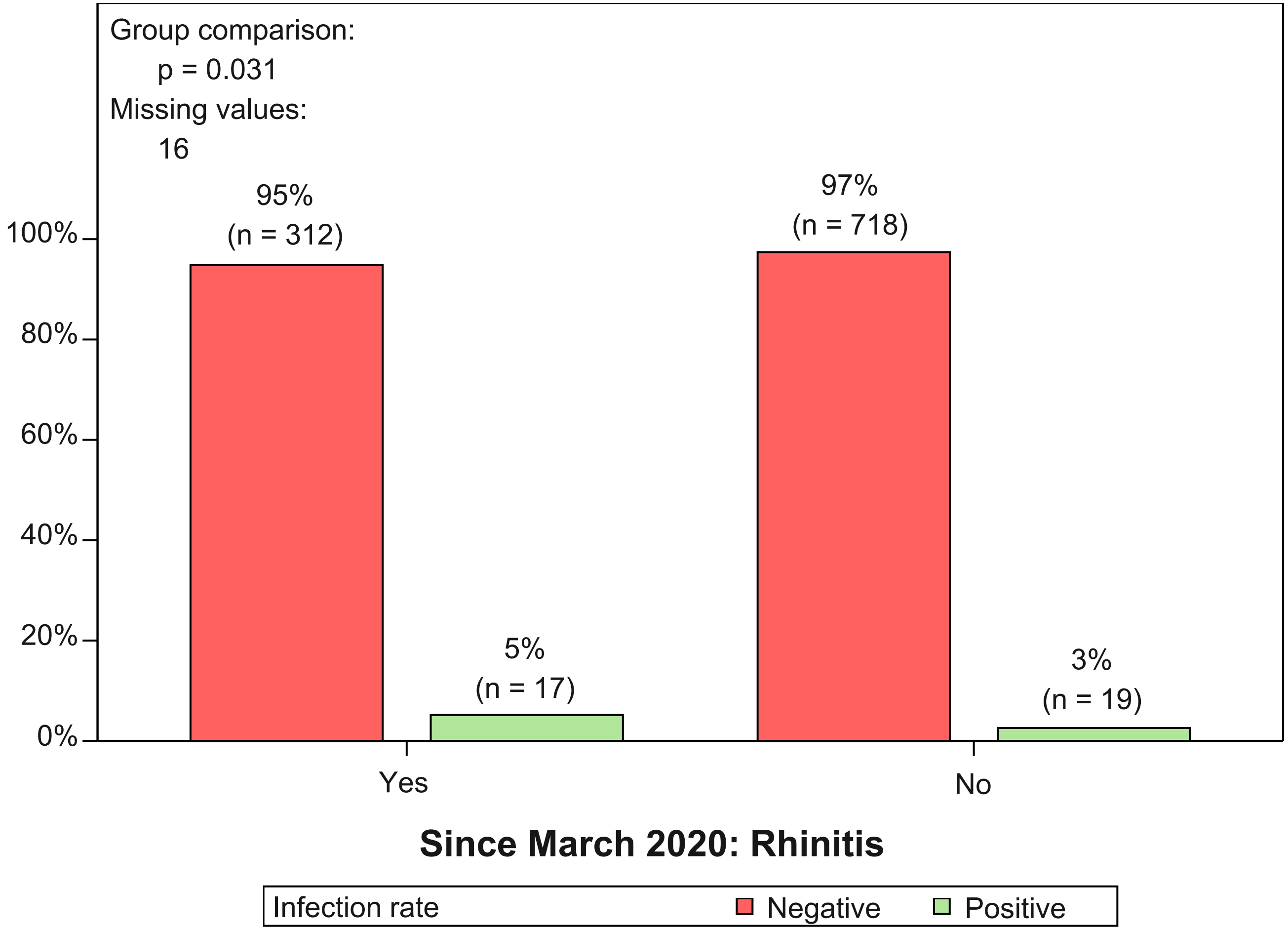

Figure 17: Infection rate vs. rhinitis (since March 2020) in second test series (October 2020). Results are based on the symptom reported by participants in the questionnaire during the second test series.

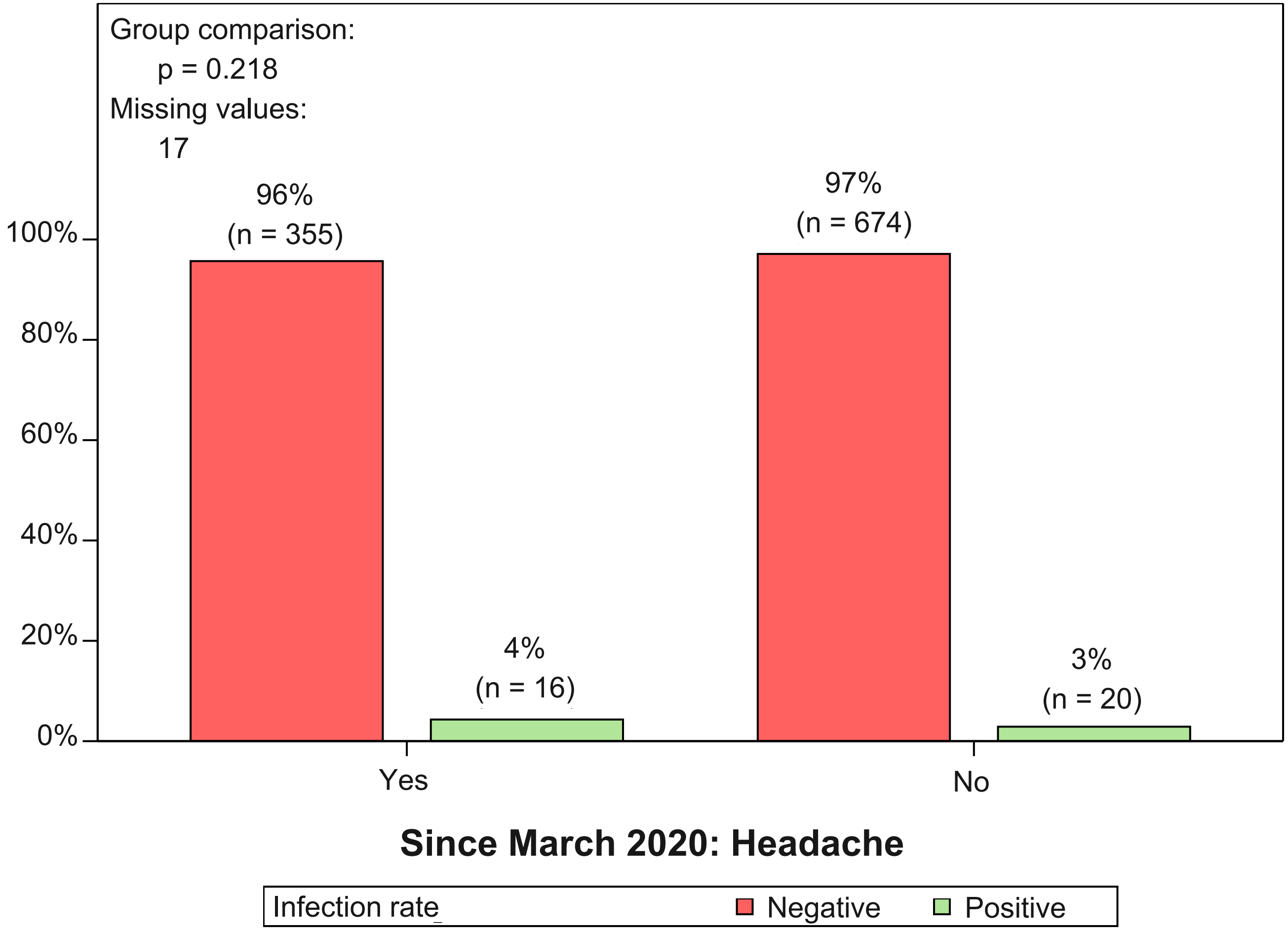

Figure 18: Infection rate vs. headache (since March 2020) in the second test series (October 2020). Results are based on the symptom reported by participants in the questionnaire during the second test series.

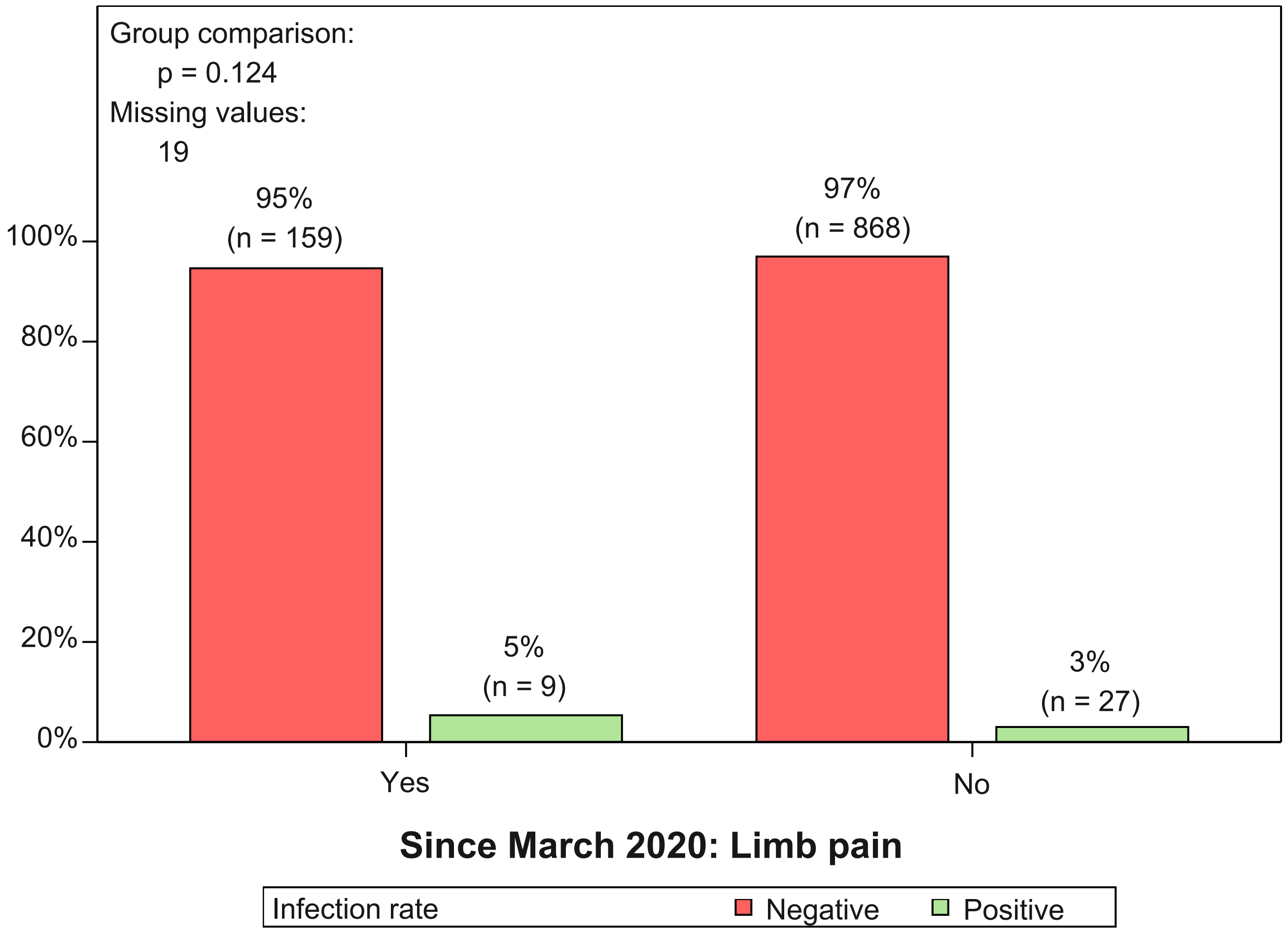

Figure 19: Infection rate vs. reported limb pain (since March 2020) in the second test series (October 2020). Results are based on the symptom reported by participants in the questionnaire during the second test series.

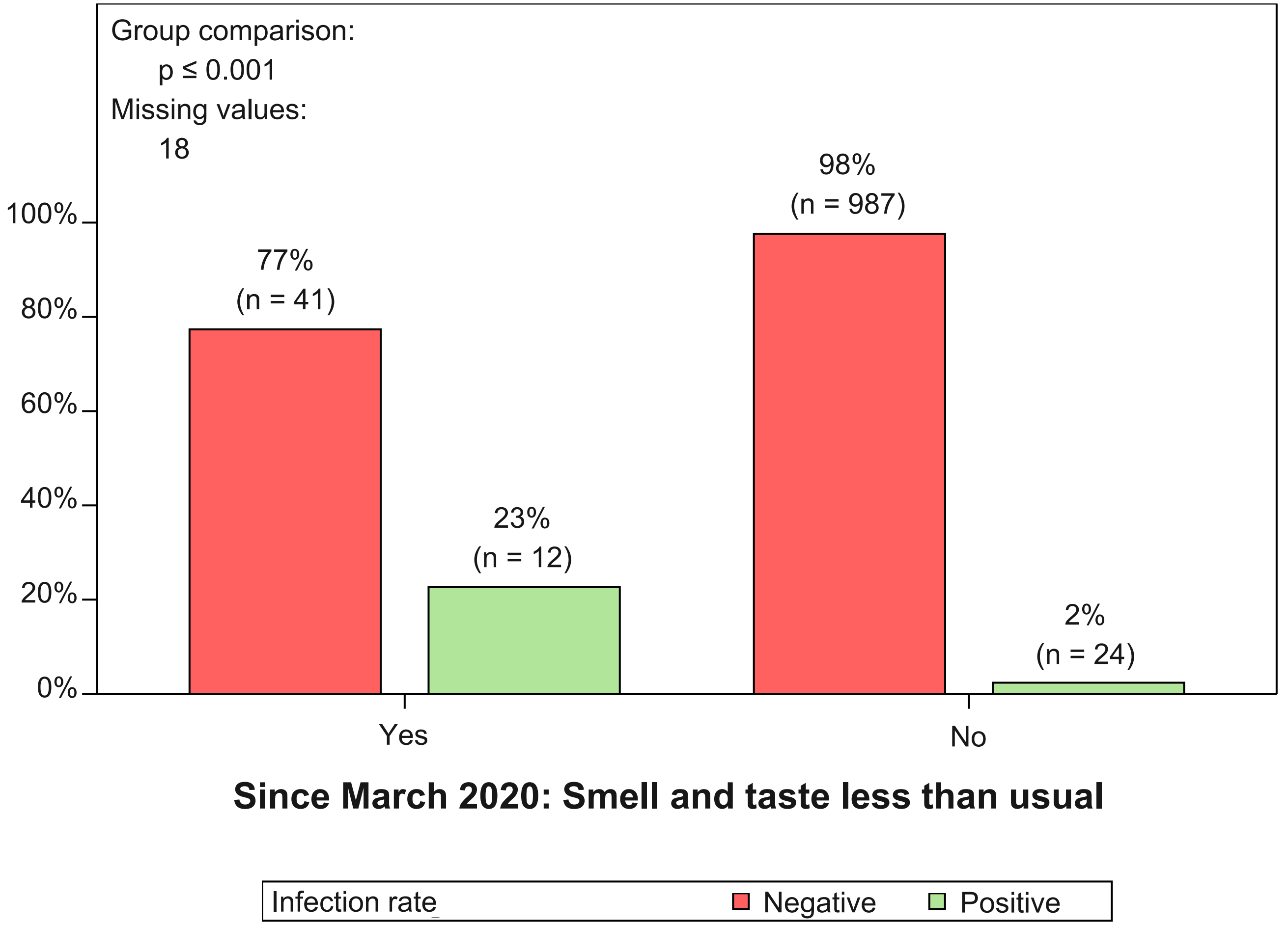

Figure 20: Infection rate vs. reported anosmia and ageusia (since March 2020) in the second test series (October 2020). Results are based on the symptom reported by participants in the questionnaire during the second test series.

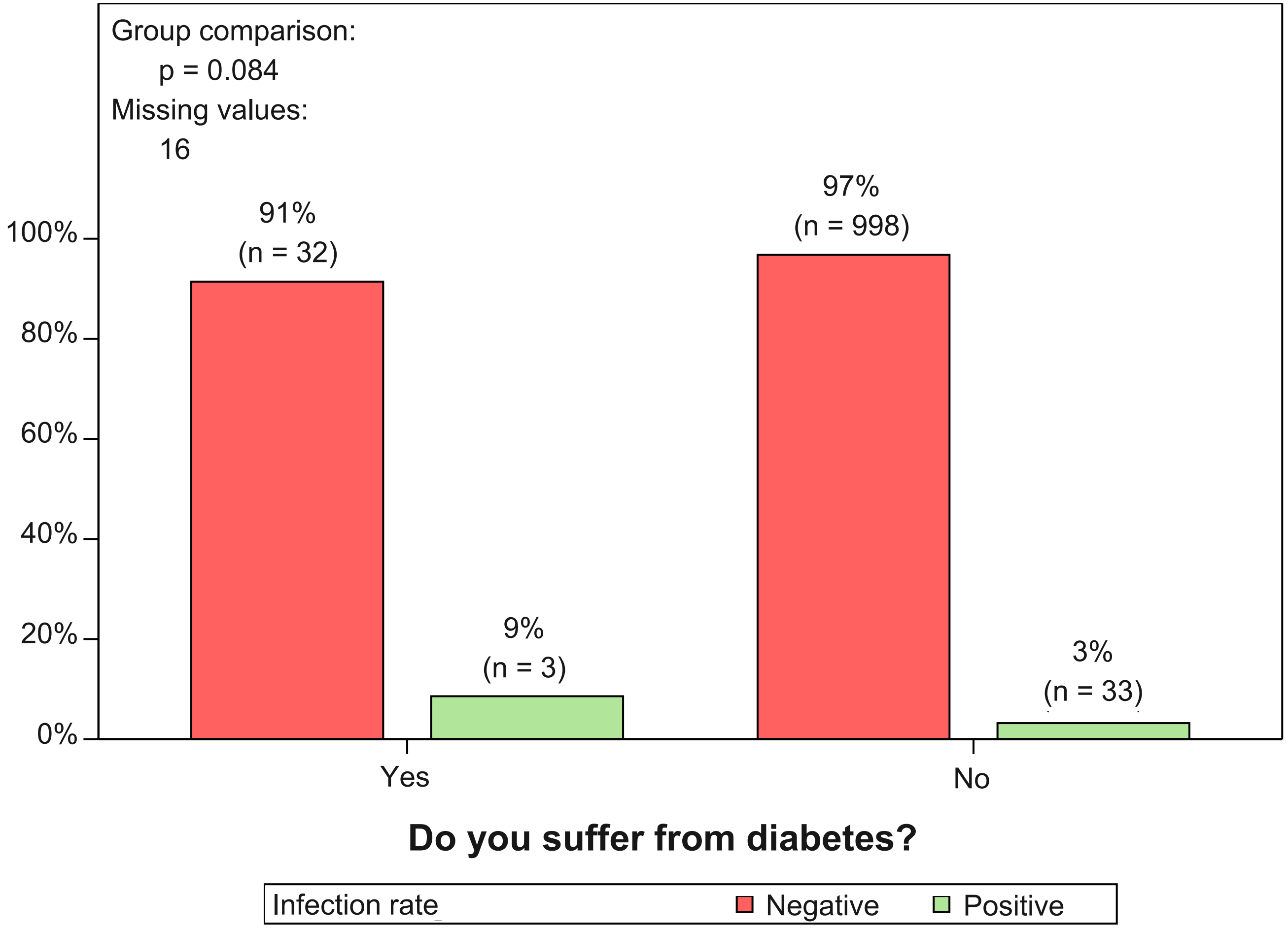

Figure 21: Infection rate vs. reported diabetes (second test series: October 2020). Results are based on answers of participants in the questionnaire during the second test series.

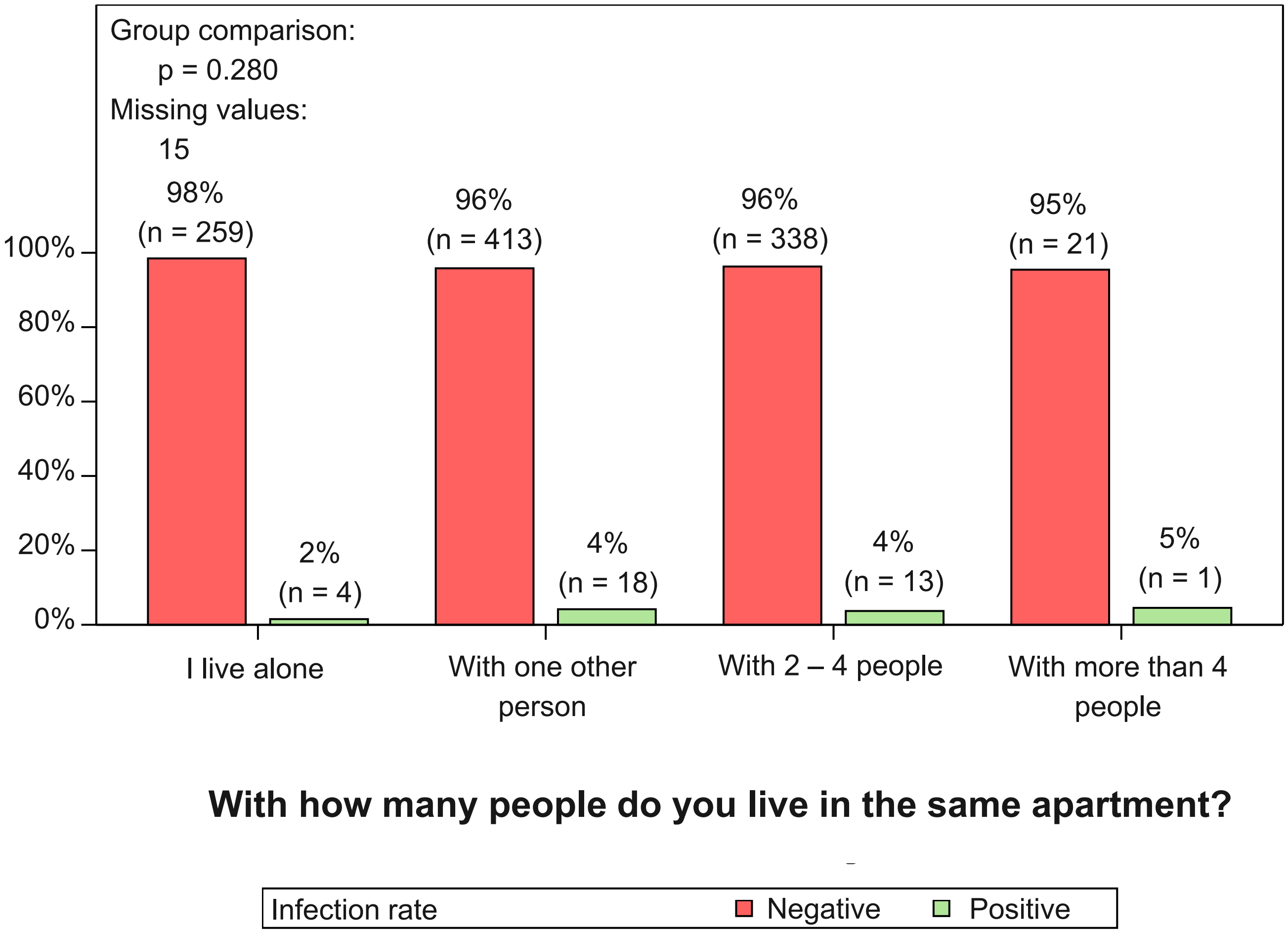

Figure 22: Infection rate vs. number of people in same apartment (second test series: October 2020). Results are based on answers of participants in the questionnaire during the second test series.

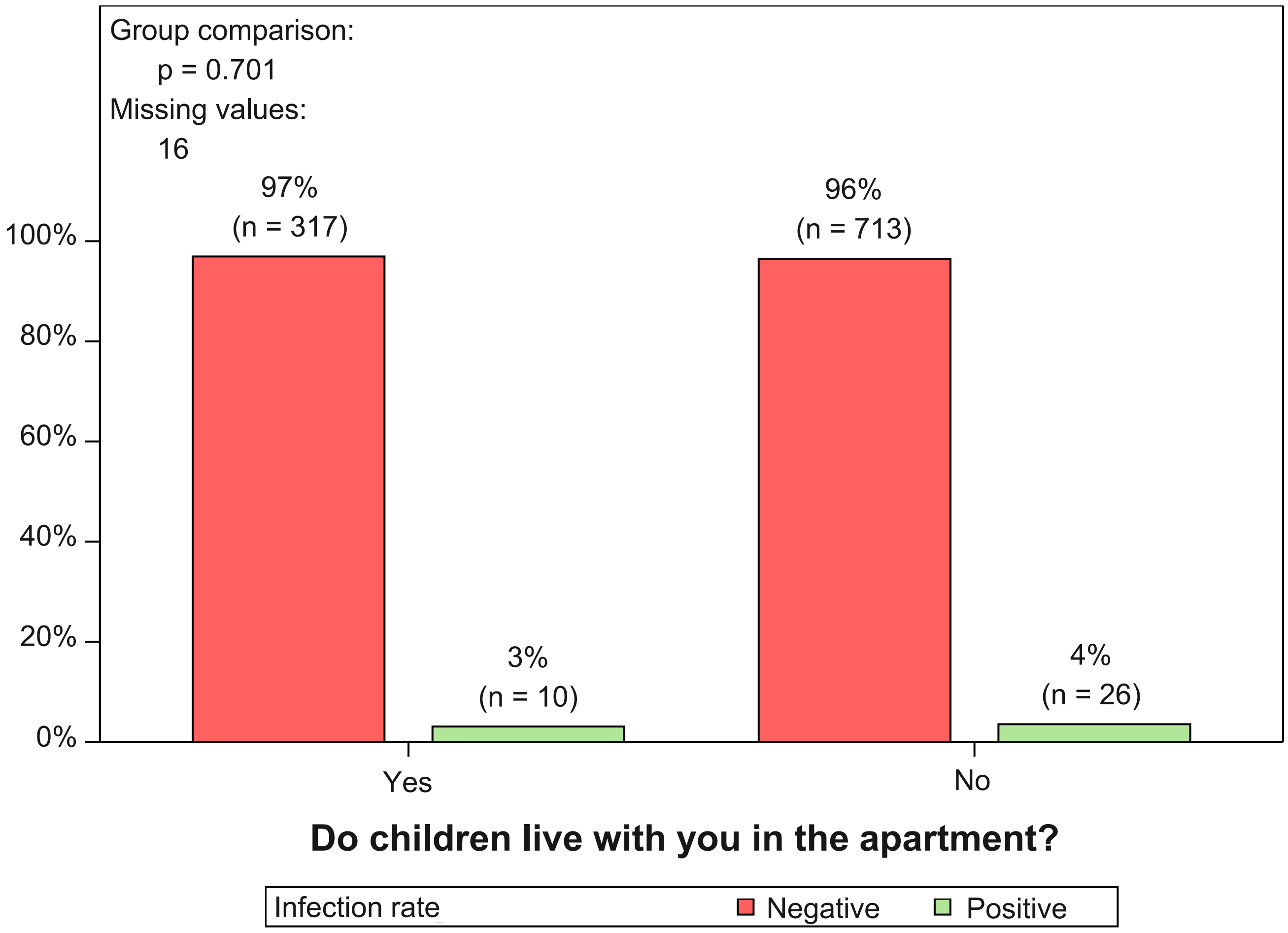

Figure 23: Infection rate vs. children in same apartment (second test series: October 2020). Results are based on answers of participants in the questionnaire during the second test series.

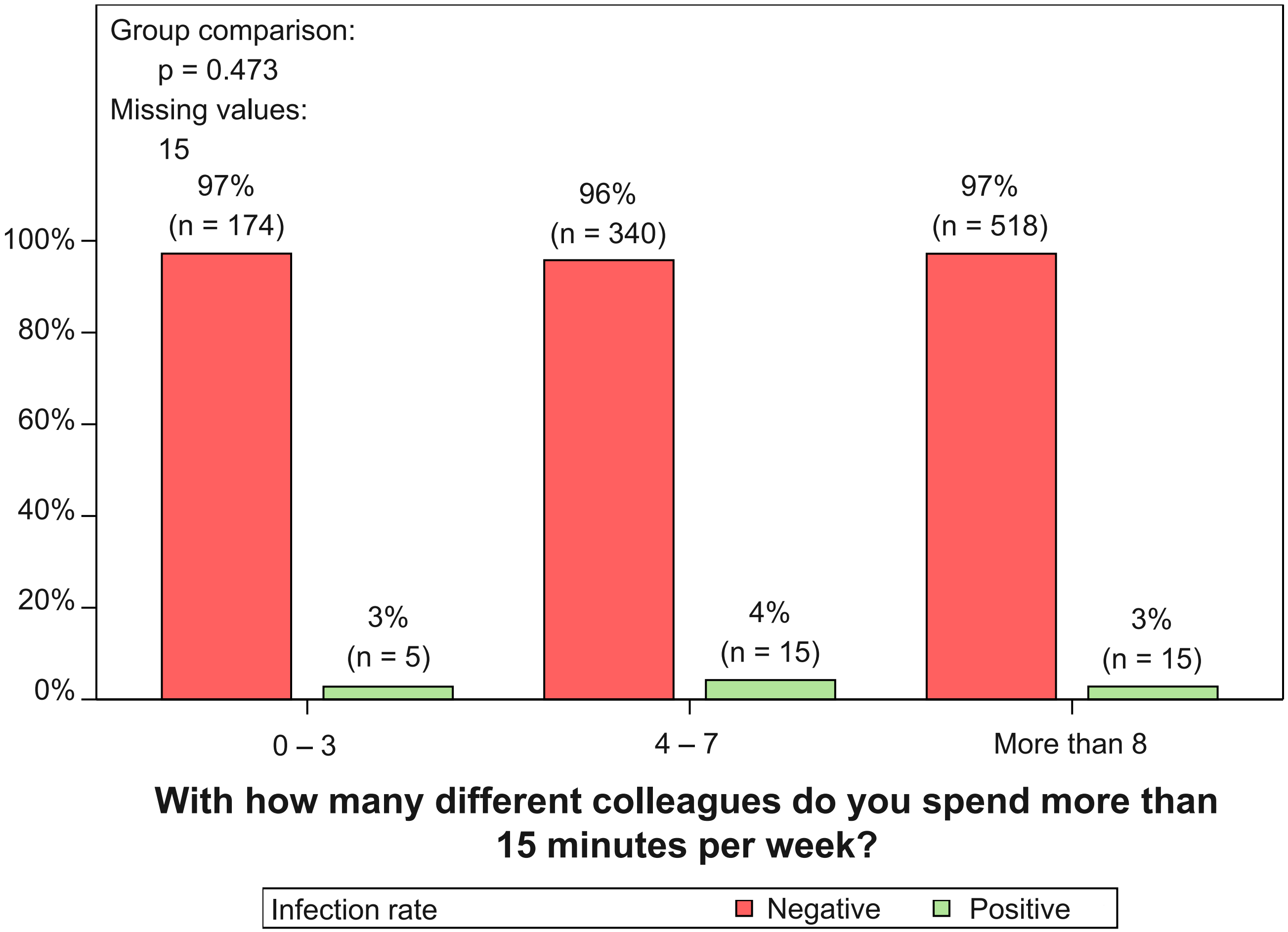

Figure 24: Infection rate vs. number of occupational contacts per week lasting more than 15 minutes (second test series: October 2020). Results are based on answers of participants in the questionnaire during the second test series.

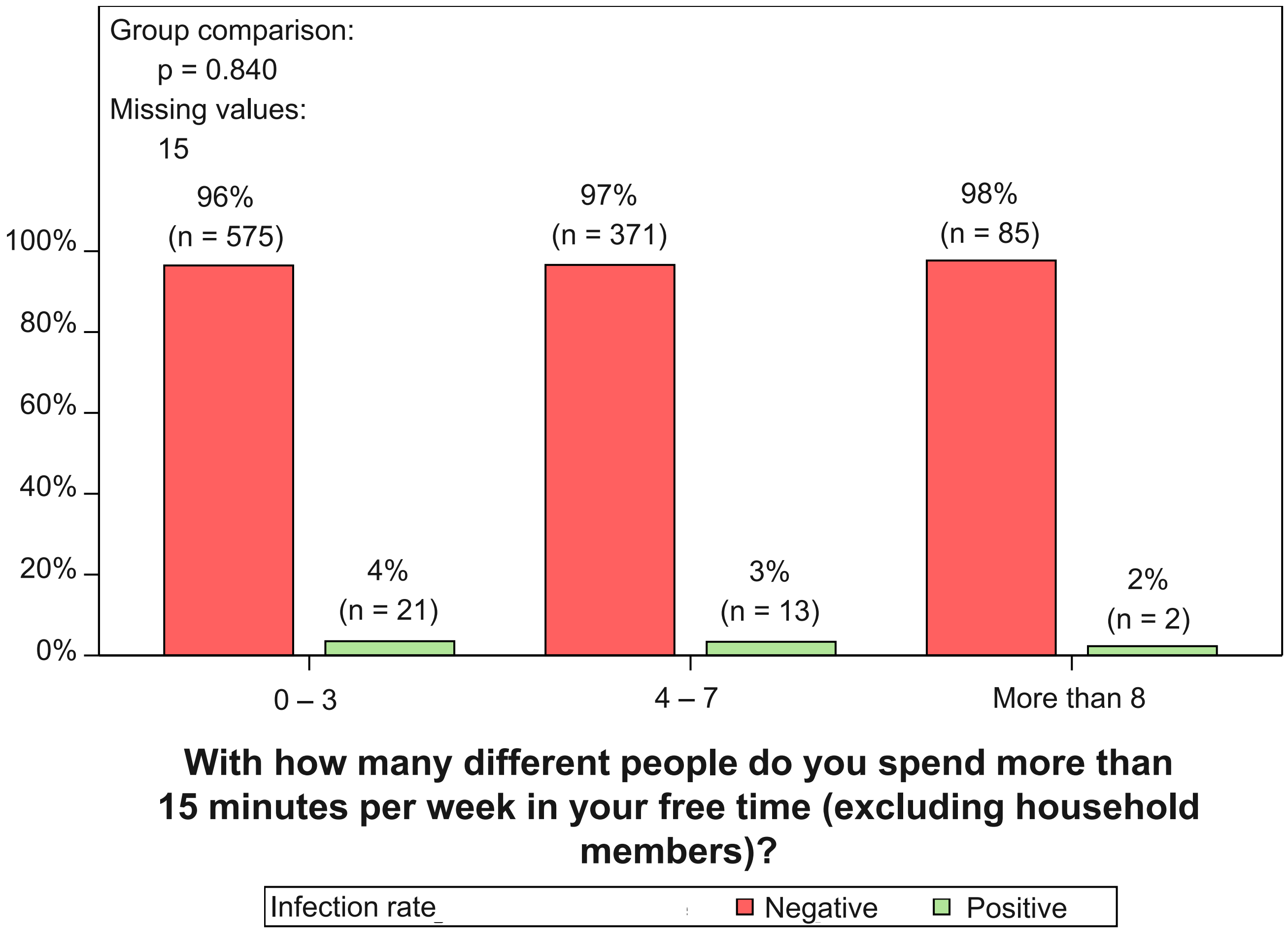

Figure 25: Infection rate vs. number of contacts in free time per week lasting more than 15 minutes (second test series: October 2020). Results are based on answers of participants in the questionnaire during the second test series
