# Supplementary material for: Longitudinal study of SARS-CoV-2 infections in different employee groups of long distance train services from June 2020 until February 2021 in Germany
Source: Epidemiol Infect. 2022 Apr 20;150:e88. doi: 10.1017/S095026882200070X (PMC9095852; doi:10.1017/S095026882200070X)
Supplement: Supplementary file 1 [file hygsup.zip › S095026882200070Xsup003.pdf]

01 Supplementary Material

Longitudinal study of SARS-CoV-2 infections in different employee groups of long distance train services in Germany

Authors:

HyoungJin Kim[1,5] , Robert Schultz-Heienbrok[1*], Markus Uhle[1], Jenni Neubert[1], Fabian Ball[2], Matthes Metz[3], Christian Gravert[4]

[1]Charité Research Organisation GmbH, Germany, [2]DB Fernverkehr AG, Germany, [3]Department of Biostatistics, GCP-Service International Ltd. & Co. KG, Germany,

[4]Deutsche Bahn AG, Germany, [5]Janssen-Cilag GmbH, Germany

Author for correspondence:

Robert Schultz-Heienbrok E-Mail: robert.schultz-heienbrok@charite-research.org

Table of Content

Table 1: Overview of participants in groups and subgroups - Total ... 3
Table 2: Demographics - Total ... 3
Table 3: Demographics - Subgroup: Berlin ... 4
Table 4: Demographics - Subgroup: Hamburg ... 4
Table 5: Demographics - Subgroup: Munich ... 5
Table 6: Demographics - Subgroup: Frankfurt/ Main ... 5
Table 7: Demographics - Subgroup: In 1st test series enrolled ... 6
Table 8: Demographics - Subgroup: In 2nd test series enrolled ... 6
Table 9: Demographics - Subgroup: In 3rd test series enrolled ... 7
Table 10: Epidemiologic baseline characteristics - Total ... 8
Table 11: Epidemiologic baseline characteristics - Subgroup Berlin ... 9
Table 12: Epidemiologic baseline characteristics - Subgroup Hamburg ... 10
Table 13: Epidemiologic baseline characteristics - Subgroup Munich ... 11
Table 14: Epidemiologic baseline characteristics - Subgroup Frankfurt am Main ... 12
Table 15: Epidemiologic baseline characteristics - Subgroup: In 1st test series enrolled ... 13
Table 16: Epidemiologic baseline characteristics - Subgroup: In 2nd test series enrolled ... 14
Table 17: Epidemiologic baseline characteristics - Subgroup: In 3rd test series enrolled ... 15
Table 18: Questionnaire analysis - Total ... 16
Table 19: Questionnaire analysis - Total (continued) ... 17
Table 20: Questionnaire analysis - Total (continued) ... 18
Table 22: Primary objectives - Total ... 19
Table 23: Primary objectives - Total (continued) ... 20
Table 24: Primary objectives – Subgroup: Age under 45 ... 21
Table 25: Primary objectives - Subgroup: Age 45 or more ... 22
Table 26: Primary objectives - Subgroup: Berlin ... 23
Table 27: Primary objectives - Subgroup: Hamburg ... 24
Table 28: Primary objectives - Subgroup: Munich ... 25
Table 29: Primary objectives - Subgroup: Frankfurt am Main ... 26
Table 30: Primary objectives - Subgroup: In 1st test series enrolled ... 27
Table 31: Primary objectives - Subgroup: In 2nd test series enrolled ... 28
Table 32: Primary objectives - Subgroup: In 3rd test series enrolled ... 29
Table 33: Course of SARS-CoV-2 infections ... 30
Table 34: Positive and negative predictive values ... 30
Table 35: Antibody detection - 1st test series/3rd test series ... 31
Table 36: Antibody detection - 2nd test series/3rd test series ... 31

Table 1: Overview of participants in groups and subgroups - Total

	Train attendants		Train drivers		Maintenance workers		Total	
	n	(%)	n	(%)	n	(%)	n	(%)
**In total**								
	589	(56.8)	230	(22.2)	218	(21.0)	1037	(100.0)
**Subgroup: Study site**								
Berlin	157	(26.7)	62	(27.0)	57	(26.1)	276	(26.6)
Frankfurt	154	(26.1)	57	(24.8)	24	(11.0)	235	(22.7)
Hamburg	151	(25.6)	56	(24.3)	92	(42.2)	299	(28.8)
Munich	127	(21.6)	55	(23.9)	45	(20.6)	227	(21.9)
**Subgroup: First study enrolment**								
1st test series	393	(66.7)	167	(72.6)	132	(60.6)	692	(66.7)
2nd test series	98	(16.6)	39	(17.0)	58	(26.6)	195	(18.8)
3rd test series	98	(16.6)	24	(10.4)	28	(12.8)	150	(14.5)

n: Number of non-missing observations; %: Percentages for response categories based on total number of non-missing observations in the respective group, Percentages for missing observations are based on number of all subjects in the respective group

Table 2: Demographics - Total

	Train attendants		Train drivers		Maintenance workers		Total		
	n	(%)	n	(%)	n	(%)	n	(%)	p-value*
**Gender**									<.0001
Male	282	(47.9)	224	(97.4)	204	(95.3)	710	(68.7)	
Female	307	(52.1)	6	(2.6)	10	(4.7)	323	(31.3)	
Missing	0	(0.0)	0	(0.0)	4	(1.8)	4	(0.4)	
**Age [Years]**									<.0001
n	585		223		213		1021		
Missing	4	(0.7)	7	(3.0)	5	(2.3)	16	(1.5)	
Mean value	43.63		47.58		45.91		44.97		
SD	10.54		11.10		11.31		10.94		
Min	18		20		21		18		
Q1	36.0		39.0		36.0		37.0		
Median	45.0		50.0		48.0		47.0		
Q3	51.0		56.0		55.0		53.0		
Max	66		65		64		66		

n: Number of non-missing observations; %: Percentages for response categories based on total number of non-missing observations in the respective group, Percentages for missing observations are based on number of all subjects in the respective group; SD: Standard deviation; Q1: Lower quartile; Q3: Upper quartile
*: Chi-square test for Gender, ANOVA (global null hypothesis) for Age.

Table 3: Demographics - Subgroup: Berlin

	Train attendants		Train drivers		Maintenance workers		Total		
	n	(%)	n	(%)	n	(%)	n	(%)	p-value*
**Gender**									<.0001
Male	79	(50.3)	61	(98.4)	55	(96.5)	195	(70.7)	
Female	78	(49.7)	1	(1.6)	2	(3.5)	81	(29.3)	
Missing	0	(0.0)	0	(0.0)	0	(0.0)	0	(0.0)	
**Age [Years]**									0.0012
n	157		60		57		274		
Missing	0	(0.0)	2	(3.2)	0	(0.0)	2	(0.7)	
Mean value	46.43		51.83		45.68		47.46		
SD	9.91		9.33		12.64		10.64		
Min	20		25		21		20		
Q1	40.0		49.0		33.0		40.0		
Median	48.0		54.0		50.0		49.5		
Q3	54.0		59.0		57.0		56.0		
Max	63		63		62		63		

n: Number of non-missing observations; %: Percentages for response categories based on total number of non-missing observations in the respective group, Percentages for missing observations are based on number of all subjects in the respective group; SD: Standard deviation; Q1: Lower quartile; Q3: Upper quartile
*: Chi-square test for Gender, ANOVA (global null hypothesis) for Age.

Table 4: Demographics - Subgroup: Hamburg

	Train attendants		Train drivers		Maintenance workers		Total		
	n	(%)	n	(%)	n	(%)	n	(%)	p-value*
**Gender**									<.0001
Male	63	(41.7)	53	(94.6)	86	(94.5)	202	(67.8)	
Female	88	(58.3)	3	(5.4)	5	(5.5)	96	(32.2)	
Missing	0	(0.0)	0	(0.0)	1	(1.1)	1	(0.3)	
**Age [Years]**									<.0001
n	151		54		89		294		
Missing	0	(0.0)	2	(3.6)	3	(3.3)	5	(1.7)	
Mean value	42.83		49.91		46.39		45.21		
SD	9.98		9.64		11.12		10.60		
Min	21		24		22		21		
Q1	35.0		46.0		38.0		37.0		
Median	44.0		51.5		49.0		48.0		
Q3	50.0		56.0		54.0		53.0		
Max	64		64		63		64		

n: Number of non-missing observations; %: Percentages for response categories based on total number of non-missing observations in the respective group, Percentages for missing observations are based on number of all subjects in the respective group; SD: Standard deviation; Q1: Lower quartile; Q3: Upper quartile
*: Chi-square test for Gender, ANOVA (global null hypothesis) for Age.

Table 5: Demographics - Subgroup: Munich

	Train attendants		Train drivers		Maintenance workers		Total		
	n	(%)	n	(%)	n	(%)	n	(%)	p-value*
**Gender**									<.0001
Male	60	(47.2)	53	(96.4)	39	(92.9)	152	(67.9)	
Female	67	(52.8)	2	(3.6)	3	(7.1)	72	(32.1)	
Missing	0	(0.0)	0	(0.0)	3	(6.7)	3	(1.3)	
**Age [Years]**									0.1169
n	126		53		43		222		
Missing	1	(0.8)	2	(3.6)	2	(4.4)	5	(2.2)	
Mean value	42.84		44.25		46.79		43.94		
SD	10.49		11.61		10.71		10.87		
Min	20		20		22		20		
Q1	35.0		32.0		37.0		37.0		
Median	43.0		48.0		48.0		44.0		
Q3	50.0		52.0		57.0		52.0		
Max	66		65		64		66		

n: Number of non-missing observations; %: Percentages for response categories based on total number of non-missing observations in the respective group, Percentages for missing observations are based on number of all subjects in the respective group; SD: Standard deviation; Q1: Lower quartile; Q3: Upper quartile
*: Chi-square test for Gender, ANOVA (global null hypothesis) for Age.

Table 6: Demographics - Subgroup: Frankfurt/ Main

	Train attendants		Train drivers		Maintenance workers		Total		
	n	(%)	n	(%)	n	(%)	n	(%)	p-value*
**Gender**									<.0001
Male	80	(51.9)	57	(100.0)	24	(100.0)	161	(68.5)	
Female	74	(48.1)	0	(0.0)	0	(0.0)	74	(31.5)	
Missing	0	(0.0)	0	(0.0)	0	(0.0)	0	(0.0)	
**Age [Years]**									0.5886
n	151		56		24		231		
Missing	3	(1.9)	1	(1.8)	0	(0.0)	4	(1.7)	
Mean value	42.16		43.95		43.08		42.69		
SD	11.28		11.74		9.85		11.24		
Min	18		23		27		18		
Q1	34.0		35.0		35.5		35.0		
Median	43.0		46.0		42.0		43.0		
Q3	51.0		55.0		50.5		51.0		
Max	63		62		63		63		

n: Number of non-missing observations; %: Percentages for response categories based on total number of non-missing observations in the respective group, Percentages for missing observations are based on number of all subjects in the respective group; SD: Standard deviation; Q1: Lower quartile; Q3: Upper quartile
*: Chi-square test for Gender, ANOVA (global null hypothesis) for Age.

Table 7: Demographics - Subgroup: In 1st test series enrolled

	Train attendants		Train drivers		Maintenance workers		Total		
	n	(%)	n	(%)	n	(%)	n	(%)	p-value*
**Gender**									<.0001
Male	181	(46.1)	164	(98.2)	123	(94.6)	468	(67.8)	
Female	212	(53.9)	3	(1.8)	7	(5.4)	222	(32.2)	
Missing	0	(0.0)	0	(0.0)	2	(1.5)	2	(0.3)	
**Age [Years]**									<.0001
n	390		163		132		685		
Missing	3	(0.8)	4	(2.4)	0	(0.0)	7	(1.0)	
Mean value	43.83		47.84		46.70		45.34		
SD	10.26		10.70		11.17		10.68		
Min	19		23		22		19		
Q1	37.0		41.0		37.0		37.0		
Median	45.0		51.0		49.0		48.0		
Q3	51.0		55.0		55.5		53.0		
Max	64		65		63		65		

n: Number of non-missing observations; %: Percentages for response categories based on total number of non-missing observations in the respective group, Percentages for missing observations are based on number of all subjects in the respective group; SD: Standard deviation; Q1: Lower quartile; Q3: Upper quartile
*: Chi-square test for Gender, ANOVA (global null hypothesis) for Age.

Table 8: Demographics - Subgroup: In 2nd test series enrolled

	Train attendants		Train drivers		Maintenance workers		Total		
	n	(%)	n	(%)	n	(%)	n	(%)	p-value*
**Gender**									<.0001
Male	46	(46.9)	37	(94.9)	58	(100.0)	141	(72.3)	
Female	52	(53.1)	2	(5.1)	0	(0.0)	54	(27.7)	
Missing	0	(0.0)	0	(0.0)	0	(0.0)	0	(0.0)	
**Age [Years]**									0.2209
n	98		39		58		195		
Missing	0	(0.0)	0	(0.0)	0	(0.0)	0	(0.0)	
Mean value	43.74		47.41		44.21		44.62		
SD	10.92		11.99		11.45		11.33		
Min	18		20		21		18		
Q1	36.0		37.0		36.0		36.0		
Median	45.0		50.0		45.5		46.0		
Q3	52.0		58.0		53.0		54.0		
Max	63		63		64		64		

n: Number of non-missing observations; %: Percentages for response categories based on total number of non-missing observations in the respective group, Percentages for missing observations are based on number of all subjects in the respective group; SD: Standard deviation; Q1: Lower quartile; Q3: Upper quartile
*: Chi-square test for Gender, ANOVA (global null hypothesis) for Age.

Table 9: Demographics - Subgroup: In 3rd test series enrolled

	Train attendants		Train drivers		Maintenance workers		Total		
	**n**	**(%)**	**n**	**(%)**	**n**	**(%)**	**n**	**(%)**	**p-value***
**Gender**									<.0001
Male	55	(56.1)	23	(95.8)	23	(88.5)	101	(68.2)	
Female	43	(43.9)	1	(4.2)	3	(11.5)	47	(31.8)	
Missing	0	(0.0)	0	(0.0)	2	(7.1)	2	(1.3)	
**Age [Years]**									0.3479
n	97		21		23		141		
Missing	1	(1.0)	3	(12.5)	5	(17.9)	9	(6.0)	
Mean value	42.70		45.90		45.65		43.66		
SD	11.30		12.81		11.74		11.61		
Min	20		24		23		20		
Q1	34.0		33.0		34.0		34.0		
Median	43.0		51.0		49.0		44.0		
Q3	52.0		55.0		56.0		53.0		
Max	66		62		62		66		

n: Number of non-missing observations; %: Percentages for response categories based on total number of non-missing observations in the respective group, Percentages for missing observations are based on number of all subjects in the respective group; SD: Standard deviation; Q1: Lower quartile; Q3: Upper quartile
*: Chi-square test for Gender, ANOVA (global null hypothesis) for Age.

Table 10: Epidemiologic baseline characteristics - Total

	Train attendants		Train drivers		Maintenance workers		Total		
	n	(%)	n	(%)	n	(%)	n	(%)	p-value*
**Do you suffer from a disease of the cardiovascular system?**									0.0004
Yes	40	(6.9)	25	(11.1)	34	(16.2)	99	(9.7)	
No	541	(93.1)	200	(88.9)	176	(83.8)	917	(90.3)	
Missing	8	(1.4)	5	(2.2)	8	(3.7)	21	(2.0)	
**Do you suffer from diabetes?**									0.1347
Yes	16	(2.7)	9	(4.0)	12	(5.7)	37	(3.6)	
No	568	(97.3)	218	(96.0)	198	(94.3)	984	(96.4)	
Missing	5	(0.8)	3	(1.3)	8	(3.7)	16	(1.5)	
**Have you smoked regularly in the past 12 months?**									<.0001
Yes, e-cigarette	15	(2.6)	4	(1.8)	5	(2.4)	24	(2.4)	
Yes, up to 10 cigarettes/ day	71	(12.3)	12	(5.3)	21	(10.0)	104	(10.2)	
Yes, more than 10 cigarettes/ day	115	(19.9)	15	(6.6)	25	(11.8)	155	(15.3)	
No	378	(65.3)	195	(86.3)	160	(75.8)	733	(72.1)	
Missing	10	(1.7)	4	(1.7)	7	(3.2)	21	(2.0)	
**With how many people do you live in the same apartment?**									0.1032
I live alone	150	(25.6)	62	(27.3)	47	(22.2)	259	(25.3)	
With one other person	228	(38.9)	97	(42.7)	79	(37.3)	404	(39.4)	
With 2 - 4 people	192	(32.8)	68	(30.0)	81	(38.2)	341	(33.3)	
With more than 4 people	16	(2.7)	0	(0.0)	5	(2.4)	21	(2.0)	
Missing	3	(0.5)	3	(1.3)	6	(2.8)	12	(1.2)	
**Do children live with you in the apartment?**									0.3069
0	403	(69.0)	172	(76.1)	146	(68.9)	721	(70.5)	
1	104	(17.8)	39	(17.3)	41	(19.3)	184	(18.0)	
2	62	(10.6)	14	(6.2)	21	(9.9)	97	(9.5)	
3	13	(2.2)	1	(0.4)	4	(1.9)	18	(1.8)	
4	2	(0.3)	0	(0.0)	0	(0.0)	2	(0.2)	
Missing	5	(0.8)	4	(1.7)	6	(2.8)	15	(1.4)	

n: Number of non-missing observations; %: Percentages for response categories based on total number of non-missing observations in the respective group, Percentages for missing observations are based on number of all subjects in the respective group; *: Chi-squared test.

Table 11: Epidemiologic baseline characteristics - Subgroup Berlin

	Train attendants		Train drivers		Maintenance workers		Total		
	n	(%)	n	(%)	n	(%)	n	(%)	p-value*
**Do you suffer from a disease of the cardiovascular system?**									0.2330
Yes	12	(7.8)	6	(10.0)	9	(15.8)	27	(10.0)	
No	141	(92.2)	54	(90.0)	48	(84.2)	243	(90.0)	
Missing	4	(2.5)	2	(3.2)	0	(0.0)	6	(2.2)	
**Do you suffer from diabetes?**									0.3661
Yes	4	(2.5)	4	(6.6)	2	(3.6)	10	(3.6)	
No	153	(97.5)	57	(93.4)	54	(96.4)	264	(96.4)	
Missing	0	(0.0)	1	(1.6)	1	(1.8)	2	(0.7)	
**Have you smoked regularly in the past 12 months?**									0.0519
Yes, E-cigarette	1	(0.6)	1	(1.6)	1	(1.8)	3	(1.1)	
Yes, up to 10 cigarettes/ day	14	(9.0)	2	(3.3)	8	(14.3)	24	(8.8)	
Yes, more than 10 cigarettes/ day	34	(21.9)	5	(8.2)	7	(12.5)	46	(16.9)	
No	106	(68.4)	53	(86.9)	40	(71.4)	199	(73.2)	
Missing	2	(1.3)	1	(1.6)	1	(1.8)	4	(1.4)	
**With how many people do you live in the same apartment?**									0.3518
I live alone	38	(24.2)	15	(24.6)	12	(21.1)	65	(23.6)	
With one other person	62	(39.5)	32	(52.5)	22	(38.6)	116	(42.2)	
With 2 - 4 people	52	(33.1)	14	(23.0)	22	(38.6)	88	(32.0)	
With more than 4 people	5	(3.2)	0	(0.0)	1	(1.8)	6	(2.2)	
Missing	0	(0.0)	1	(1.6)	0	(0.0)	1	(0.4)	
**Do children live with you in the apartment?**									0.0570
0	101	(64.3)	52	(85.2)	38	(66.7)	191	(69.5)	
1	36	(22.9)	8	(13.1)	11	(19.3)	55	(20.0)	
2	15	(9.6)	0	(0.0)	7	(12.3)	22	(8.0)	
3	5	(3.2)	1	(1.6)	1	(1.8)	7	(2.5)	
4	0	(0.0)	0	(0.0)	0	(0.0)	0	(0.0)	
Missing	101	(64.3)	52	(85.2)	38	(66.7)	191	(69.5)	

n: Number of non-missing observations; %: Percentages for response categories based on total number of non-missing observations in the respective group, Percentages for missing observations are based on number of all subjects in the respective group; *: Chi-squared test.

Table 12: Epidemiologic baseline characteristics - Subgroup Hamburg

	Train attendants		Train drivers		Maintenance workers		Total		
	n	(%)	n	(%)	n	(%)	n	(%)	p-value*
**Do you suffer from a disease of the cardiovascular system?**									0.0005
Yes	8	(5.3)	6	(10.7)	19	(21.8)	33	(11.3)	
No	142	(94.7)	50	(89.3)	68	(78.2)	260	(88.7)	
Missing	1	(0.7)	0	(0.0)	5	(5.4)	6	(2.0)	
**Do you suffer from diabetes?**									0.5449
Yes	6	(4.0)	4	(7.1)	6	(6.8)	16	(5.5)	
No	143	(96.0)	52	(92.9)	82	(93.2)	277	(94.5)	
Missing	2	(1.3)	0	(0.0)	4	(4.3)	6	(2.0)	
**Have you smoked regularly in the past 12 months?**									0.1323
Yes, E-cigarette	4	(2.7)	0	(0.0)	3	(3.4)	7	(2.4)	
Yes, up to 10 cigarettes/ day	17	(11.5)	4	(7.1)	7	(8.0)	28	(9.6)	
Yes, more than 10 cigarettes/ day	28	(18.9)	4	(7.1)	10	(11.4)	42	(14.4)	
No	99	(66.9)	48	(85.7)	68	(77.3)	215	(73.6)	
Missing	3	(2.0)	0	(0.0)	4	(4.3)	7	(2.3)	
**With how many people do you live in the same apartment?**									0.8969
I live alone	38	(25.3)	15	(26.8)	22	(25.0)	75	(25.5)	
With one other person	58	(38.7)	26	(46.4)	35	(39.8)	119	(40.5)	
With 2 - 4 people	53	(35.3)	15	(26.8)	30	(34.1)	98	(33.3)	
With more than 4 people	1	(0.7)	0	(0.0)	1	(1.1)	2	(0.7)	
Missing	1	(0.7)	0	(0.0)	4	(4.3)	5	(1.7)	
**Do children live with you in the apartment?**									0.3509
0	102	(68.5)	43	(76.8)	66	(75.0)	211	(72.0)	
1	27	(18.1)	10	(17.9)	16	(18.2)	53	(18.1)	
2	20	(13.4)	3	(5.4)	6	(6.8)	29	(9.9)	
3	0	(0.0)	0	(0.0)	0	(0.0)	0	(0.0)	
4	0	(0.0)	0	(0.0)	0	(0.0)	0	(0.0)	
Missing	2	(1.3)	0	(0.0)	4	(4.3)	6	(2.0)	

n: Number of non-missing observations; %: Percentages for response categories based on total number of non-missing observations in the respective group, Percentages for missing observations are based on number of all subjects in the respective group; *: Chi-squared test.

Table 13: Epidemiologic baseline characteristics - Subgroup Munich

	Train attendants		Train drivers		Maintenance workers		Total		
	n	(%)	n	(%)	n	(%)	n	(%)	p-value*
**Do you suffer from a disease of the cardiovascular system?**									0.2747
Yes	8	(6.4)	7	(13.2)	5	(11.9)	20	(9.1)	
No	117	(93.6)	46	(86.8)	37	(88.1)	200	(90.9)	
Missing	2	(1.6)	2	(3.6)	3	(6.7)	7	(3.1)	
**Do you suffer from diabetes?**									0.1068
Yes	1	(0.8)	0	(0.0)	2	(4.7)	3	(1.4)	
No	123	(99.2)	53	(100.0)	41	(95.3)	217	(98.6)	
Missing	3	(2.4)	2	(3.6)	2	(4.4)	7	(3.1)	
**With how many people do you live in the same apartment?**									0.6012
I live alone	41	(32.8)	16	(30.2)	10	(23.3)	67	(30.3)	
With one other person	47	(37.6)	19	(35.8)	18	(41.9)	84	(38.0)	
With 2 - 4 people	32	(25.6)	18	(34.0)	14	(32.6)	64	(29.0)	
With more than 4 people	5	(4.0)	0	(0.0)	1	(2.3)	6	(2.7)	
Missing	2	(1.6)	2	(3.6)	2	(4.4)	6	(2.6)	
**Do children live with you in the apartment?**									0.4989
0	99	(79.8)	39	(75.0)	29	(67.4)	167	(76.3)	
1	13	(10.5)	8	(15.4)	8	(18.6)	29	(13.2)	
2	8	(6.5)	5	(9.6)	5	(11.6)	18	(8.2)	
3	4	(3.2)	0	(0.0)	1	(2.3)	5	(2.3)	
4	0	(0.0)	0	(0.0)	0	(0.0)	0	(0.0)	
Missing	3	(2.4)	3	(5.5)	2	(4.4)	8	(3.5)	

n: Number of non-missing observations; %: Percentages for response categories based on total number of non-missing observations in the respective group, Percentages for missing observations are based on number of all subjects in the respective group; *: Chi-squared test.

Table 14: Epidemiologic baseline characteristics - Subgroup Frankfurt am Main

	Train attendants		Train drivers		Maintenance workers		Total		
	n	(%)	n	(%)	n	(%)	n	(%)	p-value*
**Do you suffer from a disease of the cardiovascular system?**									0.6007
Yes	12	(7.8)	6	(10.7)	1	(4.2)	19	(8.2)	
No	141	(92.2)	50	(89.3)	23	(95.8)	214	(91.8)	
Missing	1	(0.6)	1	(1.8)	0	(0.0)	2	(0.9)	
**Do you suffer from diabetes?**									0.2965
Yes	5	(3.2)	1	(1.8)	2	(8.7)	8	(3.4)	
No	149	(96.8)	56	(98.2)	21	(91.3)	226	(96.6)	
Missing	0	(0.0)	0	(0.0)	1	(4.2)	1	(0.4)	
**Have you smoked regularly in the past 12 months?**									0.1521
Yes, e-cigarette	5	(3.3)	2	(3.6)	1	(4.2)	8	(3.4)	
Yes, up to 10 cigarettes/ day	18	(11.8)	4	(7.1)	1	(4.2)	23	(9.9)	
Yes, more than 10 cigarettes/ day	26	(17.1)	2	(3.6)	3	(12.5)	31	(13.4)	
No	103	(67.8)	48	(85.7)	19	(79.2)	170	(73.3)	
Missing	2	(1.3)	1	(1.8)	0	(0.0)	3	(1.3)	
**With how many people do you live in the same apartment?**									0.0437
I live alone	33	(21.4)	16	(28.1)	3	(12.5)	52	(22.1)	
With one other person	61	(39.6)	20	(35.1)	4	(16.7)	85	(36.2)	
With 2 - 4 people	55	(35.7)	21	(36.8)	15	(62.5)	91	(38.7)	
With more than 4 people	5	(3.2)	0	(0.0)	2	(8.3)	7	(3.0)	
Missing	0	(0.0)	0	(0.0)	0	(0.0)	0	(0.0)	
**Do children live with you in the apartment?**									0.5350
0	101	(65.6)	38	(66.7)	13	(54.2)	152	(64.7)	
1	28	(18.2)	13	(22.8)	6	(25.0)	47	(20.0)	
2	19	(12.3)	6	(10.5)	3	(12.5)	28	(11.9)	
3	4	(2.6)	0	(0.0)	2	(8.3)	6	(2.6)	
4	2	(1.3)	0	(0.0)	0	(0.0)	2	(0.9)	
Missing	0	(0.0)	0	(0.0)	0	(0.0)	0	(0.0)	

n: Number of non-missing observations; %: Percentages for response categories based on total number of non-missing observations in the respective group, Percentages for missing observations are based on number of all subjects in the respective group; *: Chi-squared test.

Table 15: Epidemiologic baseline characteristics - Subgroup: In 1st test series enrolled

	Train attendants		Train drivers		Maintenance workers		Total		
	n	(%)	n	(%)	n	(%)	n	(%)	p-value*
**Do you suffer from a disease of the cardiovascular system?**									0.0087
Yes	31	(8.0)	17	(10.3)	23	(17.4)	71	(10.3)	
No	358	(92.0)	148	(89.7)	109	(82.6)	615	(89.7)	
Missing	4	(1.0)	2	(1.2)	0	(0.0)	6	(0.9)	
**Do you suffer from diabetes?**									0.2655
Yes	10	(2.6)	7	(4.2)	7	(5.4)	24	(3.5)	
No	381	(97.4)	159	(95.8)	123	(94.6)	663	(96.5)	
Missing	2	(0.5)	1	(0.6)	2	(1.5)	5	(0.7)	
**Have you smoked regularly in the past 12 months?**									<.0001
Yes, e-cigarette	8	(2.1)	3	(1.8)	2	(1.5)	13	(1.9)	
Yes, up to 10 cigarettes/ day	50	(12.9)	9	(5.4)	10	(7.6)	69	(10.1)	
Yes, more than 10 cigarettes/ day	79	(20.4)	12	(7.2)	15	(11.5)	106	(15.5)	
No	250	(64.6)	142	(85.5)	104	(79.4)	496	(72.5)	
Missing	6	(1.5)	1	(0.6)	1	(0.8)	8	(1.2)	
**With how many people do you live in the same apartment?**									0.2226
I live alone	98	(25.0)	41	(24.7)	26	(19.7)	165	(23.9)	
With one other person	156	(39.8)	75	(45.2)	56	(42.4)	287	(41.6)	
With 2 - 4 people	130	(33.2)	50	(30.1)	45	(34.1)	225	(32.6)	
With more than 4 people	8	(2.0)	0	(0.0)	5	(3.8)	13	(1.9)	
Missing	1	(0.3)	1	(0.6)	0	(0.0)	2	(0.3)	
**Do children live with you in the apartment?**									0.3539
0	268	(68.5)	124	(75.2)	90	(68.2)	482	(70.1)	
1	72	(18.4)	31	(18.8)	25	(18.9)	128	(18.6)	
2	43	(11.0)	9	(5.5)	13	(9.8)	65	(9.4)	
3	6	(1.5)	1	(0.6)	4	(3.0)	11	(1.6)	
4	2	(0.5)	0	(0.0)	0	(0.0)	2	(0.3)	
Missing	2	(0.5)	2	(1.2)	0	(0.0)	4	(0.6)	

n: Number of non-missing observations; %: Percentages for response categories based on total number of non-missing observations in the respective group, Percentages for missing observations are based on number of all subjects in the respective group; *: Chi-squared test.

Table 16: Epidemiologic baseline characteristics - Subgroup: In 2nd test series enrolled

	Train attendants		Train drivers		Maintenance workers		Total		
	n	(%)	n	(%)	n	(%)	n	(%)	p-value*
**Do you suffer from a disease of the cardiovascular system?**									0.0614
Yes	4	(4.1)	5	(13.2)	8	(14.3)	17	(8.9)	
No	93	(95.9)	33	(86.8)	48	(85.7)	174	(91.1)	
Missing	1	(1.0)	1	(2.6)	2	(3.4)	4	(2.1)	
**Do you suffer from diabetes?**									0.5643
Yes	1	(1.0)	1	(2.6)	2	(3.5)	4	(2.1)	
No	96	(99.0)	38	(97.4)	55	(96.5)	189	(97.9)	
Missing	1	(1.0)	0	(0.0)	1	(1.7)	2	(1.0)	
**With how many people do you live in the same apartment?**									0.0635
I live alone	21	(21.6)	15	(38.5)	15	(26.3)	51	(26.4)	
With one other person	38	(39.2)	14	(35.9)	16	(28.1)	68	(35.2)	
With 2 - 4 people	33	(34.0)	10	(25.6)	26	(45.6)	69	(35.8)	
With more than 4 people	5	(5.2)	0	(0.0)	0	(0.0)	5	(2.6)	
Missing	1	(1.0)	0	(0.0)	1	(1.7)	2	(1.0)	
**Do children live with you in the apartment?**									0.3256
0	64	(66.0)	32	(82.1)	40	(70.2)	136	(70.5)	
1	19	(19.6)	5	(12.8)	10	(17.5)	34	(17.6)	
2	10	(10.3)	2	(5.1)	7	(12.3)	19	(9.8)	
3	4	(4.1)	0	(0.0)	0	(0.0)	4	(2.1)	
4	0	(0.0)	0	(0.0)	0	(0.0)	0	(0.0)	
Missing	1	(1.0)	0	(0.0)	1	(1.7)	2	(1.0)	

n: Number of non-missing observations; %: Percentages for response categories based on total number of non-missing observations in the respective group, Percentages for missing observations are based on number of all subjects in the respective group; *: Chi-squared test.

Table 17: Epidemiologic baseline characteristics - Subgroup: In 3rd test series enrolled

	Train attendants		Train drivers		Maintenance workers		Total		
	n	(%)	n	(%)	n	(%)	n	(%)	p-value*
**Do you suffer from a disease of the cardiovascular system?**									0.2354
Yes	5	(5.3)	3	(13.6)	3	(13.6)	11	(7.9)	
No	90	(94.7)	19	(86.4)	19	(86.4)	128	(92.1)	
Missing	3	(3.1)	2	(8.3)	6	(21.4)	11	(7.3)	
**Do you suffer from diabetes?**									0.3582
Yes	5	(5.2)	1	(4.5)	3	(13.0)	9	(6.4)	
No	91	(94.8)	21	(95.5)	20	(87.0)	132	(93.6)	
Missing	2	(2.0)	2	(8.3)	5	(17.9)	9	(6.0)	
**Have you smoked regularly in the past 12 months?**									0.3425
Yes, E-cigarette	1	(1.1)	0	(0.0)	1	(4.3)	2	(1.4)	
Yes, up to 10 cigarettes/ day	11	(11.6)	2	(9.1)	3	(13.0)	16	(11.4)	
Yes, more than 10 cigarettes/ day	15	(15.8)	0	(0.0)	2	(8.7)	17	(12.1)	
No	68	(71.6)	20	(90.9)	17	(73.9)	105	(75.0)	
Missing	3	(3.1)	2	(8.3)	5	(17.9)	10	(6.7)	
**With how many people do you live in the same apartment?**									0.8161
I live alone	31	(32.0)	6	(27.3)	6	(26.1)	43	(30.3)	
With one other person	34	(35.1)	8	(36.4)	7	(30.4)	49	(34.5)	
With 2 - 4 people	29	(29.9)	8	(36.4)	10	(43.5)	47	(33.1)	
With more than 4 people	3	(3.1)	0	(0.0)	0	(0.0)	3	(2.1)	
Missing	1	(1.0)	2	(8.3)	5	(17.9)	8	(5.3)	
**Do children live with you in the apartment?**									0.6158
0	71	(74.0)	16	(72.7)	16	(69.6)	103	(73.0)	
1	13	(13.5)	3	(13.6)	6	(26.1)	22	(15.6)	
2	9	(9.4)	3	(13.6)	1	(4.3)	13	(9.2)	
3	3	(3.1)	0	(0.0)	0	(0.0)	3	(2.1)	
4	0	(0.0)	0	(0.0)	0	(0.0)	0	(0.0)	
Missing	2	(2.0)	2	(8.3)	5	(17.9)	9	(6.0)	

n: Number of non-missing observations; %: Percentages for response categories based on total number of non-missing observations in the respective group, Percentages for missing observations are based on number of all subjects in the respective group; *: Chi-squared test.

Table 18: Questionnaire analysis - Total

	Train attendants		Train drivers		Maintenance workers		Total		
	n	(%)	n	(%)	n	(%)	n	(%)	p-value*
**Hours of work in November 2020**									<.0001
n	545		225		204		974		
Missing	44	(7.5)	5	(2.2)	14	(6.4)	63	(6.1)	
Mean value	357.82		383.52		386.73		369.81		
SD	102.71		87.83		80.54		95.98		
Min	0		0		16		0		
Q1	302.0		345.0		358.0		331.0		
Median	377.0		411.0		418.0		396.0		
Q3	446.0		446.0		446.0		446.0		
Max	640		571		501		640		
**With how many different colleagues do you spend more than 15 min per week?**									<.0001
0 - 3	51	(8.8)	88	(38.6)	27	(12.4)	166	(16.2)	
4 - 7	185	(31.9)	95	(41.7)	82	(37.8)	362	(35.3)	
More than 8	344	(59.3)	45	(19.7)	108	(49.8)	497	(48.5)	
Missing	9	(1.5)	2	(0.9)	1	(0.5)	12	(1.2)	
**With how many people do you spend more than 15 min per week in your free time (excluding household members)?**									0.1041
0 - 3	455	(78.2)	172	(74.8)	181	(83.0)	808	(78.4)	
4 - 7	117	(20.1)	56	(24.3)	32	(14.7)	205	(19.9)	
More than 8	10	(1.7)	2	(0.9)	5	(2.3)	17	(1.7)	
Missing	7	(1.2)	0	(0.0)	0	(0.0)	7	(0.7)	

n: Number of non-missing observations; %: Percentages for response categories based on total number of non-missing observations in the respective group, percentages for missing observations are based on number of all subjects in the respective group; SD: Standard deviation; Q1: Lower quartile; Q3: Upper quartile. *: ANOVA (global null hypothesis) for number of working hours in the last 80 days, otherwise chi-squared test; if there were more than 80 days of absence, the working hours were set to missing.

Table 19: Questionnaire analysis - Total (continued)

	Train attendants		Train drivers		Maintenance workers		Total		
	n	(%)	n	(%)	n	(%)	n	(%)	p-value*
**Fever (above 38°C) (Y/N) (currently)**									0.3149
Yes	3	(0.5)	0	(0.0)	0	(0.0)	3	(0.3)	
No	579	(99.5)	229	(100.0)	218	(100.0)	1026	(99.7)	
Missing	7	(1.2)	1	(0.4)	0	(0.0)	8	(0.8)	
**Limb pain (Y/N) (currently)**									0.1130
Yes	10	(1.7)	7	(3.0)	1	(0.5)	18	(1.7)	
No	572	(98.3)	223	(97.0)	217	(99.5)	1012	(98.3)	
Missing	7	(1.2)	0	(0.0)	0	(0.0)	7	(0.7)	
**Sore throat (Y/N) (currently)**									0.0513
Yes	19	(3.3)	4	(1.7)	1	(0.5)	24	(2.3)	
No	563	(96.7)	226	(98.3)	217	(99.5)	1006	(97.7)	
Missing	7	(1.2)	0	(0.0)	0	(0.0)	7	(0.7)	
**Coughing (Y/N) (currently)**									0.1740
Yes	26	(4.5)	4	(1.7)	8	(3.7)	38	(3.7)	
No	553	(95.5)	226	(98.3)	210	(96.3)	989	(96.3)	
Missing	10	(1.7)	0	(0.0)	0	(0.0)	10	(1.0)	
**Rhinitis (Y/N) (currently)**									0.3042
Yes	33	(5.7)	7	(3.1)	11	(5.1)	51	(5.0)	
No	547	(94.3)	221	(96.9)	206	(94.9)	974	(95.0)	
Missing	9	(1.5)	2	(0.9)	1	(0.5)	12	(1.2)	
**Headache (Y/N) (currently)**									0.0489
Yes	35	(6.0)	5	(2.2)	8	(3.7)	48	(4.7)	
No	547	(94.0)	224	(97.8)	210	(96.3)	981	(95.3)	
Missing	7	(1.2)	1	(0.4)	0	(0.0)	8	(0.8)	
**Diarrhea (more than 3 bowel movements daily) (Y/N) (currently)**									0.0941
Yes	7	(1.2)	1	(0.4)	6	(2.8)	14	(1.4)	
No	575	(98.8)	229	(99.6)	212	(97.2)	1016	(98.6)	
Missing	7	(1.2)	0	(0.0)	0	(0.0)	7	(0.7)	
**Smell and taste less than usual (Y/N) (currently)**									0.8065
Yes	3	(0.5)	2	(0.9)	1	(0.5)	6	(0.6)	
No	579	(99.5)	228	(99.1)	217	(99.5)	1024	(99.4)	
Missing	7	(1.2)	0	(0.0)	0	(0.0)	7	(0.7)	

n: Number of non-missing observations; %: Percentages for response categories based on total number of non-missing observations in the respective group, Percentages for missing observations are based on number of all subjects in the respective group; SD: Standard deviation; Q1: Lower quartile; Q3: Upper quartile. *: Chi-squared test

Table 20: Questionnaire analysis - Total (continued)

	Train attendants		Train drivers		Maintenance workers		Total		
	n	(%)	n	(%)	n	(%)	n	(%)	p-value*
**Fever (above 38°C) (Y/N) (since March 2020)**									0.6396
Yes	58	(10.1)	18	(8.0)	20	(9.3)	96	(9.5)	
No	515	(89.9)	208	(92.0)	196	(90.7)	919	(90.5)	
Missing	16	(2.7)	4	(1.7)	2	(0.9)	22	(2.1)	
**Limb pain (Y/N) (since March 2020)**									0.2096
Yes	119	(20.6)	38	(16.8)	34	(15.7)	191	(18.7)	
No	459	(79.4)	188	(83.2)	182	(84.3)	829	(81.3)	
Missing	11	(1.9)	4	(1.7)	2	(0.9)	17	(1.6)	
**Sore throat (Y/N) (since March 2020)**									0.0149
Yes	192	(33.2)	59	(26.1)	51	(23.7)	302	(29.6)	
No	387	(66.8)	167	(73.9)	164	(76.3)	718	(70.4)	
Missing	10	(1.7)	4	(1.7)	3	(1.4)	17	(1.6)	
**Coughing (Y/N) (since March 2020)**									0.1571
Yes	178	(30.8)	60	(26.4)	53	(24.5)	291	(28.5)	
No	399	(69.2)	167	(73.6)	163	(75.5)	729	(71.5)	
Missing	12	(2.0)	3	(1.3)	2	(0.9)	17	(1.6)	
**Rhinitis (Y/N) (since March 2020)**									0.9736
Yes	200	(34.6)	78	(34.1)	73	(33.8)	351	(34.3)	
No	378	(65.4)	151	(65.9)	143	(66.2)	672	(65.7)	
Missing	11	(1.9)	1	(0.4)	2	(0.9)	14	(1.4)	
**Headache (Y/N) (since March 2020)**									<.0001
Yes	259	(44.8)	67	(29.6)	71	(32.9)	397	(38.9)	
No	319	(55.2)	159	(70.4)	145	(67.1)	623	(61.1)	
Missing	11	(1.9)	4	(1.7)	2	(0.9)	17	(1.6)	
**Diarrhea (more than 3 bowel movements daily) (Y/N) (since March 2020)**									0.5551
Yes	63	(11.0)	31	(13.7)	25	(11.7)	119	(11.7)	
No	511	(89.0)	195	(86.3)	189	(88.3)	895	(88.3)	
Missing	15	(2.5)	4	(1.7)	4	(1.8)	23	(2.2)	
**Smell and taste less than usual (Y/N) (since March 2020)**									0.9722
Yes	38	(6.7)	14	(6.2)	14	(6.5)	66	(6.5)	
No	533	(93.3)	212	(93.8)	201	(93.5)	946	(93.5)	
Missing	18	(3.1)	4	(1.7)	3	(1.4)	25	(2.4)	

n: Number of non-missing observations; %: Percentages for response categories based on total number of non-missing observations in the respective group, Percentages for missing observations are based on number of all subjects in the respective group; SD: Standard deviation; Q1: Lower quartile; Q3: Upper quartile. *: Chi-squared test

Table 21: Primary objectives - Total

	Train attendants		Train drivers		Maintenance workers		Total		p-value*
**IgG test (n (%))**									0.1390
Negative	536	(93.2)	219	(96.1)	193	(91.5)	948	(93.5)	
Positive	39	(6.8)	9	(3.9)	18	(8.5)	66	(6.5)	
Missing	14	(2.4)	2	(0.9)	7	(3.2)	23	(2.2)	
**PCR test (n (%))**									0.6252
Negative	586	(99.7)	230	(100.0)	216	(99.5)	1032	(99.7)	
Positive	2	(0.3)	0	(0.0)	1	(0.5)	3	(0.3)	
Missing	1	(0.2)	0	(0.0)	1	(0.5)	2	(0.2)	
**Incidence (n (%))**									0.0092
Negative	401	(94.8)	187	(98.4)	146	(91.3)	734	(95.0)	
Positive	22	(5.2)	3	(1.6)	14	(8.8)	39	(5.0)	
Missing	5	(1.2)	1	(0.5)	4	(2.4)	10	(1.3)	
**Infection rate (n (%))**									0.0744
Negative	487	(91.5)	205	(94.5)	172	(88.2)	864	(91.5)	
Positive	45	(8.5)	12	(5.5)	23	(11.8)	80	(8.5)	
Missing	57	(9.7)	13	(5.7)	23	(10.6)	93	(9.0)	
**Infection rate with additional tests (n (%))**									0.0434
Negative	481	(90.1)	204	(94.0)	170	(86.7)	855	(90.3)	
Positive	53	(9.9)	13	(6.0)	26	(13.3)	92	(9.7)	
Missing	55	(9.3)	13	(5.7)	22	(10.1)	90	(8.7)	
**Incidence (%)**									
Incidence (Estimator + 95%-CI)	5.20	(3.08, 7.32)	1.58	(0.00, 3.35)	8.75	(4.37, 13.13)	5.05	(3.50, 6.59)	
Incidence (Estimator + 90%-CI)	5.20	(3.43, 6.98)	1.58	(0.09, 3.07)	8.75	(5.08, 12.42)	5.05	(3.75, 6.34)	

Only subjects who had no missing values on both the second and third series of tests and tested negative on the second series of tests were included in the calculation of incidence; Infection rate: Participants who had a positive PCR or antibody test since study enrolment.
n: Number of non-missing observations; %: Percentages for response categories based on total number of non-missing observations in the respective group, Percentages for missing observations are based on number of all subjects in the respective group; KI: Confidence interval (asymptomatic), [1]: Exact confidence interval due to employee groups without a positive test result.
*: Chi-squared test; The estimates of the regression are based on a logistic regression with age and gender as covariates; The time-adjusted estimator is based on a Poisson regression with age as covariate and working time as exposure time; The time-adjusted estimator indicates the infection rate in % per 8 h working day.

Table 22: Primary objectives - Total (continued)

	Train attendants		Train drivers		Maintenance workers		Total		p-value*
**Prevalence (%)**									
**Not Adjusted**									
IgG test (Estimator + 95%-CI)	6.78	(4.73, 8.84)	3.95	(1.42, 6.47)	8.53	(4.76, 12.30)	6.51	(4.99, 8.03)	
IgG test (Estimator + 90%-CI)	6.78	(5.06, 8.51)	3.95	(1.83, 6.07)	8.53	(5.37, 11.69)	6.51	(5.23, 7.78)	
PCR test (Estimator + 95%-CI[1])	0.34	(0.04, 1.22)	0.00	(0.00, 1.59)	0.46	(0.01, 2.54)	0.29	(0.06, 0.84)	
PCR test (Estimator + 90%-CI[1])	0.34	(0.06, 1.07)	0.00	(0.00, 1.29)	0.46	(0.02, 2.17)	0.29	(0.08, 0.75)	
Infection rate (Estimator + 95%-CI)	8.46	(6.09, 10.82)	5.53	(2.49, 8.57)	11.79	(7.27, 16.32)	8.47	(6.70, 10.25)	
Infection rate (Estimator + 90%-CI)	8.46	(6.47, 10.44)	5.53	(2.98, 8.08)	11.79	(8.00, 15.59)	8.47	(6.98, 9.97)	
**Adjusted**									
IgG test (Rogan-Gladen Estimator)	6.79		3.77		8.65		6.50		
PCR test (Rogan-Gladen Estimator)	0.34		0.00		0.46		0.29		
Infection rate (Regression + 95%-CI)	8.02	(5.98, 10.69)	5.12	(2.74, 9.36)	10.34	(6.40, 16.29)	-		
Infection rate (Time adjusted + 95%-CI)	0.18	(0.13, 0.24)	0.12	(0.07, 0.22)	0.25	(0.16, 0.38)	-		

Only subjects who had no missing values on both the second and third series of tests and tested negative on the second series of tests were included in the calculation of incidence; Infection rate: Participants who had a positive PCR or antibody test since study enrolment.
n: Number of non-missing observations; %: Percentages for response categories based on total number of non-missing observations in the respective group, Percentages for missing observations are based on number of all subjects in the respective group; KI: Confidence interval (asymptomatic), [1]: Exact confidence interval due to employee groups without a positive test result.
*: Chi-squared test; The estimates of the regression are based on a logistic regression with age and gender as covariates; The time-adjusted estimator is based on a Poisson regression with age as covariate and working time as exposure time; The time-adjusted estimator indicates the infection rate in % per 8 h working day.

Table 23: Primary objectives – Subgroup: Age under 45

	Train attendants		Train drivers		Maintenance workers		Total		p-value*
**IgG test (n (%))**									0.3040
Negative	262	(92.6)	66	(95.7)	73	(89.0)	401	(92.4)	
Positive	21	(7.4)	3	(4.3)	9	(11.0)	33	(7.6)	
Missing	8	(2.7)	0	(0.0)	4	(4.7)	12	(2.7)	
**PCR test (n (%))**									0.5065
Negative	289	(99.7)	69	(100.0)	85	(98.8)	443	(99.6)	
Positive	1	(0.3)	0	(0.0)	1	(1.2)	2	(0.4)	
Missing	1	(0.3)	0	(0.0)	0	(0.0)	1	(0.2)	
**Incidence (n (%))**									0.0379
Negative	189	(93.1)	55	(100.0)	52	(88.1)	296	(93.4)	
Positive	14	(6.9)	0	(0.0)	7	(11.9)	21	(6.6)	
Missing	3	(1.5)	0	(0.0)	2	(3.3)	5	(1.6)	
**Infection rate (n (%))**									0.0592
Negative	235	(90.7)	60	(93.8)	61	(82.4)	356	(89.7)	
Positive	24	(9.3)	4	(6.3)	13	(17.6)	41	(10.3)	
Missing	32	(11.0)	5	(7.2)	12	(14.0)	49	(11.0)	
**Incidence (%)**									
Incidence (Estimator + 95%-CI[1])	6.90	(3.82, 11.30)	0.00	(0.00, 6.49)	11.86	(4.91, 22.93)	6.62	(4.15, 9.95)	
Incidence (Estimator + 90%-CI[1])	6.90	(4.22, 10.57)	0.00	(0.00, 5.30)	11.86	(5.70, 21.13)	6.62	(4.48, 9.40)	
**Prevalence (%)**									
**Not Adjusted**									
IgG test(Estimator + 95%-CI)	7.42	(4.37, 10.47)	4.35	(0.00, 9.16)	10.98	(4.21, 17.74)	7.60	(5.11, 10.10)	
IgG test(Estimator + 90%-CI)	7.42	(4.86, 9.98)	4.35	(0.31, 8.39)	10.98	(5.30, 16.65)	7.60	(5.51, 9.70)	
PCR test(Estimator + 95%-CI[1])	0.34	(0.01, 1.91)	0.00	(0.00, 5.21)	1.16	(0.03, 6.31)	0.45	(0.05, 1.61)	
PCR test(Estimator + 90%-CI[1])	0.34	(0.02, 1.63)	0.00	(0.00, 4.25)	1.16	(0.06, 5.40)	0.45	(0.08, 1.41)	
Infection rate (Estimator + 95%-CI)	9.27	(5.74, 12.80)	6.25	(0.32, 12.18)	17.57	(8.90, 26.24)	10.33	(7.33, 13.32)	
Infection rate (Estimator + 90%-CI)	9.27	(6.30, 12.23)	6.25	(1.27, 11.23)	17.57	(10.29, 24.84)	10.33	(7.82, 12.84)	
**Adjusted**									
Infection rate (Regression + 95%-CI)	9.27	(6.27, 13.51)	4.69	(1.61, 12.87)	13.28	(6.89, 24.04)	-		
Infection rate (Time adjusted + 95%-CI)	0.21	(0.14, 0.33)	0.12	(0.05, 0.33)	0.36	(0.21, 0.62)	-		

Only subjects who had no missing values on both the second and third series of tests and tested negative on the second series of tests were included in the calculation of incidence; Infection rate: participants who had a positive PCR or antibody test since 1st test series.
n: Number of non-missing observations; %: Percentages for response categories based on total number of non-missing observations in the respective group, Percentages for missing observations are based on number of all subjects in the respective group; KI: Confidence interval (asymptomatic).
[1]: Exact confidence interval due to employee groups without a positive test result.
*: Chi-squared test; The estimates of the regression are based on a logistic regression with age and gender as covariates; The time-adjusted estimator is based on a Poisson regression with age as covariate and working time as exposure time; The time-adjusted estimators indicate the infection rate per 8 h working day.

Table 24: Primary objectives - Subgroup: Age 45 or more

	Train attendants		Train drivers		Maintenance workers		Total		p-value*
**IgG test (n (%))**									0.4572
Negative	270	(93.8)	147	(96.1)	115	(92.7)	532	(94.2)	
Positive	18	(6.3)	6	(3.9)	9	(7.3)	33	(5.8)	
Missing	6	(2.0)	1	(0.6)	3	(2.4)	10	(1.7)	
**PCR test (n (%))**									0.6206
Negative	293	(99.7)	154	(100.0)	126	(100.0)	573	(99.8)	
Positive	1	(0.3)	0	(0.0)	0	(0.0)	1	(0.2)	
Missing	0	(0.0)	0	(0.0)	1	(0.8)	1	(0.2)	
**Incidence (n (%))**									0.1916
Negative	209	(96.3)	128	(97.7)	94	(93.1)	431	(96.0)	
Positive	8	(3.7)	3	(2.3)	7	(6.9)	18	(4.0)	
Missing	2	(0.9)	1	(0.8)	2	(1.9)	5	(1.1)	
**Infection rate (n (%))**									0.5639
Negative	248	(92.2)	139	(94.6)	106	(91.4)	493	(92.7)	
Positive	21	(7.8)	8	(5.4)	10	(8.6)	39	(7.3)	
Missing	25	(8.5)	7	(4.5)	11	(8.7)	43	(7.5)	
**Incidence (%)**									
Incidence (Estimator + 95%-CI)	3.69	(1.18, 6.19)	2.29	(0.00, 4.85)	6.93	(1.98, 11.88)	4.01	(2.19, 5.82)	
Incidence (Estimator + 90%-CI)	3.69	(1.58, 5.79)	2.29	(0.14, 4.44)	6.93	(2.77, 11.09)	4.01	(2.49, 5.53)	
**Prevalence (%)**									
**Not Adjusted**									
IgG test(Estimator + 95%-CI)	6.25	(3.45, 9.05)	3.92	(0.85, 7.00)	7.26	(2.69, 11.82)	5.84	(3.91, 7.77)	
IgG test(Estimator + 90%-CI)	6.25	(3.90, 8.60)	3.92	(1.34, 6.50)	7.26	(3.43, 11.09)	5.84	(4.22, 7.46)	
PCR test(Estimator + 95%-CI[1])	0.34	(0.01, 1.88)	0.00	(0.00, 2.37)	0.00	(0.00, 2.89)	0.17	(0.00, 0.97)	
PCR test(Estimator + 90%-CI[1])	0.34	(0.02, 1.60)	0.00	(0.00, 1.93)	0.00	(0.00, 2.35)	0.17	(0.01, 0.82)	
Infection rate (Estimator + 95%-CI)	7.81	(4.60, 11.01)	5.44	(1.78, 9.11)	8.62	(3.51, 13.73)	7.33	(5.12, 9.55)	
Infection rate (Estimator + 90%-CI)	7.81	(5.12, 10.50)	5.44	(2.36, 8.52)	8.62	(4.33, 12.91)	7.33	(5.47, 9.19)	
**Adjusted**									
Infection rate (Regression + 95%-CI)	6.88	(4.37, 10.67)	5.31	(2.41, 11.32)	8.38	(4.08, 16.44)	-		
Infection rate (Time adjusted + 95%-CI)	0.15	(0.09, 0.24)	0.12	(0.06, 0.23)	0.18	(0.09, 0.34)	-		

Only subjects who had no missing values on both the second and third series of tests and tested negative on the second series of tests were included in the calculation of incidence; Infection rate: participants who had a positive PCR or antibody test since 1st test series.
n: Number of non-missing observations; %: Percentages for response categori.es based on total number of non-missing observations in the respective group, Percentages for missing observations are based on number of all subjects in the respective group; KI: Confidence interval (asymptomatic).
[1]: Exact confidence interval due to employee groups without a positive test result.
*: Chi-squared test; The estimates of the regression are based on a logistic regression with age and gender as covariates; The time-adjusted estimator is based on a Poisson regression with age as covariate and working time as exposure time; The time-adjusted estimators indicate the infection rate per 8 h working day.

Table 25: Primary objectives - Subgroup: Berlin

	Train attendants		Train drivers		Maintenance workers		Total		p-value*
**IgG test (n (%))**									0.0889
Negative	144	(92.3)	59	(96.7)	48	(85.7)	251	(91.9)	
Positive	12	(7.7)	2	(3.3)	8	(14.3)	22	(8.1)	
Missing	1	(0.6)	1	(1.6)	1	(1.8)	3	(1.1)	
**PCR test (n (%))**									0.6858
Negative	156	(99.4)	62	(100.0)	56	(100.0)	274	(99.6)	
Positive	1	(0.6)	0	(0.0)	0	(0.0)	1	(0.4)	
Missing	0	(0.0)	0	(0.0)	1	(1.8)	1	(0.4)	
**Incidence (n (%))**									0.2282
Negative	105	(92.9)	50	(98.0)	43	(89.6)	198	(93.4)	
Positive	8	(7.1)	1	(2.0)	5	(10.4)	14	(6.6)	
Missing	0	(0.0)	0	(0.0)	1	(2.0)	1	(0.5)	
**Infection rate (n (%))**									0.1161
Negative	131	(90.3)	56	(96.6)	46	(85.2)	233	(90.7)	
Positive	14	(9.7)	2	(3.4)	8	(14.8)	24	(9.3)	
Missing	12	(7.6)	4	(6.5)	3	(5.3)	19	(6.9)	
**Incidence (%)**									
Incidence (Estimator + 95%-CI)	7.08	(2.35, 11.81)	1.96	(0.00, 5.77)	10.42	(1.77, 19.06)	6.60	(3.26, 9.95)	
Incidence (Estimator + 90%-CI)	7.08	(3.11, 11.05)	1.96	(0.00, 5.15)	10.42	(3.16, 17.67)	6.60	(3.80, 9.41)	
**Prevalence (%)**									
**Not Adjusted**									
IgG test(Estimator + 95%-CI)	7.69	(3.51, 11.87)	3.28	(0.00, 7.75)	14.29	(5.12, 23.45)	8.06	(4.83, 11.29)	
IgG test(Estimator + 90%-CI)	7.69	(4.18, 11.20)	3.28	(0.00, 7.03)	14.29	(6.59, 21.98)	8.06	(5.35, 10.77)	
PCR test(Estimator + 95%-CI[1])	0.64	(0.02, 3.50)	0.00	(0.00, 5.78)	0.00	(0.00, 6.38)	0.36	(0.01, 2.01)	
PCR test(Estimator + 90%-CI[1])	0.64	(0.03, 2.99)	0.00	(0.00, 4.72)	0.00	(0.00, 5.21)	0.36	(0.02, 1.71)	
Infection rate (Estimator + 95%-CI)	9.66	(4.85, 14.46)	3.45	(0.00, 8.14)	14.81	(5.34, 24.29)	9.34	(5.78, 12.90)	
Infection rate (Estimator + 90%-CI)	9.66	(5.62, 13.69)	3.45	(0.00, 7.39)	14.81	(6.86, 22.77)	9.34	(6.35, 12.32)	
**Adjusted**									
Infection rate (Regression + 95%-CI)	9.62	(5.76, 15.63)	4.04	(0.93, 15.85)	16.42	(7.34, 32.76)	-		
Infection rate (Time adjusted + 95%-CI)	0.22	(0.13, 0.38)	0.08	(0.02, 0.30)	0.30	(0.14, 0.63)	-		

Only subjects who had no missing values on both the second and third series of tests and tested negative on the second series of tests were included in the calculation of incidence; Infection rate: participants who had a positive PCR or antibody test since 1st test series.
n: Number of non-missing observations; %: Percentages for response categories based on total number of non-missing observations in the respective group, Percentages for missing observations are based on number of all subjects in the respective group; KI: Confidence interval (asymptomatic).
[1]: Exact confidence interval due to employee groups without a positive test result.
*: Chi-squared test; The estimates of the regression are based on a logistic regression with age and gender as covariates; The time-adjusted estimator is based on a Poisson regression with age as covariate and working time as exposure time; The time-adjusted estimators indicate the infection rate per 8 h working day.

Table 26: Primary objectives - Subgroup: Hamburg

	Train attendants		Train drivers		Maintenance workers		Total		p-value*
**IgG test (n (%))**									0.1474
Negative	148	(99.3)	54	(96.4)	87	(95.6)	289	(97.6)	
Positive	1	(0.7)	2	(3.6)	4	(4.4)	7	(2.4)	
Missing	2	(1.3)	0	(0.0)	1	(1.1)	3	(1.0)	
**PCR test (n (%))**									0.3234
Negative	151	(100.0)	56	(100.0)	91	(98.9)	298	(99.7)	
Positive	0	(0.0)	0	(0.0)	1	(1.1)	1	(0.3)	
Missing	0	(0.0)	0	(0.0)	0	(0.0)	0	(0.0)	
**Incidence (n (%))**									0.0278
Negative	106	(100.0)	49	(98.0)	59	(93.7)	214	(97.7)	
Positive	0	(0.0)	1	(2.0)	4	(6.3)	5	(2.3)	
Missing	1	(0.9)	0	(0.0)	0	(0.0)	1	(0.5)	
**Infection rate (n (%))**									0.0120
Negative	141	(99.3)	50	(94.3)	75	(91.5)	266	(96.0)	
Positive	1	(0.7)	3	(5.7)	7	(8.5)	11	(4.0)	
Missing	9	(6.0)	3	(5.4)	10	(10.9)	22	(7.4)	
**Incidence (%)**									
Incidence (Estimator + 95%-CI[1])	0.00	(0.00, 3.42)	2.00	(0.05, 10.65)	6.35	(1.76, 15.47)	2.28	(0.75, 5.25)	
Incidence (Estimator + 90%-CI[1])	0.00	(0.00, 2.79)	2.00	(0.10, 9.14)	6.35	(2.20, 13.94)	2.28	(0.90, 4.74)	
**Prevalence (%)**									
**Not Adjusted**									
IgG test(Estimator + 95%-CI)	0.67	(0.00, 1.98)	3.57	(0.00, 8.43)	4.40	(0.18, 8.61)	2.36	(0.63, 4.10)	
IgG test(Estimator + 90%-CI)	0.67	(0.00, 1.77)	3.57	(0.00, 7.65)	4.40	(0.86, 7.93)	2.36	(0.91, 3.82)	
PCR test(Estimator + 95%-CI[1])	0.00	(0.00, 2.41)	0.00	(0.00, 6.38)	1.09	(0.03, 5.91)	0.33	(0.01, 1.85)	
PCR test(Estimator + 90%-CI[1])	0.00	(0.00, 1.96)	0.00	(0.00, 5.21)	1.09	(0.06, 5.05)	0.33	(0.02, 1.58)	
Infection rate (Estimator + 95%-CI)	0.70	(0.00, 2.08)	5.66	(0.00, 11.88)	8.54	(2.49, 14.58)	3.97	(1.67, 6.27)	
Infection rate (Estimator + 90%-CI)	0.70	(0.00, 1.86)	5.66	(0.44, 10.88)	8.54	(3.46, 13.61)	3.97	(2.04, 5.90)	
**Adjusted**									
Infection rate (Regression + 95%-CI)	0.56	(0.07, 4.02)	5.52	(1.14, 22.80)	6.76	(1.86, 21.77)	-		
Infection rate (Time adjusted + 95%-CI)	0.01	(0.00, 0.10)	0.13	(0.04, 0.42)	0.16	(0.07, 0.36)	-		

Only subjects who had no missing values on both the second and third series of tests and tested negative on the second series of tests were included in the calculation of incidence; Infection rate: participants who had a positive PCR or antibody test since 1st test series.
n: Number of non-missing observations; %: Percentages for response categories based on total number of non-missing observations in the respective group, Percentages for missing observations are based on number of all subjects in the respective group; KI: Confidence interval (asymptomatic).
[1]: Exact confidence interval due to employee groups without a positive test result.
*: Chi-squared test; The estimates of the regression are based on a logistic regression with age and gender as covariates; The time-adjusted estimator is based on a Poisson regression with age as covariate and working time as exposure time; The time-adjusted estimators indicate the infection rate per 8 h working day.

Table 27: Primary objectives - Subgroup: Munich

	Train attendants		Train drivers		Maintenance workers		Total		p-value*
**IgG test (n (%))**									0.3885
Negative	112	(88.9)	51	(92.7)	41	(95.3)	204	(91.1)	
Positive	14	(11.1)	4	(7.3)	2	(4.7)	20	(8.9)	
Missing	1	(0.8)	0	(0.0)	2	(4.4)	3	(1.3)	
**PCR test (n (%))**									0.6713
Negative	125	(99.2)	55	(100.0)	45	(100.0)	225	(99.6)	
Positive	1	(0.8)	0	(0.0)	0	(0.0)	1	(0.4)	
Missing	1	(0.8)	0	(0.0)	0	(0.0)	1	(0.4)	
**Incidence (n (%))**									0.1661
Negative	86	(92.5)	40	(100.0)	29	(96.7)	155	(95.1)	
Positive	7	(7.5)	0	(0.0)	1	(3.3)	8	(4.9)	
Missing	1	(1.1)	0	(0.0)	1	(3.2)	2	(1.2)	
**Infection rate (n (%))**									0.5060
Negative	102	(87.2)	47	(92.2)	36	(92.3)	185	(89.4)	
Positive	15	(12.8)	4	(7.8)	3	(7.7)	22	(10.6)	
Missing	10	(7.9)	4	(7.3)	6	(13.3)	20	(8.8)	
**Incidence (%)**									
Incidence (Estimator + 95%-CI[1])	7.53	(3.08, 14.90)	0.00	(0.00, 8.81)	3.33	(0.08, 17.22)	4.91	(2.14, 9.44)	
Incidence (Estimator + 90%-CI[1])	7.53	(3.59, 13.67)	0.00	(0.00, 7.22)	3.33	(0.17, 14.86)	4.91	(2.47, 8.68)	
**Prevalence (%)**									
**Not Adjusted**									
IgG test(Estimator + 95%-CI)	11.11	(5.62, 16.60)	7.27	(0.41, 14.14)	4.65	(0.00, 10.95)	8.93	(5.19, 12.66)	
IgG test(Estimator + 90%-CI)	11.11	(6.51, 15.72)	7.27	(1.51, 13.03)	4.65	(0.00, 9.93)	8.93	(5.79, 12.06)	
PCR test(Estimator + 95%-CI[1])	0.79	(0.02, 4.34)	0.00	(0.00, 6.49)	0.00	(0.00, 7.87)	0.44	(0.01, 2.44)	
PCR test(Estimator + 90%-CI[1])	0.79	(0.04, 3.71)	0.00	(0.00, 5.30)	0.00	(0.00, 6.44)	0.44	(0.02, 2.08)	
Infection rate (Estimator + 95%-CI)	12.82	(6.76, 18.88)	7.84	(0.46, 15.22)	7.69	(0.00, 16.06)	10.63	(6.43, 14.83)	
Infection rate (Estimator + 90%-CI)	12.82	(7.74, 17.90)	7.84	(1.65, 14.04)	7.69	(0.67, 14.71)	10.63	(7.10, 14.15)	
**Adjusted**									
Infection rate (Regression + 95%-CI)	12.24	(7.31, 19.79)	6.14	(2.03, 17.11)	4.59	(1.04, 18.02)	-		
Infection rate (Time adjusted + 95%-CI)	0.29	(0.17, 0.50)	0.18	(0.07, 0.49)	0.21	(0.07, 0.66)	-		

Only subjects who had no missing values on both the second and third series of tests and tested negative on the second series of tests were included in the calculation of incidence; Infection rate: participants who had a positive PCR or antibody test since 1st test series.
n: Number of non-missing observations; %: Percentages for response categories based on total number of non-missing observations in the respective group, Percentages for missing observations are based on number of all subjects in the respective group; KI: Confidence interval (asymptomatic).
[1]: Exact confidence interval due to employee groups without a positive test result.
*: Chi-squared test; The estimates of the regression are based on a logistic regression with age and gender as covariates; The time-adjusted estimator is based on a Poisson regression with age as covariate and working time as exposure time; The time-adjusted estimators indicate the infection rate per 8 h working day.

Table 28: Primary objectives - Subgroup: Frankfurt am Main

	Train attendants		Train drivers		Maintenance workers		Total		p-value*
**IgG test (n (%))**									0.0360
Negative	132	(91.7)	55	(98.2)	17	(81.0)	204	(92.3)	
Positive	12	(8.3)	1	(1.8)	4	(19.0)	17	(7.7)	
Missing	10	(6.5)	1	(1.8)	3	(12.5)	14	(6.0)	
**PCR test (n (%))**									N/A
Negative	154	(100.0)	57	(100.0)	24	(100.0)	235	(100.0)	
Positive	0	(0.0)	0	(0.0)	0	(0.0)	0	(0.0)	
Missing	0	(0.0)	0	(0.0)	0	(0.0)	0	(0.0)	
**Incidence (n (%))**									0.0184
Negative	104	(93.7)	48	(98.0)	15	(78.9)	167	(93.3)	
Positive	7	(6.3)	1	(2.0)	4	(21.1)	12	(6.7)	
Missing	3	(2.6)	1	(2.0)	2	(9.5)	6	(3.2)	
**Infection rate (n (%))**									0.0599
Negative	113	(88.3)	52	(94.5)	15	(75.0)	180	(88.7)	
Positive	15	(11.7)	3	(5.5)	5	(25.0)	23	(11.3)	
Missing	26	(16.9)	2	(3.5)	4	(16.7)	32	(13.6)	
**Incidence (%)**									
Incidence (Estimator + 95%-CI)	6.31	(1.78, 10.83)	2.04	(0.00, 6.00)	21.05	(2.72, 39.38)	6.70	(3.04, 10.37)	
Incidence (Estimator + 90%-CI)	6.31	(2.51, 10.10)	2.04	(0.00, 5.36)	21.05	(5.67, 36.44)	6.70	(3.63, 9.78)	
**Prevalence (%)**									
**Not Adjusted**									
IgG test(Estimator + 95%-CI)	8.33	(3.82, 12.85)	1.79	(0.00, 5.25)	19.05	(2.25, 35.84)	7.69	(4.18, 11.21)	
IgG test(Estimator + 90%-CI)	8.33	(4.54, 12.12)	1.79	(0.00, 4.70)	19.05	(4.95, 33.14)	7.69	(4.74, 10.64)	
PCR test(Estimator + 95%-CI[1])	0.00	(0.00, 2.37)	0.00	(0.00, 6.27)	0.00	(0.00, 14.25)	0.00	(0.00, 1.56)	
PCR test(Estimator + 90%-CI[1])	0.00	(0.00, 1.93)	0.00	(0.00, 5.12)	0.00	(0.00, 11.73)	0.00	(0.00, 1.27)	
Infection rate (Estimator + 95%-CI)	11.72	(6.15, 17.29)	5.45	(0.00, 11.46)	25.00	(6.02, 43.98)	11.33	(6.97, 15.69)	
Infection rate (Estimator + 90%-CI)	11.72	(7.04, 16.39)	5.45	(0.42, 10.49)	25.00	(9.07, 40.93)	11.33	(7.67, 14.99)	
**Adjusted**									
Infection rate (Regression + 95%-CI)	11.19	(6.68, 18.17)	4.97	(1.40, 16.12)	23.49	(8.75, 49.56)	-		
Infection rate (Time adjusted + 95%-CI)	0.24	(0.14, 0.42)	0.11	(0.03, 0.34)	0.54	(0.22, 1.32)	-		

Only subjects who had no missing values on both the second and third series of tests and tested negative on the second series of tests were included in the calculation of incidence; Infection rate: participants who had a positive PCR or antibody test since 1st test series.
n: Number of non-missing observations; %: Percentages for response categories based on total number of non-missing observations in the respective group, Percentages for missing observations are based on number of all subjects in the respective group; KI: Confidence interval (asymptomatic).
[1]: Exact confidence interval due to employee groups without a positive test result.
*: Chi-squared test; The estimates of the regression are based on a logistic regression with age and gender as covariates; The time-adjusted estimator is based on a Poisson regression with age as covariate and working time as exposure time; The time-adjusted estimators indicate the infection rate per 8 h working day.

Table 29: Primary objectives - Subgroup: In 1st test series enrolled

	Train attendants		Train drivers		Maintenance workers		Total		p-value*
**IgG test (n (%))**									0.0782
Negative	359	(93.5)	160	(96.4)	115	(89.8)	634	(93.5)	
Positive	25	(6.5)	6	(3.6)	13	(10.2)	44	(6.5)	
Missing	9	(2.3)	1	(0.6)	4	(3.0)	14	(2.0)	
**PCR test (n (%))**									0.5748
Negative	390	(99.5)	167	(100.0)	130	(99.2)	687	(99.6)	
Positive	2	(0.5)	0	(0.0)	1	(0.8)	3	(0.4)	
Missing	1	(0.3)	0	(0.0)	1	(0.8)	2	(0.3)	
**Incidence (n (%))**									0.0236
Negative	314	(95.2)	148	(98.0)	95	(90.5)	557	(95.1)	
Positive	16	(4.8)	3	(2.0)	10	(9.5)	29	(4.9)	
Missing	5	(1.5)	1	(0.7)	2	(1.9)	8	(1.3)	
**Infection rate (n (%))**									0.0306
Negative	311	(90.9)	146	(94.2)	94	(84.7)	551	(90.6)	
Positive	31	(9.1)	9	(5.8)	17	(15.3)	57	(9.4)	
Missing	51	(13.0)	12	(7.2)	21	(15.9)	84	(12.1)	
**Incidence (%)**									
Incidence (Estimator + 95%-CI)	4.85	(2.53, 7.17)	1.99	(0.00, 4.21)	9.52	(3.91, 15.14)	4.95	(3.19, 6.70)	
Incidence (Estimator + 90%-CI)	4.85	(2.90, 6.79)	1.99	(0.12, 3.85)	9.52	(4.81, 14.24)	4.95	(3.48, 6.42)	
**Prevalence (%)**									
**Not Adjusted**									
IgG test(Estimator + 95%-CI)	6.51	(4.04, 8.98)	3.61	(0.78, 6.45)	10.16	(4.92, 15.39)	6.49	(4.64, 8.34)	
IgG test(Estimator + 90%-CI)	6.51	(4.44, 8.58)	3.61	(1.23, 6.00)	10.16	(5.76, 14.55)	6.49	(4.93, 8.05)	
PCR test (Estimator + 95%-CI[1])	0.51	(0.06, 1.83)	0.00	(0.00, 2.18)	0.76	(0.02, 4.18)	0.43	(0.09, 1.27)	
PCR test (Estimator + 90%-CI[1])	0.51	(0.09, 1.60)	0.00	(0.00, 1.78)	0.76	(0.04, 3.57)	0.43	(0.12, 1.12)	
Infection rate (Estimator + 95%-CI)	9.06	(6.02, 12.11)	5.81	(2.12, 9.49)	15.32	(8.62, 22.01)	9.38	(7.06, 11.69)	
Infection rate (Estimator + 90%-CI)	9.06	(6.51, 11.62)	5.81	(2.72, 8.90)	15.32	(9.69, 20.94)	9.38	(7.43, 11.32)	
**Adjusted**									
Infection rate (Regression + 95%-CI)	8.49	(5.94, 12.00)	6.07	(2.95, 12.08)	15.48	(9.11, 25.07)	-		
Infection rate (Time adjusted + 95%-CI)	0.18	(0.12, 0.27)	0.13	(0.07, 0.25)	0.32	(0.20, 0.53)	-		

Only subjects who had no missing values on both the second and third series of tests and tested negative on the second series of tests were included in the calculation of incidence; Infection rate: participants who had a positive PCR or antibody test since 1st test series.
n: Number of non-missing observations; %: Percentages for response categories based on total number of non-missing observations in the respective group, Percentages for missing observations are based on number of all subjects in the respective group; KI: Confidence interval (asymptomatic)
[1]: Exact confidence interval due to employee groups without a positive test result
*: Chi-squared test; The estimates of the regression are based on a logistic regression with age and gender as covariates; The time-adjusted estimator is based on a Poisson regression with age as covariate and working time as exposure time; The time-adjusted estimators indicate the infection rate per 8 h working day.

Table 30: Primary objectives - Subgroup: In 2nd test series enrolled

	Train attendants		Train drivers		Maintenance workers		Total		p-value*
**IgG test (n (%))**									0.1167
Negative	88	(89.8)	39	(100.0)	51	(92.7)	178	(92.7)	
Positive	10	(10.2)	0	(0.0)	4	(7.3)	14	(7.3)	
Missing	0	(0.0)	0	(0.0)	3	(5.2)	3	(1.5)	
**PCR test (n (%))**									N/A
Negative	98	(100.0)	39	(100.0)	58	(100.0)	195	(100.0)	
Positive	0	(0.0)	0	(0.0)	0	(0.0)	0	(0.0)	
Missing	0	(0.0)	0	(0.0)	0	(0.0)	0	(0.0)	
**Incidence (n (%))**									0.2429
Negative	87	(93.5)	39	(100.0)	51	(92.7)	177	(94.7)	
Positive	6	(6.5)	0	(0.0)	4	(7.3)	10	(5.3)	
Missing	0	(0.0)	0	(0.0)	2	(3.5)	2	(1.1)	
**Infection rate (n (%))**									0.1199
Negative	87	(89.7)	39	(100.0)	51	(91.1)	177	(92.2)	
Positive	10	(10.3)	0	(0.0)	5	(8.9)	15	(7.8)	
Missing	1	(1.0)	0	(0.0)	2	(3.4)	3	(1.5)	
**Incidence (%)**									
Incidence (Estimator + 95%-CI[1])	6.45	(2.40, 13.52)	0.00	(0.00, 9.03)	7.27	(2.02, 17.59)	5.35	(2.59, 9.61)	
Incidence (Estimator + 90%-CI[1])	6.45	(2.85, 12.34)	0.00	(0.00, 7.39)	7.27	(2.52, 15.87)	5.35	(2.93, 8.90)	
**Prevalence (%)**									
**Not Adjusted**									
IgG test(Estimator + 95%-CI[1])	10.20	(5.00, 17.97)	0.00	(0.00, 9.03)	7.27	(2.02, 17.59)	7.29	(4.04, 11.93)	
IgG test(Estimator + 90%-CI[1])	10.20	(5.64, 16.69)	0.00	(0.00, 7.39)	7.27	(2.52, 15.87)	7.29	(4.46, 11.16)	
PCR test (Estimator + 95%-CI[1])	0.00	(0.00, 3.69)	0.00	(0.00, 9.03)	0.00	(0.00, 6.16)	0.00	(0.00, 1.87)	
PCR test (Estimator + 90%-CI[1])	0.00	(0.00, 3.01)	0.00	(0.00, 7.39)	0.00	(0.00, 5.03)	0.00	(0.00, 1.52)	
Infection rate (Estimator + 95%-CI[1])	10.31	(5.06, 18.14)	0.00	(0.00, 9.03)	8.93	(2.96, 19.62)	7.81	(4.44, 12.56)	
Infection rate (Estimator + 90%-CI[1])	10.31	(5.70, 16.86)	0.00	(0.00, 7.39)	8.93	(3.58, 17.86)	7.81	(4.88, 11.78)	
**Adjusted**									
Infection rate (Regression + 95%-CI)	7.51	(3.37, 15.93)	0.00	(0.00, 100.0)	3.25	(0.91, 10.88)	-		
Infection rate (Time adjusted + 95%-CI)	0.22	(0.11, 0.42)	0.00	(0.00, 0.0)	0.17	(0.07, 0.43)	-		

Only subjects who had no missing values on both the second and third series of tests and tested negative on the second series of tests were included in the calculation of incidence; Infection rate: participants who had a positive PCR or antibody test since 1st test series.
n: Number of non-missing observations; %: Percentages for response categories based on total number of non-missing observations in the respective group, Percentages for missing observations are based on number of all subjects in the respective group; KI: Confidence interval (asymptomatic)
[1]: Exact confidence interval due to employee groups without a positive test result
*: Chi-squared test; The estimates of the regression are based on a logistic regression with age and gender as covariates; The time-adjusted estimator is based on a Poisson regression with age as covariate and working time as exposure time; The time-adjusted estimators indicate the infection rate per 8 h working day.

Table 31: Primary objectives - Subgroup: In 3rd test series enrolled

	Train attendants		Train drivers		Maintenance workers		Total		p-value*
**IgG test (n (%))**									0.2291
Negative	89	(95.7)	20	(87.0)	27	(96.4)	136	(94.4)	
Positive	4	(4.3)	3	(13.0)	1	(3.6)	8	(5.6)	
Missing	5	(5.1)	1	(4.2)	0	(0.0)	6	(4.0)	
**PCR test (n (%))**									N/A
Negative	98	(100.0)	24	(100.0)	28	(100.0)	150	(100.0)	
Positive	0	(0.0)	0	(0.0)	0	(0.0)	0	(0.0)	
Missing	0	(0.0)	0	(0.0)	0	(0.0)	0	(0.0)	
**Infection rate (n (%))**									0.2291
Negative	89	(95.7)	20	(87.0)	27	(96.4)	136	(94.4)	
Positive	4	(4.3)	3	(13.0)	1	(3.6)	8	(5.6)	
Missing	5	(5.1)	1	(4.2)	0	(0.0)	6	(4.0)	
**Prevalence (%)**									
**Not Adjusted**									
IgG test (Estimator + 95%-CI)	4.30	(0.18, 8.42)	13.04	(0.00, 26.81)	3.57	(0.00, 10.45)	5.56	(1.81, 9.30)	
IgG test (Estimator + 90%-CI)	4.30	(0.84, 7.76)	13.04	(1.49, 24.59)	3.57	(0.00, 9.34)	5.56	(2.42, 8.70)	
PCR test (Estimator + 95%-CI[1])	0.00	(0.00, 3.69)	0.00	(0.00, 14.25)	0.00	(0.00, 12.34)	0.00	(0.00, 2.43)	
PCR test (Estimator + 90%-CI[1])	0.00	(0.00, 3.01)	0.00	(0.00, 11.73)	0.00	(0.00, 10.15)	0.00	(0.00, 1.98)	
Infection rate (Estimator + 95%-CI)	4.30	(0.18, 8.42)	13.04	(0.00, 26.81)	3.57	(0.00, 10.45)	5.56	(1.81, 9.30)	
Infection rate (Estimator + 90%-CI)	4.30	(0.84, 7.76)	13.04	(1.49, 24.59)	3.57	(0.00, 9.34)	5.56	(2.42, 8.70)	

[1]: Exact confidence interval due to employee groups without a positive test result
*: Chi-squared test; The estimates of the regression are based on a logistic regression with age and gender as covariates; The time-adjusted estimator is based on a Poisson regression with age as covariate and working time as exposure time; The time-adjusted estimators indicate the infection rate per 8 h working day.

Table 32: Course of SARS-CoV-2 infections

	Train attendants		Train drivers		Maintenance workers		Total		
	n	(%)	n	(%)	n	(%)	n	(%)	p-value*
**SARS-CoV-2 course (IgG test)**									0.2004
Symptomatic	28	(73.7)	9	(100.0)	14	(82.4)	51	(79.7)	
Asymptomatic	10	(26.3)	0	(0.0)	3	(17.6)	13	(20.3)	
Missing	1	(2.6)	0	(0.0)	1	(5.6)	2	(3.0)	
**SARS-CoV-2 course (PCR test)**									N/A
Asymptomatic	2	(100.0)	0	(0.0)	1	(100.0)	3	(100.0)	
Symptomatic	0	(0.0)	0	(0.0)	0	(0.0)	0	(0.0)	
Missing	0	(0.0)	0	(0.0)	0	(0.0)	0	(0.0)	

n: Number of non-missing observations; %: Percentages for response categories based on total number of non-missing observations in the respective group, Percentages for missing observations are based on number of all subjects in the respective group; *: Chi-squared test.
Two participants had both a positive PCR test and a positive antibody test. These subjects had no acute symptoms but reported symptoms since March 2020. Due to the small number of positive cases, no subgroup analysis was performed.

Table 33: Positive and negative predictive values

	Train attendants	Train drivers	Maintenance workers	Total
	(%)	(%)	(%)	(%)
**IgG test**				
Prevalence	6.78	3.95	8.53	6.51
Positive predictive value	94.5	90.65	95.65	94.26
Negative predictive value	99.59	99.77	99.48	99.61

To calculate the positive and negative predictive values, a sensitivity of 94.4% and a specificity of 99.6% were assumed for the IgG test; the positive and negative predictive value was not calculated for the PCR test because a sensitivity and specificity of 1 were assumed.

Table 34: Antibody detection - 1st test series/3rd test series

		IgG test (3rd test series)					
		Positive		Negative		Missing	Total
	1st test series	n	(%)	n	(%)	n	n
**IgG test**	Positive	12	(66.7)	6	(33.3)	1	19
	Negative	32	(4.9)	626	(95.1)	13	671
	Missing	0		2		0	2
**PCR test**	Positive	0	(0.0)	0	(0.0)	0	0
	Negative	44	(6.5)	633	(93.5)	14	691
	Missing	0		1		0	1
**IgG or PCR test**	Positive	12	(66.7)	6	(33.3)	1	19
	Negative	32	(4.9)	625	(95.1)	13	670
	Missing	0		3		0	3
**Total**		44		634		14	692

n: Number of values in each category; %: Percentage based on test results of participants in 1st test series und 3rd test series.

Table 35: Antibody detection - 2nd test series/3rd test series

		IgG test(3rd test series)					
		Positive		Negative		Missing	Total
	2nd test series	n	(%)	n	(%)	n	n
**IgG test**	Positive	15	(93.8)	1	(6.3)	3	19
	Negative	39	(5.0)	742	(95.0)	9	790
	Missing	1		2		1	4
**PCR test**	Positive	1	(25.0)	3	(75.0)	0	4
	Negative	54	(6.8)	739	(93.2)	13	806
	Missing	0		3		0	3
**IgG or PCR test**	Positive	16	(80.0)	4	(20.0)	3	23
	Negative	38	(4.9)	736	(95.1)	9	783
	Missing	1		5		1	7
**Total**		55		745		13	813

n: Number of values in each category; %: Percentage based on test results of participants in 1st test series und 3rd test series.
